# Identifying the candidate genes involved in the calyx abscission process of 'Kuerlexiangli’ (*Pyrus sinkiangensis* Yu) by digital transcript abundance measurements

**DOI:** 10.1186/1471-2164-14-727

**Published:** 2013-10-23

**Authors:** Xiaoxiao Qi, Jun Wu, Lifen Wang, Leiting Li, Yufen Cao, Luming Tian, Xingguang Dong, Shaoling Zhang

**Affiliations:** College of Horticulture, State Key Laboratory of Crop Genetics and Germplasm Enhancement, Nanjing Agricultural University, Nanjing, 210095 China; Research Institute of Pomology, Chinese Academy of Agricultural Sciences, Xingcheng, 125100 China

**Keywords:** Pear, Calyx abscission, Digital transcript abundance measurements, Differentially expressed genes

## Abstract

**Background:**

'Kuerlexiangli’ (*Pyrus sinkiangensis* Yu), a native pear of Xinjiang, China, is an important agricultural fruit and primary export to the international market. However, fruit with persistent calyxes affect fruit shape and quality. Although several studies have looked into the physiological aspects of the calyx abscission process, the underlying molecular mechanisms remain unknown. In order to better understand the molecular basis of the process of calyx abscission, materials at three critical stages of regulation, with 6000 × Flusilazole plus 300 × PBO treatment (calyx abscising treatment) and 50 mg^.^L^-1^GA_3_ treatment (calyx persisting treatment), were collected and cDNA fragments were sequenced using digital transcript abundance measurements to identify candidate genes.

**Results:**

Digital transcript abundance measurements was performed using high-throughput Illumina GAII sequencing on seven samples that were collected at three important stages of the calyx abscission process with chemical agent treatments promoting calyx abscission and persistence. Altogether more than 251,123,845 high quality reads were obtained with approximately 8.0 M raw data for each library. The values of 69.85%-71.90% of clean data in the digital transcript abundance measurements could be mapped to the pear genome database. There were 12,054 differentially expressed genes having Gene Ontology (GO) terms and associating with 251 Kyoto Encyclopedia of Genes and Genomes (KEGG) defined pathways. The differentially expressed genes correlated with calyx abscission were mainly involved in photosynthesis, plant hormone signal transduction, cell wall modification, transcriptional regulation, and carbohydrate metabolism. Furthermore, candidate calyx abscission-specific genes, e.g. *Inflorescence deficient in abscission* gene, were identified. Quantitative real-time PCR was used to confirm the digital transcript abundance measurements results.

**Conclusions:**

We identified candidate genes that showed highly dynamic changes in expression during the calyx abscission process. These genes are potential targets for future functional characterization and should be valuable for exploration of the mechanisms of calyx abscission, and eventually for developing methods based on small molecule application to induce calyx abscission in fruit production.

**Electronic supplementary material:**

The online version of this article (doi:10.1186/1471-2164-14-727) contains supplementary material, which is available to authorized users.

## Background

The 'Kuerlexiangli’ pear is one of the characteristic and economically important fruit trees in Xinjiang Uygur Autonomous Region, China. Fruit of this cultivar is a rich source of juice and has few stone cells, good flavor, and tantalizing aroma. However, a persistent calyx seriously affects the fruit shape (Additional file 1) as well as quality. Fruit with calyx abscission have higher titer of soluble sugars and vitamin C, lower titer of titratable acids compared with persistent calyx fruit [1]. Thus calyx persistence significantly affects the commercial importance of 'Kuerlexiangli’ and causes serious loss in economic value.

Previously, few studies have focused on calyx abscission of pear, which is clearly regulated by ethylene, whilst auxins apparently decrease the ratio of calyx abscission [2, 3]. On the other hand, the rate of calyx abscission in 'Kuerlexiangli’ fruit is varied among different pollinizer varieties, with higher calyx abscission rates obtained when flowers were pollinated with 'Xueqing’, 'Yali’, 'Zhongliyihao’ and 'Cuiguan’ pear [4]. Moreover, manually excising the calyx was helpful for enhancing the comprehensive quality of pear fruit [5].

Recent research has mainly focused on the physiological aspects of the calyx development process, with emphasis on anatomy, and the role of nutrition and hormone control. Microstructure data of calyx tubes at the calyx developing stage after spraying 300 mg kg^-1^ paclobutrazol (PP333) (calyx abscising treatment) or 50 mg kg^-1^ GA_3_ (calyx persisting treatment) showed that the average area of vascular bundle of calyx tube tissue was bigger and many sieve tube cells and idioblasts gradually appeared when the tree was treated with GA_3_. When the tree was treated with PP333, calyx tubes of young fruit only had vessels in vascular bundle. Abscission layer appeared at the late young fruit of calyx tube developing stage, and finally the calyx tube broke off and young fruit developed as calyx fruit [6]. The physiological evidence indicates a role of both nutrition and hormone regulation in calyx abscission. Sufficient Fe content is a guarantee for fruit calyx development [7]. High content of zeatin-riboside (ZR) and indoleacetic acid (IAA) in young fruit, high content of IAA and GA_3_ but low content of abscisic acid (ABA) in calyx, high ratio of IAA, ZR, GA_3_ between calyx and young fruit, and low ratio of ABA between calyx and young fruit all promoted calyx persistence [8]. However, the key genes that control or regulate fruit calyx abscission or persistence are still unknown. Thus the understanding of gene expression and regulation differences under calyx abscission and calyx persistence status and the responses to different chemical agents leading to different calyx status is necessary.

Next generation sequencing methods have emerged as a cost-effective high-throughput approach to the sequencing of a very large number of expressed genes even in small experiments. One of these, digital transcript abundance measurements method is a revolutionary approach for expression analysis competing to replace microarrays for analyzing transcriptome [9]. It is tag-based transcriptome sequencing for measuring relative gene expression levels, which can identify, quantify, and annotate expressed genes on the whole genome level with or even without prior sequence knowledge. It enables an entirely new scale of biological experimentation to reveal related pathways or identify target genes involved in different bio-processes. For example, digital transcript abundance measurements approach has been used to study gene expression in the poplar (*Populus simonii* × *Populus nigra*) under salt stress [10]. To identify the candidate genes for sex determination of papaya (*Carica papaya*), papaya male, female, and hermaphrodite plants were used for digital transcriptome analysis using high-throughput serial analysis of gene expression [11]. The molecular regulation mechanism of the physiological and biochemical response to potassium starvation in soybean roots and shoots was investigated by high-throughput tag-sequencing [12]. Differentially expressed genes in cucumber (*Cucumis sativus* L.) root under waterlogging stress have also been identified by digital transcript abundance measurements [13]. Overall, the digital transcript abundance measurements method has provided more valuable tools for qualitative and quantitative gene expression analysis than the earlier microarray based assays [14].

Most current molecular knowledge on the abscission process has been obtained from the model plant *Arabidopsis thaliana*[15], as well as tomato (*Solanum lycopersicum*) [16]. The apple cluster during immature fruit drop also represents an ideal system to study the shedding of actively growing organs [17]. Recently developed molecular approaches have been used in abscission process in horticulture crops. Previous studies have identified the transcriptomes associated with flower abscission in tomato [18], differential gene expression by the cDNA microarray technique during abscission of citrus leaves under ethylene treatment [19] and fruitlet abscission in apple [17]. The differentially expressed genes have been identified during shedding of immature apple fruits have been identified with a cDNA-AFLP approach [20], and the role of NAA and shading in apple fruit abscission have been analyzed by the transcriptome method [21]. However, a deep knowledge of the molecular events occurring during the early phases of calyx abscission induction is still lacking. The new release of the whole genome sequence of Asian pear 'Dangshansuli’ [22], lays a good platform for genome-wide gene analysis. Here we report on the first use of genome-wide analysis to gain insight into the wide range of transcriptional responses associated with calyx abscission processes. Using Solexa/Illumina’s sequencing system, the transcriptomes were compared between chemical regulation of calyx abscission, 6000 × Flusilazole plus 300 × PBO inducing calyx abscission, and 50 mg^.^L^-1^ GA_3_ treatment to reduce calyx abscission. By investigating the expression of genes related to calyx abscission in 'Kuerlexiangli’, a number of pathways and candidate genes that are important in this process were identified.

## Results and discussion

### Effects of different treatments on calyx abscission rate

**Figure 1:**
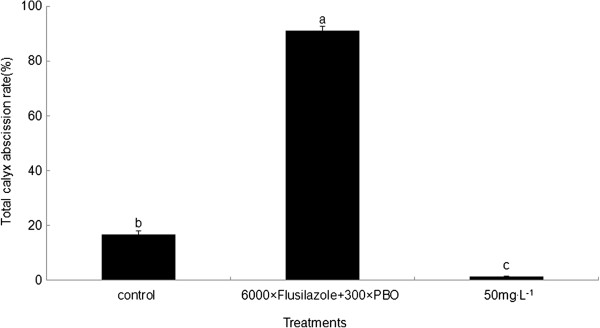
**Effect of Flusilazole and GA**_**3**_ **treatments on calyx abscission of 'Kuerlexiangli’ calyx.** 6000 × Flusilazole plus 300 × PBO increases the rate of calyx abscission, whereas GA_3_ decreases calyx abscission. Results represent the mean (±SD) of three replicates. Different letters indicate significant differences (*P* ≤ 0.05, Student *t*-test).

Comparison of Flusilazole treatment and GA_3_ treatment as inducer/inhibitor of fruit abscission revealed significant differences in abscission rates (Figure 1). At 22 d after treatments, the rate of calyx abscission in the untreated control was 16.78%, and the Flusilazole treatment increased the calyx abscission rate to 91.25%, but the GA_3_ treatment decreased the calyx abscission ratio to 1.38%. Thus the application of Flusilazole treatment increased the calyx abscission rate by 4.4 times whereas GA_3_ treatment decreased the calyx abscission rate to one fourteenth.

### Digital transcript abundance measurements libraries sequencing

Illumina sequencing analysis was performed to obtain a global view of the calyx abscission of 'Kuerlexiangli’ through analysis of the transcriptomes of Flusilazole treatment and GA_3_ treatment. Seven samples from C1 to C7 were used as material for digital transcript abundance measurements analysis (Table 1). Calyx digital transcript abundance measurements libraries (Table 2) were deep sequenced altogether. The number of reads for each library ranged from 7.1 to 9.9 million. A total of 8,132,745, 7,613,791, 7,370,972, 9,924,450, 7,174,967, 8,179,209, and 8,050,331 reads of raw data were obtained for C1, C2, C3, C4, C5, C6, and C7 libraries, respectively. In this study, the tag sequences of the seven digital transcript abundance measurements libraries were mapped to the assembled pear genome of 'Dangshansuli’ [22]. Finally, this generated 5,766,027, 5,381,267, 5,166,155, 6,932,208, 5,158,694, 5,653,121, and 5,511,540 reads corresponding to 70.90%, 70.68%, 70.09%, 69.85%, 71.90%, 69.12%, and 68.46% of all clean data in the seven libraries that could be mapped to the reference database. The Q30 percentages (A Q-score of 30 corresponds to an error rate of 1 per 1000) of all seven libraries were above 95%. All of these data showed that the throughput and sequencing quality was high enough for further analysis.

**Table 1:** **Description of calyx tube abscission zone samples used in this study**

Sample code	Description
Sample C1	Calyx tubes at 6 days after 6000 × Flusilazole + 300 × PBO treatment
Sample C2	Calyx tubes at 6 days after 50 mg/L GA_3_ treatment
Sample C3	Yellow loop in abscission zone from calyx tube at 10 days after 6000 × Flusilazole + 300 × PBO treatment
Sample C4	Calyx tubes at 10 days after treated with 50 mg/L GA_3_
Sample C5	Abscission zone from calyx tubes at 22 days after 6000 × Flusilazole + 300 × PBO treatment with calyx removing
Sample C6	Calyx tubes were still present at 22 days after 6000 × Flusilazole + 300 × PBO treatment
Sample C7	Calyx tubes were still present with control at the same time as in C6

**Table 2:** **Statistics of digital transcript abundance measurements library sequencing and tag mapping**

Samples	Raw data	Total length (bp)	Single length (bp)	Data mapping to gene	Mapping rate
C1	8132756	284646460	35	5766027	70.90%
C2	7613791	266482685	35	5381267	70.68%
C3	7370972	257984020	35	5166155	70.09%
C4	9924450	347355750	35	6932208	69.85%
C5	7174967	251123845	35	5158694	71.90%
C6	8179209	286272315	35	5653121	69.12%
C7	8132756	284646460	35	5766027	70.90%

### Analysis of differential gene expression

**Figure 2:**
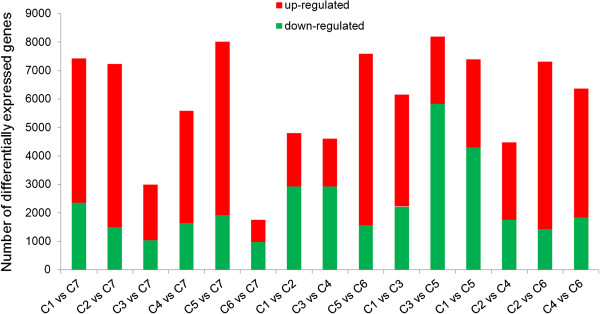
**Genes differentially expressed between different libraries.** Up-regulated (red) and down-regulated (green) genes were quantified. The results of 15 comparisons are shown.

To compare differential expression patterns among seven libraries, we employed IDEG6 (a web tool for detection of differentially expressed genes in multiple tag sampling experiments) to identify mRNAs showing statistically significant differences based on their relative abundance, as reflected by total count of individual sequence reads, between all pairs of libraries. We compared the six test libraries with the control, two treatments during the same stage, and the same treatment during different stages, so that 15 pairs of comparisons were implemented. Among these comparisons, we found that 1,760 to 8,184 genes had significant changes in expression, and the average number was 5,994 (Figure 2). The differential expression pattern among libraries revealed that the largest differences occurred between C3 and C5. A total of 8,184 genes were significantly differentially expressed between C3 and C5. Of these genes, 2,353 genes were up-regulated and 5,831 were down-regulated in C3 compared with C5. The smallest difference was observed between C6 and C7, in which only 1,760 differentially expressed genes (DEGs) were identified.

**Figure 3:**
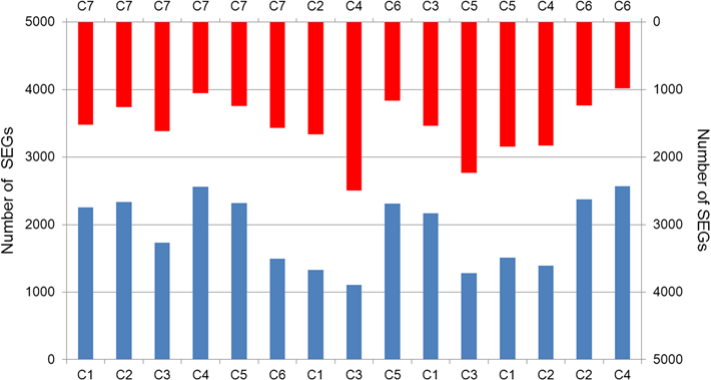
**Quantification of genes specifically expressed between different samples.** The numbers of digital transcript abundance measurements genes of 15 comparisons between each two samples are shown in red and blue histograms (top vs. bottom).

We also detected a large number of specifically expressed genes (SEGs) between each pair of libraries (Figure 3). Comparisons showed that there were 983 to 2,563 SEGs with an average number of 1,732 among 15 comparisons. These SEGs will help us to find genes correlated with calyx abscission process. Especially a number of stage-specific and treatment-specific expressed genes are likely to be key genes associated with calyx abscission.

### Functional annotation of differentially expressed genes

**Figure 4:**
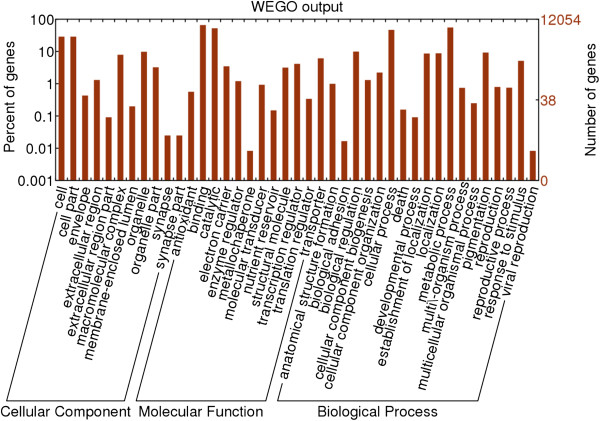
**GO categories of the genes identified.** Y-axis (left) represents percentages of genes identified in this study, Y-axis (right) represent the actual gene number. The genes were annotated in three main categories: biological process, cellular component, and molecular function (X-axis).

Gene Ontology (GO) is an international standardized gene function classification system that describes properties of genes and their products in any organism. In this study, a total of 12,054 differentially expressed genes that could be categorized into 41 functional groups were found (Figure 4). The major subcategories were as follows: four subcategories for cellular component ('cell’, 'cell part’, 'organelle’, and 'macromolecular complex’); three subcategories for molecular function ('binding’, 'catalytic activity’, and 'transporter activity’); and seven subcategories for biological process ('metabolic process’, 'cellular process’, 'biological regulation’, 'pigmentation’, 'localization’, 'establishment of location’, and 'response to stimulus’). These results indicate that expressed genes functioning in 'binding’, 'catalytic activity’, 'metabolic process’, and 'cellular process’ are important during the calyx abscission process. Only a few genes were clustered in terms of 'synapse’, 'synapse part’, 'metallochaperone activity’, 'biological adhesion’ and 'immune system process’ and 'viral reproduction’.

To further investigate the function of different expressed genes during calyx abscission, significantly enriched Kyoto Encyclopedia of Genes and Genomes (KEGG) pathways were identified according to the *P* values and enrichment factor. Performing a BLAST search against the KEGG database indicated that expressed genes were involved in 251 pathways (Additional file 2). As shown in Additional file 3, nine KEGG pathways were observed to be significantly overrepresented in calyx abscission processes. Those genes correlated with calyx abscission mainly involved in photosynthesis, plant hormone signal transduction, cell wall modification, transcriptional regulation and carbohydrate metabolism were used for subsequent analysis. These trends were consistent with overall developmental activities during abscission processes [23]. In addition, several other biological processes that have not previously been reported to be associated with calyx abscission, such as flavonoid biosynthesis and flavone/flavonol biosynthesis, were found dramatically changed during calyx abscission processes. These might be novel genes that are relevant to the calyx abscission process in 'Kuerlexiangli’ fruit.

### Cluster of calyx abscission-related genes

#### Impacts on photosynthesis

Decrease in photosynthesis may be an important contributing factor for the abscission of flowers and fruitlets in the abscission processes [24], which was confirmed by our present experiment. Our results showed that 230 genes encoding photosynthesis-related genes were differential expression under different treatments (Additional file 4). Altered expressions were found for numerous genes involved in carbon fixation, photosystem I, photosystem II, and photosynthetic electron transport. Flusilazole treatment decreased the expression of genes involved with PSI, PSII activity and photosynthetic electron transport in photosystem. The down-regulated genes included those that encode subunit V, PsaA/PsaB, F-type H^+^-transporting ATPase subunit gamma in photosystem I, and 13 kDa protein in photosystem II (Table 3). We identified that GA_3_ treatment led to strong induction in photosynthesis-related gene expression (Additional file 4). These results indicate that photosynthesis might play an important role in the calyx abscission and samples with diminished photosynthesis function are more prone to abscission.

**Table 3:** **Selected genes with altered expression during calyx abscission process**

Functional group	Gene ID	Gene description	TPM (transcripts per million clean tag)						
			C1	C2	C3	C4	C5	C6	C7
Photosynthesis									
	Pbr041327.1	Photosystem I subunit V	62.76	208.63	58.71	123.22	106.36	69.55	42.42
	Pbr001999.2	Photosystem I PsaA/PsaB	1.48	1.74	5.07	12.53	4.54	30.45	20.26
	Pbr020992.1	Photosystem I PsaA/PsaB	0.62	0.80	2.82	6.942	3.08	16.94	10.13
	Pbr031897.1	F-type H + -transporting ATPase subunit gamma	17.17	49.15	12.11	41.26	32.20	17.06	16.84
	Pbr032910.1	Photosystem II 13 kDa protein	14.46	30.59	4.37	15.52	15.82	10.07	8.23
Hormone/signaling									
	Pbr031954.1	ACC oxidase 7	9.27	14.43	71.38	86.68	17.61	7.23	7.72
	Pbr002199.1	Ethylene receptor	55.97	114.60	40.13	30.56	117.91	36.02	28.87
	Pbr023072.1	Ethylene receptor	43.61	131.70	63.22	32.01	91.77	43.13	33.68
	Pbr036692.1	ACC synthase	38.30	18.83	11.12	15.04	29.58	3.91	4.05
	Pbr000435.1	Ethylene-responsive transcription factor	14.83	4.41	3.24	8.20	9.78	2.37	4.18
	Pbr025988.1	Ethylene-responsive transcription factor	0.62	0	0	0.58	1.79	0	0
	Pbr030542.1	Ethylene-responsive transcription factor 1B	4.82	2.27	2.11	35.67	12.25	2.84	4.94
	Pbr021608.1	PtrAUX6; auxin influx carrier component	1.73	6.41	2.25	10.32	29.44	5.33	1.77
	Pbr013424.1	PIN6; auxin efflux carrier component	111.57	52.76	32.52	89.18	27.93	6.28	19.50
Cell wall modification									
	Pbr036382.1	Polygalacturonase	17.67	1.60	0.28	25.84	2.06	0.47	0.13
	Pbr014899.1	Pectinesterase	7.17	2.27	1.27	0	0	0	0.25
	Pbr014900.1	Pectinesterase	6.18	2.54	1.41	0	0.28	0.12	0
	Pbr033313.1	Beta-expansin 1a	163.83	69.86	189.37	93.14	147.49	139.93	167.78
	Pbr035305.1	Pectate lyase	3.34	5.74	2.11	5.01	20.78	1.54	1.52
	Pbr022719.1	Pectinesterase-2	9.02	2.14	0.28	2.41	1.65	0	0
	Pbr011421.1	Alpha-expansin 15	7.66	3.21	0.85	3.95	6.88	2.13	2.41
	Pbr005280.1	Pectate lyase	5.81	0.94	1.55	17.55	9.50	1.54	0.63
	Pbr039133.1	Pectate lyase	10.39	4.01	1.83	0.29	0.28	0.24	0.38
	Pbr000187.1	Xyloglucan endotransglucosylase/hydrolase	1.48	2.81	1.27	2.12	5.09	0.24	0.25
	Pbr042842.1	Xyloglucan endotransglucosylase/hydrolase	6.18	6.28	2.53	4.34	11.01	1.30	0.89
	Pbr040203.1	xyloglucan endotransglycosylase	6.18	5.61	3.66	3.76	4.95	1.42	1.14
Transcription factors									
	Pbr029902.1	AP2 domain-containing transcription factor	0.49	2.54	1.41	3.18	10.59	2.61	0.89
	Pbr022669.1	MYB 12 transcription factor	1.24	1.74	0.28	1.83	4.27	1.42	1.14
	Pbr002230.1	WRKY transcription factor	36.82	6.55	0.42	11.96	6.33	0.47	36.82
	Pbr036688.1	WRKY transcription factor	38.18	6.81	0.42	12.44	6.47	0.47	38.18
	Pbr010864.1	SCL domain class transcription factor	873.50	401.50	374.51	173.06	78.7	144.67	210.83
Carbohydrate biosynthesis and metabolism									
	Pbr003011.1	Alpha-amylase	293.80	76.67	33.09	255.11	32.06	24.88	25.96
	Pbr008092.1	Sugar transporter	225.35	58.90	299.04	149.06	74.02	104.74	167.15
	Pbr013916.1	Sorbitol dehydrogenase,	25.45	19.23	61.67	45.99	20.09	44.31	73.07
	Pbr032770.1	Sorbitol dehydrogenase	1.73	0.80	0.28	1.64	0.55	0.12	0.63
	Pbr013912.1	Sorbitol dehydrogenase	9.88	13.49	14.50	60.07	42.93	28.91	15.96
	Pbr013913.1	Sorbitol dehydrogenase	177.79	73.46	73.92	187.82	174.87	59.48	110.42
	Pbr001279.1	UDP-glucosyltransferase|	57.95	22.17	30.13	14.46	17.20	6.87	11.14
	Pbr019918.1	Glycosyl Hydrolase	51.03	4.01	5.91	8.58	8.67	2.73	2.15
	Pbr001745.1	Sucrose phosphate syntase	1.73	4.274	6.476	1.928	6.742	2.133	1.773
	Pbr008035.1	Sucrose phosphate syntase	2.842	7.88	10.7	4.435	11.97	3.555	3.546
	Pbr014962.1	Glycosyltransferase QUASIMODO1	45.34	67.32	20.98	35.87	76.09	33.41	21.78
	Pbr033075.1	Glycosyltransferase QUASIMODO1	61.28	88.15	26.19	44.54	100.58	38.51	22.03
	Pbr036396.1	Glucose-1-phosphate adenylyltransferase	49.54	42.47	23.37	43.10	54.35	24.17	26.34
	Pbr005718.1	Beta-fructofuranosidase	10.13	3.47	0.70	0.39	0.69	0	0.89
Other differentially regulated genes									
	Pbr009422.1	Probable E3 ubiquitin-protein ligase	8.15	1.74	27.60	0.87	9.22	2.01	2.91
	Pbr039793.1	Acyltransferase	45.01	11.62	17.60	7.91	20.64	3.32	4.31
	Pbr032411.1	Cysteine-rich receptor-like protein kinase	28.91	7.88	9.86	28.06	4.13	0.59	1.14
	Pbr013236.1	Protein IDA	60.88	13.89	9.29	32.01	3.16	0	0.38
	Pbr012802.1	Predicted protein	5.19	2.00	7.88	2.41	4.27	1.19	1.52
	Pbr012908.1	Predicted protein	4.20	1.34	7.74	2.41	11.56	2.84	1.01
	Pbr042050.1	Predicted protein	194.34	41.00	13.37	143.47	5.78	0.83	1.52
	Pbr020727.1	Unknown	546.95	188.06	250.61	93.23	87.78	245.15	177.53

#### Plant hormone signal transduction

Many kinds of hormones regulate the process of abscission, such as IAA, ABA, GA, JA, and ethylene, among which IAA and ethylene play a pivotal role. IAA prevents, while ethylene accelerates the abscission processes [25]. Forty-three genes were found to be involved in ethylene synthesis, perception, and response in the present study. ACC synthase (ACS) and ACC oxidase (ACO) are rate-limiting enzymes in ethylene biosynthesis [26, 27]. Genes involved in ACC synthase (Pbr002233.1, Pbr007947.1, Pbr032688.1, Pbr001095.1, Pbr029891.1, Pbr030234.1) showed expressions increased by 2.109 to 6.236 times during the early calyx abscission process after Flusilazole treatment. Two ACC oxidase genes (Pbr005179.1 and Pbr031954.1) were up-regulated in later calyx abscission process after Flusilazole treatment. On the other hand, two ethylene receptor genes (Pbr002199.1 and Pbr023072.1) and three ethylene responsive genes (Pbr000435.1, Pbr025988.1, and Pbr030542.1) were up-regulated in later calyx abscission process after Flusilazole treatment (Additional file 4). This is consistent with the correlation between abscission and increased expression of genes for ethylene synthesis and ethylene receptors in AZ (abscission zone), which has been reported in mature fruit of olive [28] and apple [21]. The general rule states that provided the flux of IAA to the abscission zone is maintained, cell separation is inhibited and abscission does not happen [29]. One hundred and two auxin-related genes were differentially expressed by the Flusilazole and GA_3_ treatments, including indole-3-acetic acid-amido synthetase, indole-3-acetic acid-induced protein, and auxin response factor. Nine genes encoding AUX/IAA protein were down-regulated at 6 d after Flusilazole treatment and 13 genes encoding auxin responsive factors were also down-regulated at 10 d after Flusilazole treatment. Genes related to polar auxin transporting were also affected by GA_3_ treatment, with auxin influx carriers induced and efflux carriers largely repressed. We found that an auxin transport-related gene (Pbr013424.1) was up-regulated at 6 d, but repressed at 10 d after Flusilazole treatment. In addition, a gene (Pbr011705.1) encoding spermidine synthase was up-regulated with Flusilazole treatment at onset of calyx abscission processes. As mentioned above, hormones seem to play a relatively important role during the early phases of abscission in the calyx, since a majority of the transcriptionally activated elements involved in hormone signaling seem to be downstream of the induction of abscission. It is especially interesting that expression of more genes associated with ethylene and auxin metabolism and signal transduction were altered during calyx expression process than previously reported.

#### Cell wall degradation-related genes are highly expressed in calyx process

Abscission involves progressive dissolution of the middle lamella. A key step in loss of adhesion between cells within a separation layer is the induction of cell wall-degrading enzymes such as polygalacturonases. The role of other wall-modifying proteins such as expansin, xyloglucan transglucosylase hydrolase, pectinesterase, etc. has also been studied during abscission processes [30]. Polygalacturonases have been studied in tomato abscission zones [31], in oilseed rape and *Arabidopsis* leaf and flower abscission zones [32], while celluloses have been studied in cotton [33], citrus fruit [34] and red raspberry abscission zones [35]. Reports have indicated that an increase in expansin [36], xyloglucan transglucosylase hydrolases [37] and pectate lyases [38] correlate with organ abscission. A large number of genes encoding cell wall hydrolases, expansin, and lyases were found to be over-represented with Flusilazole treatment (Additional file 4). In this study, a gene (Pbr036382.1) encoding polygalacturonase and a gene (Pbr014900.1) encoding pectinesterase were up-regulated at 22 d after Flusilazole treatment. We also observed expansin expression in calyx abscission process. Three genes (Pbr000187.1, Pbr042842.1, and Pbr040203.1) encoding xyloglucan endotransglucosylase/hydrolase were up-regulated at 22 d after Flusilazole treatment (Table 3). Pectate lyases (PLs,EC:4.2.2.2) could facilitate cell wall disassembly by their action on demethylated pectin through beta elimination in the presence of Ca^2+^, leading to depolymerization of pectic polysaccharides [39]. Some candidate genes related with this reaction identified in this study may play roles in cell wall degradation, such as Pbr011950.1, Pbr021157.1, Pbr035305.1, and Pbr041275.2, all of which probably aid in later abscission processes.

#### Genes encoding transcription factors

Transcription factors (TFs) act as major switches of regulatory cascades during development, and alterations in the expression of such genes may affect various developmental processes. Our digital transcript abundance measurements results showed that 198 genes encoding transcription factors were differentially expressed, including ERF/APETALA2, MYB, and WRKY transcription factors, and MADS-box proteins (Additional file 4). APETALA2 (AP2) is a floral homeotic factor that plays an important role in the control of *Arabidopsis* flower and seed development and encodes a putative TF that is distinguished by a novel DNA-binding motif referred to as the AP2 domain [40]. The expression of a gene (Pbr029902.1) encoding for a TF containing an AP2 domain was transiently up-regulated specifically at a later stage under GA_3_ treatment. Multiple up-regulated MYB factors at 22 d after Flusilazole treatment were identified, including *MYB*105, 160, 220, 219, 12, and 190. Moreover, *MYB*12 is a flavonol-specific activator of flavonoid biosynthesis, strongly triggering the promoters of genes encoding chalcone synthase (CHS), flavanone-3-hydroxylase (F3H), and flavonol synthase (FLS), which are all involved in the biosynthesis of flavonols [41, 42]. The up-regulation of *MBY*12 (Pbr022669.1) consistently resulted in an enhanced transcript level of CHS at 22 d after Flusilazole treatment. Interestingly, auxin and ethylene induce flavonol accumulation through partly identical transcription networks [43]. We hypothesize that the calyx abscission zone contains higher levels of flavonols and flavonoids, which might have functions in stress defense after calyx abscission. The WRKY family is a superfamily of TFs, which hold central positions mediating fast positive and negative regulation of disease resistance [44]. Two *WRKY* genes were up-regulated transiently at 6 d after Flusilazole treatment (Table 3), which showed similar change pattern to that reported in tomato flower abscission zone [18]. On the whole, all above results suggest that many transcription factors play an important role in regulating calyx abscission processes in pear.

#### Genes involved in carbohydrate metabolism

A strong connection between the carbohydrate amounts available for the fruitlet, especially soluble sugars, and their probability of abscission has been suggested [45]. This phenomenon has been also described for pistachio [46]. So abscission may be due to a lack of carbohydrates. In our digital transcript abundance measurements results, 486 carbohydrate metabolism genes had significantly altered expression profiles in response to Flusilazole treatment and GA_3_ treatment (Additional file 4). Affected genes within this group include those associated with glycolysis/gluconeogenesis, fatty acid biosynthesis, and sucrose metabolic processes. For example, we found induction of alpha-amylase (EC: 3.2.1.1), beta-fructofuranosidase (EC: 3.2.1.26), alcohol dehydrogenase (EC: 1.1.1.1), and acetyl-CoA carboxylase (EC: 6.4.1.2) under Flusilazole treatment. A different gene set showed lower expression in calyx abscission. This set comprised genes coding for 2-isopropylmalate synthase (EC: 2.3.3.13), pyruvate decarboxylase (EC: 4.1.1.1), sorbitol dehydrogenase (EC: 1.1.1.14) and ribulose-1,5-bisphosphate carboxylase (EC: 4.1.1.39). Some of the genes are clearly connected to sugar mobilization, such as alpha-amylase and glycosyl hydrolase, which were up-regulated at 6 d after Flusilazole treatment. The gene (Pbr001279.1) encoding UDP-glucosyltransferases (EC2.4.1.115) was highly expressed under Flusilazole treatment in the abscission process (Table 3). In apple, UDP-glucosyltransferases were also expressed during fruit abscission [17]. Genes related to sucrose metabolism, e.g., sucrose-phosphate synthase (Pbr001745.1, Pbr008035.1), glycosyltransferase (Pbr014962.1 and Pbr033075.1), and glucose-1-phosphate adenyltransferase (Pbr036396.1) were up-regulated under GA_3_ treatment during the later calyx abscission process, while beta-fructofuranosidase was up-regulated under Flusilazole treatment at the onset of calyx abscission process. Levels of sugars like galactose, fructose, and glucose, as well as transcripts for their transporters, increase in senescing leaves analogously to what happens under environmental stresses, such as cold and dehydration [47]. A gene encoding a sugar transporter was up-regulated with Flusilazole treatment at the onset of the calyx abscission processes observed for Pbr008092.1. Sorbitol dehydrogenase (SDH) has been identified as a key enzyme in sorbitol metabolism, converting sorbitol into fructose [48, 49]. In this study, four genes encoding SDH (Pbr013916.1, Pbr032770.1, Pbr013912.1, and Pbr013913.1) were down-regulated with Flusilazole treatment in later calyx abscission processes, suggesting that sorbitol catabolism was largely inhibited, resulting in abscission.

#### Other differentially regulated genes

There were other genes that showed high-level differential expression related to calyx abscission (Table 3). E3 ubiquitin-protein ligase has been shown to play an important role in hormone regulation, photomorphogenesis, floral homeosis, senescence, and pathogen defense in plant [50]. Expression of Pbr009422.1, encoding E3 ubiquitin-protein ligase, was up-regulated under Flusilazole treatment during the calyx abscission processes. Expression of Pbr039793.1, encoding acyltransferase, was up-regulated in late calyx abscission processes. One gene (Pbr032411.1) encoding cysteine-rich receptor-like protein kinase 26-like was identified. Cysteine-rich receptor-like kinases is one of the largest groups of receptor-like kinases, which have been suggested to play important roles in the regulation of pathogen defense and programmed cell death [51]. It is notable that the gene Pbr013236.1 was up-regulated with Flusilazole treatment in both early and late calyx abscission processes; it showed 69% identity with IDA (Inflorescence deficient in abscission) protein of *Arabidopsis*, which is associated with regulation of floral organ abscission. *IDA* encodes a small protein with an N-terminal signal peptide. Analysis of *ida* mutant plants indicates that *IDA* regulates floral organ abscission through an ethylene insensitive pathway. Overexpression of *IDA* results in early abscission and production of arabinose and galactose in the floral AZs [15]. This suggests that the activity of *IDA* may be important to the onset and later stages of the calyx abscission process. However, further functional experiments are necessary to confirm this point. In addition, some differentially expressed genes (Pbr012802.1, Pbr012908.1 Pbr042050.1, and Pbr020727.1) without annotation were also found. We hypothesize that these genes are putative calyx abscission-related transcripts.

### Confirmation of differentially expressed genes by qRT-PCR

In order to verify the genes that were actually differentially expressed during the calyx abscission processes, the expressive abundance of seven selected genes was analyzed by quantitative real-time PCR. The results showed that although the exact fold changes of six of the selected genes at several data points varied between digital transcript abundance measurements and qRT-PCR analysis, trends of gene expression change detected by the two different approaches were largely consistent. Only one gene (Pbr000187.1) did not show consistent expression between accurate quantification of expression and digital transcript abundance measurements (Additional file 5). Pearson’s correlation coefficient (r) showed that both the digital transcript abundance measurements and qRT-PCR data were highly correlated, with the r-value ranging from 0.656 (Pbr036692.1) to 0.934 (Pbr001279.1), which was in agreement with previous report [52]. The qRT-PCR further demonstrated that genes related to photosystem reaction (Pbr041327.1), hormone-related transcripts (Pbr036692.1), carbohydrate metabolism (Pbr021608.1, Pbr008092.1, and Pbr001279.1) and other differentially regulated genes (Pbr027181.1) showed significant difference between treatments and participated in the process of calyx abscission or persistent processes.

## Conclusions

The present results have demonstrated the usefulness of the digital transcript abundance measurements approach to identify differentially expressed genes between Flusilazole treatment and GA_3_ treatment. These differentially expressed genes may well be important for calyx abscission in fruit. In addition, a list of candidate target genes for functional studies involving calyx abscission process was generated. Among the isolated candidate genes, *IDA* appears to play a key role during calyx abscission processes. Further studies should be concentrated on functional characterization of these genes in the future. This study could lead to better understanding of the molecular mechanism of the phenotypic difference between calyx abscission and persistent fruits. Moreover, the findings of this study may facilitate the selection of new chemical agents and accelerate genetic strategies for the development of more effective pear calyx abscission for commercial purposes.

## Methods

### Plant materials and treatments

The plant materials used in this study were obtained from the Research Institute of Pomology, Chinese Academy of Agricultural Sciences (CAAS), Xingcheng, Liaoning province during the 2012 growing season. Five uniform fifty-year-old 'Kuerlexiangli’ trees were selected and divided into three blocks of six branches each. Two branches from each block were treated with: 6000 × Flusilazole + 300 × PBO (calyx abscising treatment) or GA_3_ 50 mg^.^L^-1^ (calyx persisting treatment) sprayed at 0 d after full bloom, with plants with no treatment as the control. Under these treatments, calyx tube abscission symptoms (yellow loop in abscission zone) were observed within 10 d and only a few abscission symptoms were found with calyx persisting treatment. Therefore, we selected two early time points after treatment (6 and 10 d) in order to detect genes responsible for the early abscission events. An additional sample was collected 22 d after the treatment time point because at that stage, no further calyx abscission occurs. Samples of the two treatments at defined time points were collected for digital transcript abundance measurements (Table 1). A pear fruit with calyx tube at 22 days after full bloom is shown in Additional file 6. A total of seven independent libraries were sequenced. At each time point, about 100 fruits were collected from each branch. The calyx abscission zone (AZ) tissues, containing a few layers of AZ cells on the proximal side of the separation line and adjacent cells, were manually dissected from the calyx tube samples, using a razor blade of 1 mm^3^. The AZ tissues were collected and frozen in liquid nitrogen and kept at -80°C until RNA isolation.

### Field test of calyx abscission rate induced by different chemical agents

To determine the calyx abscission rate, all flowers on branches marked at the beginning of experiment, were counted and recorded at 22 d after full bloom in different treatments. Calyx abscission rate = number of fruits with calyx abscission/ number of all fruits tested.

### RNA isolation and Solexa/Illumina sequencing

Solexa/Illumina sequencing was carried out by CapitalBio Corporation, Beijing, China. The total RNA was extracted from the samples using Plant RNA Isolation Kit (AutoLab), followed by RNA purification with RNeasy MiniElute Cleanup Kit (Qiagen), according to the manufacturer’s instruction. Total RNA content, purity and degradation were assessed by Nanodrop2000 spectrophotometer (Thermo scientific, USA) and quality of RNA was confirmed by agarose gel electrophoresis before proceeding. For mRNA library construction and deep sequencing, RNA samples were prepared using the TruSeq RNA Sample Preparation Kit according to the manufacturer’s protocol. Briefly, the poly-A-containing mRNA molecules were purified from 3 μg of total RNA using poly-T oligo-attached magnetic beads with two rounds of purification. For the second round elution of the poly-A RNA, the RNA was fragmented using divalent cations under 95°C. For Solexa/Illumina sequencing, cDNA synthesis was carried out with the broken RNA fragments and these RNA fragments reversely transcribed into first strand cDNA using random hexamers. Second-strand cDNA synthesis using DNA Polymerase I and RNase H. The cDNA fragments were put through an end repair process to convert the overhangs into blunt ends using an End Repair (ERP) mix. The 3′ to 5′ exonuclease activity of this mix removes the 3′ overhangs and the polymerase activity fills in the 5′ overhangs. A single 'A’ nucleotide was then added to the 3′ ends of the blunt fragments to prevent them from ligating to one another during the adapter ligation reaction. A corresponding single 'T’ nucleotide on the 3′ end of the adapter provides a complementary overhang for ligating the adapter to the fragment. This strategy ensures a low rate of chimera (concatenated template) formation. The multiple indexing adapters were ligated to the ends of the double-stranded cDNA, making them for hybridization onto the Illumina Sequencing Chip (flow cell). PCR was used to selectively enrich those DNA fragments that have adapter molecules on both ends and to amplify the amount of DNA in the library, and was minimized to twelve cycles to avoid skewing the representation of the library. A gel purification procedure was carried out to select the fragments sized from 300 to 400 bp to produce the library for cluster generation and sequencing. The libraries were checked for quality by Agilent 2100 bioanalyzer and quantified by Qubit and qPCR. Cluster formation and sequencing on the GAIIx platform were performed following the manufacturer’s standard cBot and sequencing protocols. For the multiplexing sequencing, 35 cycles of single read were used to sequence the RNA, followed by 7 cycles of index identification.

### Data analysis

Primary data analysis and base calling were performed using the Illumina instrument software. The following sequencing data were excluded from the analysis: low quality sequences such as the 3’ adaptor sequence; tags which were too long or too short; tags with unknown sequence; single copy tags. The remaining high quality sequences (clean data) were mapped to the pear gene set [22] using the software tool Bowtie [53]. A Perl script was written to process the mapping results and generate the gene expression profile. Similar to credibility interval approaches reported for the analysis of SAGE data [54], we employed IDEG6 [55] to identify mRNAs showing statistically significant differences based on their relative abundance (as reflected by total count of individual sequence reads) between the two libraries. The general chi-square test was performed, as it has been proven to be one of the most efficient tests (http://bioservices.capitalbio.com/xzzq/rj/3885.shtml). Finally, genes with a P value < = 0.01 and Fold Change > =2 were marked significantly different between the two libraries. InterPro domains [56] were annotated by InterProScan [57] Release 36.0 and functional assignments were mapped onto Gene Ontology (GO) [58]. Furthermore, the GO classification and draw GO tree using WEGO [59]. Genes were mapped to terms in the Kyoto Encyclopedia of Genes and Genomes database (KEGG, release [60]) using BLASTX [61] at E values < = 1e-10. A Perl script was used to retrieve KO (KEGG Ontology) information from the blast search result and then establish pathway associations between unigene and database.

### Real-time PCR

To validate the expression patterns revealed by digital transcript abundance measurements results, seven genes identified through digital transcript abundance measurements were analyzed using quantitative real-time PCR. The RNA samples for digital transcript abundance measurements were also used for quantitative real-time PCR. Primer sequences for the real-timer PCR assay were designed using eprimer 3 (from the program EMBOSS Explorer) and listed in Additional file 7. Total RNA was treated with DNase I to remove genomic DNA contamination. Approximately 1 μg of total RNA was used as a template for reverse transcription using ReverTra Ace-αFirst Strand cDNA Synthesis Kit (TOYOBO,TOYOBO Biotech Co. Ltd, Japan ) according to the manufacturers’ instructions. The qRT-PCRs were performed using SYBR® Green Premix kit (TaKaRa Biotechnology. Dalian, China). The composition of PCR mix was as follows: 10 μl 2 × SYBR Premix ExTaq™, 0.4 μl each primer and 1μl of cDNA template in a final volume of 20 μl. All reactions were run as duplicates in 96-well plates. The qRT-PCR was performed on the Lightcycle-480 (Roche). Each cDNA was analyzed in triplicate, after which the average threshold cycle (Ct) was calculated per sample. The relative expression levels were calculated with the 2^-ΔΔCt^ method [62]. Tubulin (AB239681) was used as the internal control. The primer pairs were: forward: 5′ TGGGCTTTGCTCCTCTTAC 3′; reverse: 5′ CCTTCGTGCTCATCTTACC 3′. The protocol of real-time PCR was as follows: initiation with a 10 min denaturation at 95°C followed by 40 cycles of amplification with 15 s of denaturation at 95°C, 15 s of annealing at 60°C, 20 s of extension at 72°C and reading the plate for fluorescence data collection at 60°C. A melting curve was performed from 60 to 95°C to check the specificity to the amplified product.

## Electronic supplementary material

Additional file 1: **Pear fruit without calyx (A) and pear fruit with calyx (white arrow) (B) during the fruit mature period.** (DOC 758 KB)

Additional file 2: **Pathway enrichment analysis of differentially expressed genes.** A BLAST search against the KEGG database indicated that expressed genes from C1 to C7 were involved in 251 pathways. (DOC 220 KB)

Additional file 3: **List of significantly altered pathways with enriched differentially expressed genes after Flusilazole treatment and GA**_**3**_ **treatment.** The table provides the list of significantly alter biochemical pathways during calyx abscission process: Pathway ID, pathway name, the total number of genes with pathway annotation, the number of differentially expressed genes with pathway annotation of each comparison (Fifteen pairs comparison in total in this table, e.g. C1 vs C7 indicates the difference expressed genes in the comparison between C1and C7). (DOC 44 KB)

Additional file 4: **Categorization of significant genes encoding enzymes and proteins with a variety of biological functions.** In this table, five functional categories of genes showed differential expression patterns after Flusilazole treatment and GA_3_ treatment. The details of gene expression analysis of 15 pairs of comparisons: Gene ID, gene description and TPM (transcripts copies per million tags) of genes. (DOC 2 MB)

Additional file 5: **Real-time quantitative PCR analysis of selected differential genes detected via digital transcript abundance measurements.** Representative genes selected for the analysis were those involved in photosynthesis, plant hormone signal transduction, carbohydrate metabolism, cell wall degradation, and other processes. (DOC 88 KB)

Additional file 6: **A pear fruit with calyx tube (white arrow) at 22 d after full bloom.** (DOC 764 KB)

Additional file 7: **Primer details for genes selected for quantitative real-time PCR analysis from results of digital transcript abundance measurements.** This is the primer list of seven genes selected for quantitative real-time PCR assay to confirm the reliability of digital transcript abundance measurements. Gene ID, forward and reverse primers are shown. (DOC 30 KB)
