# Supplementary material for: Identifying the candidate genes involved in the calyx abscission process of 'Kuerlexiangli’ (Pyrus sinkiangensis Yu) by digital transcript abundance measurements
Source: BMC Genomics. 2013 Oct 23;14(1):727. doi: 10.1186/1471-2164-14-727 (PMC4046677; doi:10.1186/1471-2164-14-727)
Supplement: Supplementary file 1 — Additional file 1: Pear fruit without calyx (A) and pear fruit with calyx (white arrow) (B) during the fruit mature period. (DOC 758 KB) [file 12864_2013_5444_MOESM1_ESM.doc]

**Gene Additional file 1: Pear fruit without calyx (A) and pear fruit with calyx (white arrow) (B) during the fruit mature period.**

**
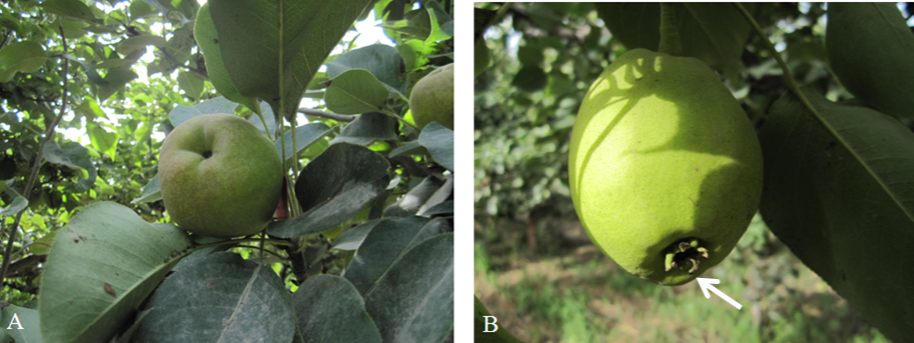
**
