# Supplementary material for: Identifying the candidate genes involved in the calyx abscission process of 'Kuerlexiangli’ (Pyrus sinkiangensis Yu) by digital transcript abundance measurements
Source: BMC Genomics. 2013 Oct 23;14(1):727. doi: 10.1186/1471-2164-14-727 (PMC4046677; doi:10.1186/1471-2164-14-727)
Supplement: Supplementary file 2 — Additional file 2: Pathway enrichment analysis of differentially expressed genes. A BLAST search against the KEGG database indicated that expressed genes from C1 to C7 were involved in 251 pathways. (DOC 220 KB) [file 12864_2013_5444_MOESM2_ESM.doc]

| **Additional file 2: Pathway enrichment analysis of differentially expressed genes.** A BLAST search against the KEGG database indicated that expressed genes from C1 to C7 were involved in 251 pathways. |
| --- |
| **Pathway** |
| >1. Metabolism |
| Metabolism; Amino Acid Metabolism; Alanine, aspartate and glutamate metabolism [PATH:ko00250]; |
| Metabolism; Amino Acid Metabolism; Amino acid related enzymes [BR:ko01007]; |
| Metabolism; Amino Acid Metabolism; Arginine and proline metabolism [PATH:ko00330]; |
| Metabolism; Amino Acid Metabolism; Cysteine and methionine metabolism [PATH:ko00270]; |
| Metabolism; Amino Acid Metabolism; Glycine, serine and threonine metabolism [PATH:ko00260]; |
| Metabolism; Amino Acid Metabolism; Histidine metabolism [PATH:ko00340]; |
| Metabolism; Amino Acid Metabolism; Lysine biosynthesis [PATH:ko00300]; |
| Metabolism; Amino Acid Metabolism; Lysine degradation [PATH:ko00310]; |
| Metabolism; Amino Acid Metabolism; Phenylalanine metabolism [PATH:ko00360]; |
| Metabolism; Amino Acid Metabolism; Phenylalanine, tyrosine and tryptophan biosynthesis [PATH:ko00400]; |
| Metabolism; Amino Acid Metabolism; Tryptophan metabolism [PATH:ko00380]; |
| Metabolism; Amino Acid Metabolism; Tyrosine metabolism [PATH:ko00350]; |
| Metabolism; Amino Acid Metabolism; Valine, leucine and isoleucine biosynthesis [PATH:ko00290]; |
| Metabolism; Amino Acid Metabolism; Valine, leucine and isoleucine degradation [PATH:ko00280]; |
| Metabolism; Biosynthesis of Other Secondary Metabolites; Anthocyanin biosynthesis [PATH:ko00942]; |
| Metabolism; Biosynthesis of Other Secondary Metabolites; Butirosin and neomycin biosynthesis [PATH:ko00524]; |
| Metabolism; Biosynthesis of Other Secondary Metabolites; Caffeine metabolism [PATH:ko00232]; |
| Metabolism; Biosynthesis of Other Secondary Metabolites; Flavone and flavonol biosynthesis [PATH:ko00944]; |
| Metabolism; Biosynthesis of Other Secondary Metabolites; Flavonoid biosynthesis [PATH:ko00941]; |
| Metabolism; Biosynthesis of Other Secondary Metabolites; Glucosinolate biosynthesis [PATH:ko00966]; |
| Metabolism; Biosynthesis of Other Secondary Metabolites; Isoquinoline alkaloid biosynthesis [PATH:ko00950]; |
| Metabolism; Biosynthesis of Other Secondary Metabolites; Novobiocin biosynthesis [PATH:ko00401]; |
| Metabolism; Biosynthesis of Other Secondary Metabolites; Phenylpropanoid biosynthesis [PATH:ko00940]; |
| Metabolism; Biosynthesis of Other Secondary Metabolites; Stilbenoid, diarylheptanoid and gingerol biosynthesis [PATH:ko00945]; |
| Metabolism; Biosynthesis of Other Secondary Metabolites; Tropane, piperidine and pyridine alkaloid biosynthesis [PATH:ko00960]; |
| Metabolism; Carbohydrate Metabolism; Amino sugar and nucleotide sugar metabolism [PATH:ko00520]; |
| Metabolism; Carbohydrate Metabolism; Ascorbate and aldarate metabolism [PATH:ko00053]; |
| Metabolism; Carbohydrate Metabolism; Butanoate metabolism [PATH:ko00650]; |
| Metabolism; Carbohydrate Metabolism; C5-Branched dibasic acid metabolism [PATH:ko00660]; |
| Metabolism; Carbohydrate Metabolism; Citrate cycle (TCA cycle) [PATH:ko00020]; |
| Metabolism; Carbohydrate Metabolism; Fructose and mannose metabolism [PATH:ko00051]; |
| Metabolism; Carbohydrate Metabolism; Galactose metabolism [PATH:ko00052]; |
| Metabolism; Carbohydrate Metabolism; Glycolysis / Gluconeogenesis [PATH:ko00010]; |
| Metabolism; Carbohydrate Metabolism; Glyoxylate and dicarboxylate metabolism [PATH:ko00630]; |
| Metabolism; Carbohydrate Metabolism; Inositol phosphate metabolism [PATH:ko00562]; |
| Metabolism; Carbohydrate Metabolism; Pentose and glucuronate interconversions [PATH:ko00040]; |
| Metabolism; Carbohydrate Metabolism; Pentose phosphate pathway [PATH:ko00030]; |
| Metabolism; Carbohydrate Metabolism; Propanoate metabolism [PATH:ko00640]; |
| Metabolism; Carbohydrate Metabolism; Pyruvate metabolism [PATH:ko00620]; |
| Metabolism; Carbohydrate Metabolism; Starch and sucrose metabolism [PATH:ko00500]; |
| Metabolism; Energy Metabolism; Carbon fixation in photosynthetic organisms [PATH:ko00710]; |
| Metabolism; Energy Metabolism; Carbon fixation pathways in prokaryotes [PATH:ko00720]; |
| Metabolism; Energy Metabolism; Methane metabolism [PATH:ko00680]; |
| Metabolism; Energy Metabolism; Nitrogen metabolism [PATH:ko00910]; |
| Metabolism; Energy Metabolism; Oxidative phosphorylation [PATH:ko00190]; |
| Metabolism; Energy Metabolism; Photosynthesis - antenna proteins [PATH:ko00196]; |
| Metabolism; Energy Metabolism; Photosynthesis [PATH:ko00195]; |
| Metabolism; Energy Metabolism; Photosynthesis proteins [BR:ko00194]; |
| Metabolism; Energy Metabolism; Sulfur metabolism [PATH:ko00920]; |
| Metabolism; Enzyme Families; Cytochrome P450 [BR:ko00199]; |
| Metabolism; Enzyme Families; Peptidases [BR:ko01002]; |
| Metabolism; Enzyme Families; Protein kinases [BR:ko01001]; |
| Metabolism; Glycan Biosynthesis and Metabolism; Glycosaminoglycan biosynthesis - chondroitin sulfate [PATH:ko00532]; |
| Metabolism; Glycan Biosynthesis and Metabolism; Glycosaminoglycan biosynthesis - heparan sulfate [PATH:ko00534]; |
| Metabolism; Glycan Biosynthesis and Metabolism; Glycosaminoglycan degradation [PATH:ko00531]; |
| Metabolism; Glycan Biosynthesis and Metabolism; Glycosphingolipid biosynthesis - ganglio series [PATH:ko00604]; |
| Metabolism; Glycan Biosynthesis and Metabolism; Glycosphingolipid biosynthesis - globo series [PATH:ko00603]; |
| Metabolism; Glycan Biosynthesis and Metabolism; Glycosylphosphatidylinositol(GPI)-anchor biosynthesis [PATH:ko00563]; |
| Metabolism; Glycan Biosynthesis and Metabolism; Glycosyltransferases [BR:ko01003]; |
| Metabolism; Glycan Biosynthesis and Metabolism; Lipopolysaccharide biosynthesis [PATH:ko00540]; |
| Metabolism; Glycan Biosynthesis and Metabolism; Lipopolysaccharide biosynthesis proteins [BR:ko01005]; |
| Metabolism; Glycan Biosynthesis and Metabolism; N-Glycan biosynthesis [PATH:ko00510]; |
| Metabolism; Glycan Biosynthesis and Metabolism; Other glycan degradation [PATH:ko00511]; |
| Metabolism; Glycan Biosynthesis and Metabolism; Other types of O-glycan biosynthesis [PATH:ko00514]; |
| Metabolism; Glycan Biosynthesis and Metabolism; Peptidoglycan biosynthesis [PATH:ko00550]; |
| Metabolism; Glycan Biosynthesis and Metabolism; Proteoglycans [BR:ko00535]; |
| Metabolism; Glycan Biosynthesis and Metabolism; Various types of N-glycan biosynthesis [PATH:ko00513]; |
| Metabolism; Lipid Metabolism; Arachidonic acid metabolism [PATH:ko00590]; |
| Metabolism; Lipid Metabolism; Biosynthesis of unsaturated fatty acids [PATH:ko01040]; |
| Metabolism; Lipid Metabolism; Ether lipid metabolism [PATH:ko00565]; |
| Metabolism; Lipid Metabolism; Fatty acid biosynthesis [PATH:ko00061]; |
| Metabolism; Lipid Metabolism; Fatty acid elongation in mitochondria [PATH:ko00062]; |
| Metabolism; Lipid Metabolism; Fatty acid metabolism [PATH:ko00071]; |
| Metabolism; Lipid Metabolism; Glycerolipid metabolism [PATH:ko00561]; |
| Metabolism; Lipid Metabolism; Glycerophospholipid metabolism [PATH:ko00564]; |
| Metabolism; Lipid Metabolism; Linoleic acid metabolism [PATH:ko00591]; |
| Metabolism; Lipid Metabolism; Lipid biosynthesis proteins [BR:ko01004]; |
| Metabolism; Lipid Metabolism; Primary bile acid biosynthesis [PATH:ko00120]; |
| Metabolism; Lipid Metabolism; Sphingolipid metabolism [PATH:ko00600]; |
| Metabolism; Lipid Metabolism; Steroid biosynthesis [PATH:ko00100]; |
| Metabolism; Lipid Metabolism; Steroid hormone biosynthesis [PATH:ko00140]; |
| Metabolism; Lipid Metabolism; Synthesis and degradation of ketone bodies [PATH:ko00072]; |
| Metabolism; Lipid Metabolism; alpha-Linolenic acid metabolism [PATH:ko00592]; |
| Metabolism; Metabolism of Cofactors and Vitamins; Biotin metabolism [PATH:ko00780]; |
| Metabolism; Metabolism of Cofactors and Vitamins; Folate biosynthesis [PATH:ko00790]; |
| Metabolism; Metabolism of Cofactors and Vitamins; Lipoic acid metabolism [PATH:ko00785]; |
| Metabolism; Metabolism of Cofactors and Vitamins; Nicotinate and nicotinamide metabolism [PATH:ko00760]; |
| Metabolism; Metabolism of Cofactors and Vitamins; One carbon pool by folate [PATH:ko00670]; |
| Metabolism; Metabolism of Cofactors and Vitamins; Pantothenate and CoA biosynthesis [PATH:ko00770]; |
| Metabolism; Metabolism of Cofactors and Vitamins; Porphyrin and chlorophyll metabolism [PATH:ko00860]; |
| Metabolism; Metabolism of Cofactors and Vitamins; Retinol metabolism [PATH:ko00830]; |
| Metabolism; Metabolism of Cofactors and Vitamins; Riboflavin metabolism [PATH:ko00740]; |
| Metabolism; Metabolism of Cofactors and Vitamins; Thiamine metabolism [PATH:ko00730]; |
| Metabolism; Metabolism of Cofactors and Vitamins; Ubiquinone and other terpenoid-quinone biosynthesis [PATH:ko00130]; |
| Metabolism; Metabolism of Cofactors and Vitamins; Vitamin B6 metabolism [PATH:ko00750]; |
| Metabolism; Metabolism of Other Amino Acids; Cyanoamino acid metabolism [PATH:ko00460]; |
| Metabolism; Metabolism of Other Amino Acids; D-Glutamine and D-glutamate metabolism [PATH:ko00471]; |
| Metabolism; Metabolism of Other Amino Acids; Glutathione metabolism [PATH:ko00480]; |
| Metabolism; Metabolism of Other Amino Acids; Phosphonate and phosphinate metabolism [PATH:ko00440]; |
| Metabolism; Metabolism of Other Amino Acids; Selenocompound metabolism [PATH:ko00450]; |
| Metabolism; Metabolism of Other Amino Acids; Taurine and hypotaurine metabolism [PATH:ko00430]; |
| Metabolism; Metabolism of Other Amino Acids; beta-Alanine metabolism [PATH:ko00410]; |
| Metabolism; Metabolism of Terpenoids and Polyketides; Biosynthesis of siderophore group nonribosomal peptides [PATH:ko01053]; |
| Metabolism; Metabolism of Terpenoids and Polyketides; Brassinosteroid biosynthesis [PATH:ko00905]; |
| Metabolism; Metabolism of Terpenoids and Polyketides; Carotenoid biosynthesis [PATH:ko00906]; |
| Metabolism; Metabolism of Terpenoids and Polyketides; Diterpenoid biosynthesis [PATH:ko00904]; |
| Metabolism; Metabolism of Terpenoids and Polyketides; Geraniol degradation [PATH:ko00281]; |
| Metabolism; Metabolism of Terpenoids and Polyketides; Limonene and pinene degradation [PATH:ko00903]; |
| Metabolism; Metabolism of Terpenoids and Polyketides; Polyketide sugar unit biosynthesis [PATH:ko00523]; |
| Metabolism; Metabolism of Terpenoids and Polyketides; Prenyltransferases [BR:ko01006]; |
| Metabolism; Metabolism of Terpenoids and Polyketides; Terpenoid backbone biosynthesis [PATH:ko00900]; |
| Metabolism; Metabolism of Terpenoids and Polyketides; Tetracycline biosynthesis [PATH:ko00253]; |
| Metabolism; Metabolism of Terpenoids and Polyketides; Zeatin biosynthesis [PATH:ko00908]; |
| Metabolism; Nucleotide Metabolism; Purine metabolism [PATH:ko00230]; |
| Metabolism; Nucleotide Metabolism; Pyrimidine metabolism [PATH:ko00240]; |
| Metabolism; Xenobiotics Biodegradation and Metabolism; Aminobenzoate degradation [PATH:ko00627]; |
| Metabolism; Xenobiotics Biodegradation and Metabolism; Benzoate degradation [PATH:ko00362]; |
| Metabolism; Xenobiotics Biodegradation; Bisphenol degradation [PATH:ko00363]; |
| Metabolism; Xenobiotics Biodegradation and Metabolism; Caprolactam degradation [PATH:ko00930]; |
| Metabolism; Xenobiotics Biodegradation and Metabolism; Chloroalkane and chloroalkene degradation [PATH:ko00625]; |
| Metabolism; Xenobiotics Biodegradation and Metabolism; Chlorocyclohexane and chlorobenzene degradation [PATH:ko00361]; |
| Metabolism; Xenobiotics Biodegradation and Metabolism; Dioxin degradation [PATH:ko00621]; |
| Metabolism; Xenobiotics Biodegradation and Metabolism; Drug metabolism - cytochrome P450 [PATH:ko00982]; |
| Metabolism; Xenobiotics Biodegradation and Metabolism; Drug metabolism - other enzymes [PATH:ko00983]; |
| Metabolism; Xenobiotics Biodegradation and Metabolism; Fluorobenzoate degradation [PATH:ko00364]; |
| Metabolism; Xenobiotics Biodegradation and Metabolism; Metabolism of xenobiotics by cytochrome P450 [PATH:ko00980]; |
| Metabolism; Xenobiotics Biodegradation and Metabolism; Naphthalene degradation [PATH:ko00626]; |
| Metabolism; Xenobiotics Biodegradation and Metabolism; Polycyclic aromatic hydrocarbon degradation [PATH:ko00624]; |
| Metabolism; Xenobiotics Biodegradation and Metabolism; Styrene degradation [PATH:ko00643]; |
| Metabolism; Xenobiotics Biodegradation and Metabolism; Toluene degradation [PATH:ko00623]; |
| >2. Genetic Information Processing |
| Genetic Information Processing; Folding, Sorting and Degradation; Chaperones and folding catalysts [BR:ko03110]; |
| Genetic Information Processing; Folding, Sorting and Degradation; Proteasome [BR:ko03051]; |
| Genetic Information Processing; Folding, Sorting and Degradation; Proteasome [PATH:ko03050]; |
| Genetic Information Processing; Folding, Sorting and Degradation; Protein export [PATH:ko03060]; |
| Genetic Information Processing; Folding, Sorting and Degradation; Protein processing in endoplasmic reticulum [PATH:ko04141]; |
| Genetic Information Processing; Folding, Sorting and Degradation; RNA degradation [PATH:ko03018]; |
| Genetic Information Processing; Folding, Sorting and Degradation; SNARE interactions in vesicular transport [PATH:ko04130]; |
| Genetic Information Processing; Folding, Sorting and Degradation; SNAREs [BR:ko04131]; |
| Genetic Information Processing; Folding, Sorting and Degradation; Sulfur relay system [PATH:ko04122]; |
| Genetic Information Processing; Folding, Sorting and Degradation; Ubiquitin mediated proteolysis [PATH:ko04120]; |
| Genetic Information Processing; Replication and Repair; Base excision repair [PATH:ko03410]; |
| Genetic Information Processing; Replication and Repair; Chromosome [BR:ko03036]; |
| Genetic Information Processing; Replication and Repair; DNA repair and recombination proteins [BR:ko03400]; |
| Genetic Information Processing; Replication and Repair; DNA replication [PATH:ko03030]; |
| Genetic Information Processing; Replication and Repair; DNA replication proteins [BR:ko03032]; |
| Genetic Information Processing; Replication and Repair; Homologous recombination [PATH:ko03440]; |
| Genetic Information Processing; Replication and Repair; Mismatch repair [PATH:ko03430]; |
| Genetic Information Processing; Replication and Repair; Non-homologous end-joining [PATH:ko03450]; |
| Genetic Information Processing; Replication and Repair; Nucleotide excision repair [PATH:ko03420]; |
| Genetic Information Processing; Transcription; Basal transcription factors [PATH:ko03022]; |
| Genetic Information Processing; Transcription; RNA polymerase [PATH:ko03020]; |
| Genetic Information Processing; Transcription; Spliceosome [BR:ko03041]; |
| Genetic Information Processing; Transcription; Spliceosome [PATH:ko03040]; |
| Genetic Information Processing; Transcription; Transcription factors [BR:ko03000]; |
| Genetic Information Processing; Translation; Aminoacyl-tRNA biosynthesis [PATH:ko00970]; |
| Genetic Information Processing; Translation; RNA transport [PATH:ko03013]; |
| Genetic Information Processing; Translation; Ribosome Biogenesis [BR:ko03009]; |
| Genetic Information Processing; Translation; Ribosome [BR:ko03011]; |
| Genetic Information Processing; Translation; Ribosome [PATH:ko03010]; |
| Genetic Information Processing; Translation; Ribosome biogenesis in eukaryotes [PATH:ko03008]; |
| Genetic Information Processing; Translation; Translation factors [BR:ko03012]; |
| Genetic Information Processing; Translation; mRNA surveillance pathway [PATH:ko03015]; |
| >3. Environmental Information Processing |
| Environmental Information Processing; Membrane Transport; ABC transporters [PATH:ko02010]; |
| Environmental Information Processing; Membrane Transport; Bacterial secretion system [PATH:ko03070]; |
| Environmental Information Processing; Membrane Transport; Secretion system [BR:ko02044]; |
| Environmental Information Processing; Membrane Transport; Transporters [BR:ko02000]; |
| Environmental Information Processing; Signal Transduction; Calcium signaling pathway [PATH:ko04020]; |
| Environmental Information Processing; Signal Transduction; ErbB signaling pathway [PATH:ko04012]; |
| Environmental Information Processing; Signal Transduction; Hedgehog signaling pathway [PATH:ko04340]; |
| Environmental Information Processing; Signal Transduction; Jak-STAT signaling |
| Environmental Information Processing; Signal Transduction; MAPK signaling pathway - fly [PATH:ko04013]; |
| Environmental Information Processing; Signal Transduction; MAPK signaling pathway - yeast [PATH:ko04011]; |
| Environmental Information Processing; Signal Transduction; MAPK signaling pathway [PATH:ko04010]; |
| Environmental Information Processing; Signal Transduction; Notch signaling pathway [PATH:ko04330]; |
| Environmental Information Processing; Signal Transduction; Phosphatidylinositol signaling system [PATH:ko04070]; |
| Environmental Information Processing; Signal Transduction; Plant hormone signal transduction [PATH:ko04075]; |
| Environmental Information Processing; Signal Transduction; TGF-beta signaling pathway [PATH:ko04350]; |
| Environmental Information Processing; Signal Transduction; Two-component system [PATH:ko02020]; |
| Environmental Information Processing; Signal Transduction; VEGF signaling pathway [PATH:ko04370]; |
| Environmental Information Processing; Signal Transduction; Wnt signaling pathway [PATH:ko04310]; |
| Environmental Information Processing; Signal Transduction; mTOR signaling pathway [PATH:ko04150]; |
| Environmental Information Processing; Signaling Molecules and Interaction; Bacterial toxins [BR:ko02042]; |
| Environmental Information Processing; Signaling Molecules and Interaction; Cellular antigens [BR:ko04090]; |
| Environmental Information Processing; Signaling Molecules and Interaction; G protein-coupled receptors [BR:ko04030]; |
| Environmental Information Processing; Signaling Molecules and Interaction; GTP-binding proteins [BR:ko04031]; |
| Environmental Information Processing; Signaling Molecules and Interaction; Glycan bindng proteins [BR:ko04091]; |
| Environmental Information Processing; Signaling Molecules and Interaction; Ion channels [BR:ko04040]; |
| Environmental Information Processing; Signaling Molecules and Interaction; Neuroactive ligand-receptor interaction [PATH:ko04080]; |
| >4. Cellular Processes |
| Cellular Processes; Cell Communication; Adherens junction [PATH:ko04520]; |
| Cellular Processes; Cell Communication; Focal adhesion [PATH:ko04510]; |
| Cellular Processes; Cell Communication; Gap junction [PATH:ko04540]; |
| Cellular Processes; Cell Communication; Tight junction [PATH:ko04530]; |
| Cellular Processes; Cell Growth and Death; Apoptosis [PATH:ko04210]; |
| Cellular Processes; Cell Growth and Death; Cell cycle - Caulobacter [PATH:ko04112]; |
| Cellular Processes; Cell Growth and Death; Cell cycle - yeast [PATH:ko04111]; |
| Cellular Processes; Cell Growth and Death; Cell cycle [PATH:ko04110]; |
| Cellular Processes; Cell Growth and Death; Meiosis - yeast [PATH:ko04113]; |
| Cellular Processes; Cell Growth and Death; Oocyte meiosis [PATH:ko04114]; |
| Cellular Processes; Cell Growth and Death; p53 signaling pathway [PATH:ko04115]; |
| Cellular Processes; Cell Motility; Cytoskeleton proteins [BR:ko04812]; |
| Cellular Processes; Cell Motility; Regulation of actin cytoskeleton [PATH:ko04810]; |
| Cellular Processes; Transport and Catabolism; Endocytosis [PATH:ko04144]; |
| Cellular Processes; Transport and Catabolism; Lysosome [PATH:ko04142]; |
| Cellular Processes; Transport and Catabolism; Peroxisome [PATH:ko04146]; |
| Cellular Processes; Transport and Catabolism; Phagosome [PATH:ko04145]; |
| Cellular Processes; Transport and Catabolism; Regulation of autophagy [PATH:ko04140]; |
| >5. Human Diseases |
| Human Diseases; Cancers; Acute myeloid leukemia [PATH:ko05221]; |
| Human Diseases; Cancers; Basal cell carcinoma [PATH:ko05217]; |
| Human Diseases; Cancers; Bladder cancer [PATH:ko05219]; |
| Human Diseases; Cancers; Chronic myeloid leukemia [PATH:ko05220]; |
| Human Diseases; Cancers; Colorectal cancer [PATH:ko05210]; |
| Human Diseases; Cancers; Endometrial cancer [PATH:ko05213]; |
| Human Diseases; Cancers; Glioma [PATH:ko05214]; |
| Human Diseases; Cancers; Melanoma [PATH:ko05218]; |
| Human Diseases; Cancers; Non-small cell lung cancer [PATH:ko05223]; |
| Human Diseases; Cancers; Pancreatic cancer [PATH:ko05212]; |
| Human Diseases; Cancers; Pathways in cancer [PATH:ko05200]; |
| Human Diseases; Cancers; Prostate cancer [PATH:ko05215]; |
| Human Diseases; Cancers; Renal cell carcinoma [PATH:ko05211]; |
| Human Diseases; Cancers; Small cell lung cancer [PATH:ko05222]; |
| Human Diseases; Cancers; Thyroid cancer [PATH:ko05216]; |
| Human Diseases; Cardiovascular Diseases; Arrhythmogenic right ventricular cardiomyopathy (ARVC) [PATH:ko05412]; |
| Human Diseases; Cardiovascular Diseases; Dilated cardiomyopathy (DCM) [PATH:ko05414]; |
| Human Diseases; Cardiovascular Diseases; Hypertrophic cardiomyopathy (HCM) [PATH:ko05410]; |
| Human Diseases; Cardiovascular Diseases; Viral myocarditis [PATH:ko05416]; |
| Human Diseases; Immune System Diseases; Primary immunodeficiency [PATH:ko05340]; |
| Human Diseases; Immune System Diseases; Rheumatoid arthritis [PATH:ko05323]; |
| Human Diseases; Immune System Diseases; Systemic lupus erythematosus [PATH:ko05322]; |
| Human Diseases; Infectious Diseases; African trypanosomiasis [PATH:ko05143]; |
| Human Diseases; Infectious Diseases; Amoebiasis [PATH:ko05146]; |
| Human Diseases; Infectious Diseases; Bacterial invasion of epithelial cells [PATH:ko05100]; |
| Human Diseases; Infectious Diseases; Chagas disease (American trypanosomiasis) [PATH:ko05142]; |
| Human Diseases; Infectious Diseases; Epithelial cell signaling in Helicobacter pylori infection [PATH:ko05120]; |
| Human Diseases; Infectious Diseases; Hepatitis C [PATH:ko05160]; |
| Human Diseases; Infectious Diseases; Leishmaniasis [PATH:ko05140]; |
| Human Diseases; Infectious Diseases; Measles [PATH:ko05162]; |
| Human Diseases; Infectious Diseases; Pathogenic Escherichia coli infection [PATH:ko05130]; |
| Human Diseases; Infectious Diseases; Shigellosis [PATH:ko05131]; |
| Human Diseases; Infectious Diseases; Toxoplasmosis [PATH:ko05145]; |
| Human Diseases; Infectious Diseases; Tuberculosis [PATH:ko05152]; |
| Human Diseases; Infectious Diseases; Vibrio cholerae infection [PATH:ko05110]; |
| Human Diseases; Infectious Diseases; Vibrio cholerae pathogenic cycle [PATH:ko05111]; |
| Human Diseases; Metabolic Diseases; Type I diabetes mellitus [PATH:ko04940]; |
| Human Diseases; Metabolic Diseases; Type II diabetes mellitus [PATH:ko04930]; |
| Human Diseases; Neurodegenerative Diseases; Alzheimer's disease [PATH:ko05010]; |
| Human Diseases; Neurodegenerative Diseases; Amyotrophic lateral sclerosis (ALS) [PATH:ko05014]; |
| Human Diseases; Neurodegenerative Diseases; Huntington's disease [PATH:ko05016]; |
| Human Diseases; Neurodegenerative Diseases; Parkinson's disease [PATH:ko05012]; |
| Human Diseases; Neurodegenerative Diseases; Prion diseases [PATH:ko05020]; |
