# Supplementary material for: Identifying the candidate genes involved in the calyx abscission process of 'Kuerlexiangli’ (Pyrus sinkiangensis Yu) by digital transcript abundance measurements
Source: BMC Genomics. 2013 Oct 23;14(1):727. doi: 10.1186/1471-2164-14-727 (PMC4046677; doi:10.1186/1471-2164-14-727)
Supplement: Supplementary file 3 — Additional file 3: List of significantly altered pathways with enriched differentially expressed genes after Flusilazole treatment and GA 3 treatment. The table provides the list of significantly alter biochemical pathways during calyx abscission process: Pathway ID, pathway name, the total number of genes with pathway annotation, the number of differentially expressed genes with pathway annotation of each comparison (Fifteen pairs comparison in total in this table, e.g. C1 vs C7 indicates the difference expressed genes in the comparison between C1and C7). (DOC 44 KB) [file 12864_2013_5444_MOESM3_ESM.doc]

**Additional file 3: List of significantly altered pathways with enriched differentially expressed genes after** **Flusilazole treatment and GA_3_ treatment.**

| Pathway ID | Pathway name | All genes with pathway annotation | DEGs with pathway annotation | | | | | | | | | | | | | |
| --- | --- | --- | --- | --- | --- | --- | --- | --- | --- | --- | --- | --- | --- | --- | --- | --- |
|  |  |  | C1  VS  C7 | C2  VS  C7 | C3  VS  C7 | C4  VS  C7 | C5  VS  C7 | C6  VS  C7 | C1  VS  C3 | C1  VS  C5 | C2  VS  C4 | C2  VS  C6 | C3  VS  C4 | C3  VS  C5 | C4  VS  C6 | C5  VS  C6 |
| ko00944 | Metabolism; Biosynthesis of Other Secondary Metabolites; Flavone and flavonol biosynthesis | 11 | 8 | 8 | 0 | 8 | 9 | 0 | 9 | 1 | 9 | 9 | 8 | 9 | 8 | 9 |
| ko00500 | Metabolism; Carbohydrate Metabolism; Starch and sucrose metabolism | 200 | 61 | 57 | 20 | 65 | 71 | 17 | 61 | 66 | 47 | 47 | 53 | 60 | 65 | 70 |
| ko00710 | Metabolism; Carbon fixation in photosynthetic organisms | 134 | 23 | 36 | 27 | 14 | 32 | 16 | 19 | 12 | 35 | 35 | 26 | 31 | 19 | 25 |
| ko00190 | Metabolism; Energy Metabolism; Oxidative phosphorylation | 284 | 82 | 78 | 60 | 18 | 66 | 52 | 34 | 40 | 43 | 100 | 53 | 30 | 64 | 105 |
| ko00194 | Metabolism; Energy Metabolism; Photosynthesis proteins | 261 | 183 | 225 | 137 | 37 | 150 | 78 | 41 | 81 | 170 | 228 | 122 | 25 | 131 | 176 |
| ko00195 | Metabolism; Energy Metabolism; Photosynthesis; | 230 | 174 | 196 | 129 | 26 | 137 | 72 | 39 | 76 | 156 | 208 | 122 | 19 | 131 | 175 |
| ko00908 | Metabolism; Metabolism of Terpenoids and Polyketides; Zeatin biosynthesis | 26 | 6 | 5 | 5 | 6 | 2 | 2 | 2 | 6 | 5 | 6 | 3 | 8 | 7 | 5 |
| ko03000 | Genetic Information Processing; Transcription  factors | 364 | 89 | 78 | 29 | 57 | 79 | 15 | 84 | 71 | 59 | 80 | 54 | 86 | 70 | 84 |
| ko04075 | Signal Transduction; Plant hormone signal transduction | 325 | 79 | 60 | 29 | 64 | 86 | 17 | 47 | 67 | 42 | 67 | 48 | 72 | 81 | 83 |
