# Supplementary material for: Identifying the candidate genes involved in the calyx abscission process of 'Kuerlexiangli’ (Pyrus sinkiangensis Yu) by digital transcript abundance measurements
Source: BMC Genomics. 2013 Oct 23;14(1):727. doi: 10.1186/1471-2164-14-727 (PMC4046677; doi:10.1186/1471-2164-14-727)
Supplement: Supplementary file 4 — Additional file 4: Categorization of significant genes encoding enzymes and proteins with a variety of biological functions. In this table, five functional categories of genes showed differential expression patterns after Flusilazole treatment and GA3 treatment. The details of gene expression analysis of 15 pairs of comparisons: Gene ID, gene description and TPM (transcripts copies per million tags) of genes. (DOC 2 MB) [file 12864_2013_5444_MOESM4_ESM.doc]

Additional file 4: Categorization of significant genes encoding enzymes and proteins with a variety of biological functions.

| Photosynthesis | | | | | | | | |
| --- | --- | --- | --- | --- | --- | --- | --- | --- |
| Gene ID | Gene Description | TPM (transcripts per million clean tag) | | | | | | |
|  |  | C1 | C2 | C3 | C4 | C5 | C6 | C7 |
| Pbr000144.1 | F-type H+-transporting ATPase | 27.057 | 75.332 | 29.285 | 58.042 | 51.595 | 30.688 | 24.313 |
| Pbr000210.1 | photosystem II | 272.921 | 489.79 | 291.158 | 196.686 | 259.076 | 240.287 | 200.959 |
| Pbr000879.2 | light-harvesting complex II chlorophyll | 41.266 | 193.004 | 55.05 | 79.349 | 73.059 | 60.072 | 42.42 |
| Pbr001154.1 | Ferredoxin-3, chloroplast precursor | 239.686 | 127.289 | 85.461 | 97.861 | 87.78 | 55.332 | 98.643 |
| Pbr001687.1 | chlorophyll A/B binding protein | 780.585 | 1777.776 | 702.129 | 884.314 | 876.703 | 476.427 | 312.519 |
| Pbr001992.4 | petA; apocytochrome f precursor; K02634 apocytochrome | 2.347 | 1.469 | 2.816 | 8.581 | 3.44 | 19.905 | 10.384 |
| Pbr001993.1 | atpB; ATP synthase CF1 beta subunit; K02112 F-type H+-transporting ATPase subunit beta [EC:3.6.3.14] | 0.371 | 1.069 | 1.549 | 3.375 | 1.926 | 9.36 | 3.419 |
| Pbr001996.1 | atpE; ATP synthase epsilon chain; K02114 F-type H+-transporting ATPase subunit epsilon [EC:3.6.3.14] | 0.494 | 0.935 | 1.83 | 2.603 | 1.376 | 14.455 | 5.065 |
| Pbr001997.1 | psaA; photosystem I P700 chlorophyll a apoprotein A1; K02689 photosystem I P700 chlorophyll a apoprotein A1 | 0 | 0.401 | 0 | 0.675 | 0.275 | 3.436 | 1.646 |
| Pbr001998.1 | psaB; photosystem I P700 chlorophyll a apoprotein A2; K02690 photosystem I P700 chlorophyll a apoprotein A2 | 1.112 | 0.534 | 1.267 | 2.7 | 0.688 | 6.635 | 3.925 |
| Pbr001999.2 | psbC; photosystem II 44 kDa protein; K02705 photosystem II CP43 chlorophyll apoprotein | 1.483 | 1.736 | 5.069 | 12.534 | 4.54 | 30.451 | 20.261 |
| Pbr002000.1 | hypothetical protein LOC100243760; K02116 ATP synthase protein I | 6.795 | 5.209 | 8.448 | 23.429 | 7.43 | 58.65 | 26.339 |
| Pbr002203.1 | photosystem II reaction center psbP protein; K02717 photosystem II oxygen-evolving enhancer protein 2 | 23.721 | 31.789 | 18.725 | 24.104 | 14.997 | 12.559 | 13.169 |
| Pbr002360.1 | hypothetical protein LOC100250504; K08912 light-harvesting complex II chlorophyll a/b binding protein 1 | 184.089 | 459.203 | 213.159 | 189.551 | 146.943 | 189.931 | 202.225 |
| Pbr002394.1 | Lhcb1-1; light-harvesting complex II protein Lhcb1; K08912 light-harvesting complex II chlorophyll a/b binding protein 1 | 9.637 | 24.176 | 13.094 | 14.077 | 15.41 | 11.256 | 7.598 |
| Pbr002395.1 | hypothetical protein LOC100250504; K08912 light-harvesting complex II chlorophyll a/b binding protein 1 | 7.537 | 6.678 | 5.069 | 7.038 | 2.614 | 3.91 | 33.936 |
| Pbr002396.1 | Photosystem II 22 kDa protein, chloroplast precursor, putative; K03542 photosystem II 22kDa protein | 1.977 | 5.877 | 2.112 | 3.471 | 3.164 | 2.251 | 1.899 |
| Pbr002834.1 | Lhcb6-1; light-harvesting complex II protein Lhcb6; K08917 light-harvesting complex II chlorophyll a/b binding protein 6 | 117.248 | 231.605 | 98.273 | 107.02 | 132.909 | 111.257 | 77.876 |
| Pbr004280.1 | chlorophyll A/B binding protein, putative; K08913 light-harvesting complex II chlorophyll a/b binding protein 2 | 24.463 | 106.319 | 25.483 | 47.918 | 52.283 | 31.161 | 25.326 |
| Pbr005261.1 | Plastocyanin A, chloroplast precursor, putative; K02638 plastocyanin | 701.761 | 1552.448 | 574.431 | 877.951 | 1152.015 | 720.15 | 309.733 |
| Pbr005785.1 | psbB; photosystem II P680 chlorophyll A apoprotein; K02704 photosystem II CP47 chlorophyll apoprotein | 168.027 | 389.748 | 178.243 | 213.173 | 261.002 | 206.045 | 192.602 |
| Pbr006588.2 | petA; apocytochrome f precursor; K02634 apocytochrome | 1.853 | 1.469 | 2.957 | 9.063 | 2.889 | 19.787 | 11.397 |
| Pbr006589.1 | petA; apocytochrome f precursor; K02634 apocytochrome | 0.371 | 1.069 | 1.549 | 3.375 | 1.926 | 9.36 | 3.419 |
| Pbr006592.1 | atpB; ATP synthase CF1 beta subunit; K02112 F-type H+-transporting ATPase subunit beta [EC:3.6.3.14] | 0.494 | 0.935 | 1.83 | 2.603 | 1.376 | 14.455 | 5.065 |
| Pbr006593.1 | atpE; ATP synthase epsilon chain; K02114 F-type H+-transporting ATPase subunit epsilon [EC:3.6.3.14] | 0 | 0.401 | 0 | 0.675 | 0.275 | 3.436 | 1.646 |
| Pbr006596.1 | psaA; photosystem I P700 chlorophyll a apoprotein A1; K02689 photosystem I P700 chlorophyll a apoprotein A1 | 2.1 | 1.736 | 3.097 | 12.63 | 4.816 | 31.28 | 16.082 |
| Pbr006597.1 | psbC; photosystem II 44 kDa protein; K02705 photosystem II CP43 chlorophyll apoprotein | 4.571 | 4.274 | 5.209 | 14.462 | 3.44 | 31.635 | 13.296 |
| Pbr006598.1 | psbD; photosystem II protein D2; K02706 photosystem II P680 reaction center D2 protein | 1.977 | 1.603 | 3.661 | 9.063 | 4.678 | 28.436 | 14.436 |
| Pbr006602.1 | atpH; ATPase III subunit; K02110 F-type H+-transporting ATPase subunit c [EC:3.6.3.14] | 0 | 0.134 | 0.704 | 1.928 | 1.101 | 6.398 | 2.279 |
| Pbr006603.1 | atpA; ATP synthase CF1 alpha subunit (EC:3.6.3.14); K02111 F-type H+-transporting ATPase subunit alpha [EC:3.6.3.14] | 2.718 | 2.271 | 6.758 | 14.366 | 8.393 | 25.83 | 11.65 |
| Pbr006604.1 | psbA; photosystem II protein D1; K02703 photosystem II P680 reaction center D1 protein | 24.339 | 10.952 | 15.769 | 140.38 | 9.906 | 91.826 | 68.253 |
| Pbr006618.1 | psbA; photosystem II protein D1; K02703 photosystem II P680 reaction center D1 protein | 24.339 | 10.952 | 15.769 | 140.38 | 9.906 | 91.826 | 68.253 |
| Pbr007291.1 | chlorophyll A/B binding protein, putative; K08910 light-harvesting complex I chlorophyll a/b binding protein 4 | 53.497 | 208.899 | 40.548 | 75.203 | 79.663 | 63.863 | 18.868 |
| Pbr008050.1 | hypothetical protein LOC100246457; K08916 light-harvesting complex II chlorophyll a/b binding protein 5 | 372.749 | 628.833 | 401.398 | 288.472 | 331.309 | 255.808 | 233.883 |
| Pbr008101.1 | Photosystem I reaction center subunit II, chloroplast precursor, putative; K02692 photosystem I subunit II | 107.488 | 272.61 | 116.153 | 144.043 | 159.876 | 132.229 | 116.371 |
| Pbr008607.1 | chlorophyll A/B binding protein, putative; K08912 light-harvesting complex II chlorophyll a/b binding protein 1 | 577.717 | 1341.947 | 462.22 | 659.861 | 675.551 | 338.511 | 224.132 |
| Pbr009064.1 | photosystem I reaction center subunit V (PsaG); K02721 photosystem II PsbW protein | 88.832 | 207.162 | 111.085 | 117.915 | 98.237 | 108.413 | 70.405 |
| Pbr009517.1 | Lhcb8; light-harvesting complex II protein Lhcb8; K08915 light-harvesting complex II chlorophyll a/b binding protein 4 | 43.489 | 84.147 | 32.523 | 25.839 | 27.242 | 39.218 | 26.845 |
| Pbr009594.1 | psaA; photosystem I P700 chlorophyll a apoprotein A1; K02689 photosystem I P700 chlorophyll a apoprotein A1 | 2.1 | 1.736 | 3.097 | 12.63 | 4.816 | 31.28 | 16.082 |
| Pbr009595.1 | psaB; photosystem I P700 chlorophyll a apoprotein A2; K02690 photosystem I P700 chlorophyll a apoprotein A2 | 1.483 | 1.736 | 5.069 | 12.534 | 4.54 | 30.451 | 20.261 |
| Pbr009596.1 | psbC; photosystem II 44 kDa protein; K02705 photosystem II CP43 chlorophyll apoprotein | 4.448 | 4.274 | 5.069 | 13.884 | 3.164 | 29.74 | 12.536 |
| Pbr009597.1 | psbD; photosystem II protein D2; K02706 photosystem II P680 reaction center D2 protein | 1.977 | 1.603 | 3.661 | 9.063 | 4.678 | 28.436 | 14.436 |
| Pbr009601.1 | atpA; ATPase alpha subunit; K02111 F-type H+-transporting ATPase subunit alpha [EC:3.6.3.14] | 2.718 | 2.271 | 6.758 | 14.655 | 8.668 | 26.659 | 11.65 |
| Pbr009602.1 | psbA; photosystem II protein D1; K02703 photosystem II P680 reaction center D1 protein | 24.339 | 10.952 | 15.769 | 140.38 | 9.906 | 91.826 | 68.253 |
| Pbr009612.1 | psbA; photosystem II protein D1; K02703 photosystem II P680 reaction center D1 protein | 24.339 | 10.952 | 15.769 | 140.38 | 9.906 | 91.826 | 68.253 |
| Pbr009613.1 | atpA; ATPase alpha subunit; K02111 F-type H+-transporting ATPase subunit alpha [EC:3.6.3.14] | 2.718 | 2.271 | 6.758 | 14.655 | 8.668 | 26.659 | 11.65 |
| Pbr009616.1 | psbD; photosystem II protein D2; K02706 photosystem II P680 reaction center D2 protein | 1.977 | 1.603 | 3.661 | 9.063 | 4.678 | 28.436 | 14.436 |
| Pbr009617.1 | psbC; photosystem II 44 kDa protein; K02705 photosystem II CP43 chlorophyll apoprotein | 4.571 | 4.274 | 5.209 | 14.462 | 3.44 | 31.635 | 13.296 |
| Pbr009618.1 | psaB; photosystem I P700 chlorophyll a apoprotein A2; K02690 photosystem I P700 chlorophyll a apoprotein A2 | 1.483 | 1.736 | 5.069 | 12.534 | 4.54 | 30.451 | 20.261 |
| Pbr009619.1 | psaA; photosystem I P700 chlorophyll a apoprotein A1; K02689 photosystem I P700 chlorophyll a apoprotein A1 | 2.1 | 1.736 | 3.097 | 12.63 | 4.816 | 31.28 | 16.082 |
| Pbr009621.1 | atpE; ATP synthase epsilon chain; K02114 F-type H+-transporting ATPase subunit epsilon [EC:3.6.3.14] | 0 | 0.401 | 0 | 0.675 | 0.275 | 3.436 | 1.646 |
| Pbr009622.1 | atpB; ATP synthase CF1 beta subunit; K02112 F-type H+-transporting ATPase subunit beta [EC:3.6.3.14] | 0.494 | 0.935 | 1.83 | 2.603 | 1.376 | 14.455 | 5.065 |
| Pbr009625.1 | petA; apocytochrome f precursor; K02634 apocytochrome | 0.371 | 1.069 | 1.549 | 3.375 | 1.926 | 9.36 | 3.419 |
| Pbr009626.1 | psbB; photosystem II P680 chlorophyll A apoprotein; K02704 photosystem II CP47 chlorophyll apoprotein | 0.494 | 0.534 | 1.408 | 4.049 | 1.651 | 8.057 | 5.065 |
| Pbr010005.1 | psbB; photosystem II P680 chlorophyll A apoprotein; | 1.853 | 1.469 | 2.957 | 9.063 | 2.889 | 19.787 | 11.397 |
| Pbr010006.1 | petA; apocytochrome f precursor; K02634 apocytochrome | 0.371 | 1.069 | 1.549 | 3.375 | 1.926 | 9.36 | 3.419 |
| Pbr010009.1 | atpB; ATP synthase CF1 beta subunit; K02112 F-type H+-transporting ATPase subunit beta [EC:3.6.3.14] | 0.494 | 0.935 | 1.83 | 2.603 | 1.376 | 14.455 | 5.065 |
| Pbr010010.1 | atpE; ATP synthase epsilon chain; K02114 F-type H+-transporting ATPase subunit epsilon [EC:3.6.3.14] | 0 | 0.401 | 0 | 0.675 | 0.275 | 3.436 | 1.646 |
| Pbr010012.1 | psaA; photosystem I P700 chlorophyll a apoprotein A1; K02689 photosystem I P700 chlorophyll a apoprotein A1 | 2.1 | 1.736 | 3.097 | 12.63 | 4.816 | 31.28 | 16.082 |
| Pbr010013.1 | psaB; photosystem I P700 chlorophyll a apoprotein A2; K02690 photosystem I P700 chlorophyll a apoprotein A2 | 1.483 | 1.736 | 5.069 | 12.534 | 4.54 | 30.451 | 20.261 |
| Pbr010014.1 | psbC; photosystem II 44 kDa protein; K02705 photosystem II CP43 chlorophyll apoprotein | 4.571 | 4.274 | 5.209 | 14.462 | 3.44 | 31.635 | 13.296 |
| Pbr010015.1 | psbD; photosystem II protein D2; K02706 photosystem II P680 reaction center D2 protein | 1.977 | 1.603 | 3.661 | 9.063 | 4.678 | 28.436 | 14.436 |
| Pbr010019.1 | atpH; ATPase III subunit; K02110 F-type H+-transporting ATPase subunit c [EC:3.6.3.14] | 0 | 0.134 | 0.704 | 1.928 | 1.101 | 6.398 | 2.279 |
| Pbr010020.1 | atpA; ATPase alpha subunit; K02111 F-type H+-transporting ATPase subunit alpha [EC:3.6.3.14] | 2.718 | 2.271 | 6.758 | 14.655 | 8.668 | 26.659 | 11.65 |
| Pbr010021.1 | psbA; photosystem II protein D1; K02703 photosystem II P680 reaction center D1 protein | 24.339 | 10.952 | 15.769 | 140.38 | 9.906 | 91.826 | 68.253 |
| Pbr010196.1 | psbA; photosystem II protein D1; K02703 photosystem II P680 reaction center D1 protein | 24.339 | 10.952 | 15.769 | 140.38 | 9.906 | 91.826 | 68.253 |
| Pbr010197.1 | atpA; ATPase alpha subunit; K02111 F-type H+-transporting ATPase subunit alpha [EC:3.6.3.14] | 2.718 | 2.271 | 6.758 | 14.655 | 8.668 | 26.659 | 11.65 |
| Pbr010455.1 | psaA; photosystem I P700 chlorophyll a apoprotein A1; K02689 photosystem I P700 chlorophyll a apoprotein A1 | 2.1 | 1.736 | 3.097 | 12.63 | 4.816 | 31.28 | 16.082 |
| Pbr010456.1 | psaB; photosystem I P700 chlorophyll a apoprotein A2; | 1.483 | 1.736 | 5.069 | 12.534 | 4.54 | 30.451 | 20.261 |
| Pbr010457.1 | psbC; photosystem II 44 kDa protein; K02705 photosystem II CP43 chlorophyll apoprotein | 4.571 | 4.274 | 5.209 | 14.462 | 3.44 | 31.635 | 13.296 |
| Pbr010458.1 | psbD; photosystem II protein D2; K02706 photosystem II P680 reaction center D2 protein | 1.977 | 1.603 | 3.661 | 9.063 | 4.678 | 28.436 | 14.436 |
| Pbr010462.1 | atpA; ATPase alpha subunit; K02111 F-type H+-transporting ATPase subunit alpha [EC:3.6.3.14] | 2.718 | 2.271 | 6.758 | 14.655 | 8.668 | 26.659 | 11.65 |
| Pbr010463.1 | psbA; photosystem II protein D1; K02703 photosystem II P680 reaction center D1 protein | 24.339 | 10.952 | 15.769 | 140.38 | 9.906 | 91.826 | 68.253 |
| Pbr010472.1 | psbA; photosystem II protein D1; K02703 photosystem II P680 reaction center D1 protein | 24.339 | 10.952 | 15.769 | 140.38 | 9.906 | 91.826 | 68.253 |
| Pbr010473.1 | atpA; ATPase alpha subunit; K02111 F-type H+-transporting ATPase subunit alpha [EC:3.6.3.14] | 2.718 | 2.271 | 6.758 | 14.655 | 8.668 | 26.659 | 11.65 |
| Pbr010476.1 | psbD; photosystem II protein D2; K02706 photosystem II P680 reaction center D2 protein | 1.977 | 1.603 | 3.661 | 9.063 | 4.678 | 28.436 | 14.436 |
| Pbr010477.1 | psbC; photosystem II 44 kDa protein; K02705 photosystem II CP43 chlorophyll apoprotein | 4.571 | 4.274 | 5.209 | 14.462 | 3.44 | 31.635 | 13.296 |
| Pbr010478.1 | psaB; photosystem I P700 chlorophyll a apoprotein A2; K02690 photosystem I P700 chlorophyll a apoprotein A2 | 1.483 | 1.736 | 5.069 | 12.534 | 4.54 | 30.451 | 20.261 |
| Pbr010479.1 | psaA; photosystem I P700 chlorophyll a apoprotein A1; K02689 photosystem I P700 chlorophyll a apoprotein A1 | 2.1 | 1.736 | 3.097 | 12.63 | 4.816 | 31.28 | 16.082 |
| Pbr010482.1 | atpE; ATP synthase epsilon chain; K02114 F-type H+-transporting ATPase subunit epsilon [EC:3.6.3.14] | 0 | 0.401 | 0 | 0.675 | 0.275 | 3.436 | 1.646 |
| Pbr010483.1 | atpB; ATP synthase CF1 beta subunit; K02112 F-type H+-transporting ATPase subunit beta [EC:3.6.3.14] | 0.494 | 0.935 | 1.83 | 2.603 | 1.376 | 14.455 | 5.065 |
| Pbr010895.1 | Lhcb8; light-harvesting complex II protein Lhcb8; K08915 light-harvesting complex II chlorophyll a/b binding protein 4 | 20.262 | 39.937 | 16.473 | 13.98 | 13.621 | 14.574 | 10.384 |
| Pbr011422.1 | chlorophyll A/B binding protein, putative; K08910 light-harvesting complex I chlorophyll a/b binding protein 4 | 68.57 | 283.963 | 43.927 | 71.732 | 103.465 | 70.38 | 20.767 |
| Pbr011841.1 | hypothetical protein LOC100244206; K02639 ferredoxin | 3.212 | 5.476 | 1.83 | 3.375 | 7.155 | 4.384 | 4.812 |
| Pbr012009.1 | psaA; photosystem I P700 chlorophyll a apoprotein A1; K02689 photosystem I P700 chlorophyll a apoprotein A1 | 0.618 | 0.668 | 0.422 | 5.206 | 1.926 | 9.716 | 5.065 |
| Pbr012449.2 | psbB; photosystem II P680 chlorophyll A apoprotein; K02704 photosystem II CP47 chlorophyll apoprotein | 1.853 | 1.469 | 2.957 | 9.063 | 2.889 | 19.787 | 11.397 |
| Pbr012450.1 | petA; apocytochrome f precursor; K02634 apocytochrome f | 0.865 | 2.271 | 2.393 | 4.435 | 2.752 | 10.308 | 4.305 |
| Pbr012798.1 | hypothetical protein LOC100246457; K08916 light-harvesting complex II chlorophyll a/b binding protein 5 | 141.093 | 315.352 | 153.886 | 150.021 | 164.967 | 143.959 | 81.675 |
| Pbr014580.1 | Photosystem I reaction center subunit III, chloroplast precursor, putative; K02694 photosystem I subunit III | 84.384 | 269.672 | 90.388 | 119.458 | 130.845 | 106.873 | 96.871 |
| Pbr015123.1 | hypothetical protein LOC100256544; K14172 light-harvesting complex II chlorophyll a/b binding protein 7 | 7.166 | 8.014 | 7.321 | 5.399 | 3.164 | 3.081 | 3.925 |
| Pbr015169.1 | hypothetical protein LOC100266036; K08908 light-harvesting complex I chlorophyll a/b binding protein 2 | 179.517 | 455.33 | 248.779 | 161.591 | 223.303 | 249.884 | 196.78 |
| Pbr017226.1 | photosystem I reaction center subunit N PsaN; K02701 photosystem I subunit PsaN | 63.257 | 175.641 | 80.392 | 63.537 | 83.378 | 80.807 | 63.061 |
| Pbr017917.2 | hypothetical protein; K14332 photosystem I subunit PsaO | 84.755 | 152.533 | 142.2 | 93.04 | 58.887 | 95.973 | 97.251 |
| Pbr018027.1 | Cytochrome b6-f complex iron-sulfur subunit, chloroplast precursor, putative (EC:1.10.99.1); K02636 cytochrome b6-f complex iron-sulfur subunit [EC:1.10.99.1] | 62.763 | 123.416 | 68.566 | 51.389 | 45.816 | 60.309 | 69.012 |
| Pbr019044.1 | psbB; photosystem II P680 chlorophyll A apoprotein; K02704 photosystem II CP47 chlorophyll apoprotein | 1.853 | 1.469 | 2.957 | 9.063 | 2.889 | 19.787 | 11.397 |
| Pbr019045.1 | psbE; cytochrome b559 alpha subunit; K02707 photosystem II cytochrome b559 subunit alpha | 0.124 | 0.267 | 0.422 | 0.964 | 0 | 2.251 | 1.52 |
| Pbr019046.1 | petA; apocytochrome f precursor; K02634 apocytochrome | 0.371 | 1.069 | 1.549 | 3.375 | 1.926 | 9.36 | 3.419 |
| Pbr019048.1 | atpB; ATP synthase CF1 beta subunit; K02112 F-type H+-transporting ATPase subunit beta [EC:3.6.3.14] | 0.494 | 0.935 | 1.83 | 2.603 | 1.376 | 14.455 | 5.065 |
| Pbr019052.1 | psaA; photosystem I P700 chlorophyll a apoprotein A1; K02689 photosystem I P700 chlorophyll a apoprotein A1 | 2.224 | 1.736 | 3.097 | 13.787 | 4.816 | 32.346 | 16.842 |
| Pbr019053.1 | psaB; photosystem I P700 chlorophyll a apoprotein A2; K02690 photosystem I P700 chlorophyll a apoprotein A2 | 1.483 | 1.736 | 5.069 | 12.148 | 4.403 | 29.858 | 19.754 |
| Pbr019054.2 | psbC; photosystem II 44 kDa protein; K02705 photosystem II CP43 chlorophyll apoprotein | 4.571 | 4.274 | 5.209 | 14.462 | 3.44 | 31.635 | 13.296 |
| Pbr019058.1 | atpA; ATPase alpha subunit; K02111 F-type H+-transporting ATPase subunit alpha [EC:3.6.3.14] | 2.718 | 2.271 | 6.758 | 14.655 | 8.668 | 26.659 | 11.65 |
| Pbr019059.1 | psbA; photosystem II protein D1; K02703 photosystem II P680 reaction center D1 protein | 24.339 | 10.952 | 15.769 | 140.38 | 9.906 | 91.826 | 68.253 |
| Pbr019069.1 | psbA; photosystem II protein D1; K02703 photosystem II P680 reaction center D1 protein | 24.339 | 10.952 | 15.769 | 140.38 | 9.906 | 91.826 | 68.253 |
| Pbr019070.1 | atpA; ATPase alpha subunit; K02111 F-type H+-transporting ATPase subunit alpha [EC:3.6.3.14] | 2.718 | 2.271 | 6.758 | 14.655 | 8.668 | 26.659 | 11.65 |
| Pbr019071.1 | atpH; ATPase III subunit; K02110 F-type H+-transporting ATPase subunit c [EC:3.6.3.14] | 0 | 0.134 | 0.704 | 1.928 | 1.101 | 6.398 | 2.279 |
| Pbr019098.1 | chlorophyll A/B binding protein, putative; K08912 light-harvesting complex II chlorophyll a/b binding protein 1 | 836.059 | 1872.341 | 744.367 | 939.946 | 938.48 | 509.01 | 330.12 |
| Pbr019168.1 | photosystem I reaction centre subunit VI (H) (PSAH); K02695 photosystem I subunit VI | 106.5 | 184.456 | 93.486 | 58.427 | 62.189 | 65.759 | 51.791 |
| Pbr019368.1 | photosystem II oxygen-evolving complex 33 KDa subunit; K02716 photosystem II oxygen-evolving enhancer protein 1 | 203.362 | 487.386 | 255.819 | 272.082 | 299.389 | 284.719 | 204.631 |
| Pbr020932.1 | hypothetical protein LOC100242061; K02639 ferredoxin | 24.71 | 32.323 | 25.483 | 22.657 | 18.849 | 13.863 | 12.41 |
| Pbr020992.1 | psaB; photosystem I P700 chlorophyll a apoprotein A2; K02690 photosystem I P700 chlorophyll a apoprotein A2 | 0.618 | 0.801 | 2.816 | 6.942 | 3.027 | 16.943 | 10.13 |
| Pbr020993.1 | psbC; photosystem II 44 kDa protein; K02705 photosystem II CP43 chlorophyll apoprotein | 4.571 | 4.274 | 5.209 | 14.462 | 3.44 | 31.635 | 13.296 |
| Pbr020994.1 | psbD; photosystem II protein D2; K02706 photosystem II P680 reaction center D2 protein | 1.977 | 1.603 | 3.661 | 9.063 | 4.678 | 28.436 | 14.436 |
| Pbr020997.1 | atpA; ATPase alpha subunit; K02111 F-type H+-transporting ATPase subunit alpha [EC:3.6.3.14] | 2.718 | 2.271 | 6.758 | 14.655 | 8.668 | 26.659 | 11.65 |
| Pbr020998.1 | psbA; photosystem II protein D1; K02703 photosystem II P680 reaction center D1 protein | 24.339 | 10.952 | 15.769 | 140.38 | 9.906 | 91.826 | 68.253 |
| Pbr021078.1 | hypothetical protein LOC100261762; K02698 photosystem I subunit X | 58.562 | 154.537 | 42.097 | 78.289 | 76.911 | 54.384 | 34.063 |
| Pbr021388.1 | Photosystem I reaction center subunit III, chloroplast precursor, putative; K02694 photosystem I subunit III | 62.516 | 198.48 | 62.089 | 80.024 | 93.697 | 76.541 | 61.921 |
| Pbr021654.1 | chlorophyll A/B binding protein, putative; K08915 light-harvesting complex II chlorophyll a/b binding protein 4 | 157.155 | 380.666 | 214.848 | 213.365 | 218.075 | 208.651 | 164.49 |
| Pbr021726.1 | hypothetical protein; K14332 photosystem I subunit PsaO | 53.62 | 95.5 | 52.234 | 31.72 | 41.689 | 33.057 | 28.618 |
| Pbr022044.2 | chlorophyll A/B binding protein, putative; K08907 light-harvesting complex I chlorophyll a/b binding protein 1 | 141.217 | 316.286 | 219.354 | 182.706 | 151.621 | 198.817 | 143.217 |
| Pbr022470.1 | hypothetical protein LOC100243760; K02116 ATP synthase protein I | 21.127 | 30.587 | 19.57 | 23.718 | 15.272 | 13.033 | 12.536 |
| Pbr022840.1 | photosystem II oxygen-evolving complex 33 KDa subunit; K02716 photosystem II oxygen-evolving enhancer protein 1 | 327.777 | 740.362 | 551.2 | 456.33 | 466.419 | 484.602 | 439.907 |
| Pbr023086.1 | hypothetical protein LOC100242061; K02639 ferredoxin | 19.521 | 26.046 | 18.162 | 18.319 | 13.621 | 9.953 | 9.877 |
| Pbr023131.1 | Photosystem I reaction center subunit V, chloroplast precursor, putative; K08905 photosystem I subunit V | 78.083 | 232.006 | 86.305 | 129.099 | 118.187 | 83.295 | 48.752 |
| Pbr023332.1 | hypothetical protein LOC100241976; K02641 ferredoxin--NADP+ reductase [EC:1.18.1.2] | 64.493 | 165.089 | 79.407 | 89.087 | 66.317 | 71.92 | 55.59 |
| Pbr023911.1 | chlorophyll A/B binding protein, putative; K08907 light-harvesting complex I chlorophyll a/b binding protein 1 | 57.203 | 130.495 | 79.266 | 80.892 | 75.673 | 68.84 | 47.866 |
| Pbr024832.1 | hypothetical protein LOC100260599; K08914 light-harvesting complex II chlorophyll a/b binding protein 3 | 32.617 | 132.365 | 27.595 | 51.582 | 87.092 | 49.171 | 14.309 |
| Pbr025296.1 | photosystem I reaction center subunit V (PsaG); K02721 photosystem II PsbW protein | 76.724 | 167.092 | 82.082 | 69.226 | 64.941 | 71.328 | 45.839 |
| Pbr025867.1 | hypothetical protein LOC100260011; K08903 photosystem II 13kDa protein | 6.177 | 20.436 | 3.097 | 10.316 | 12.933 | 6.872 | 4.812 |
| Pbr026445.1 | PSBP2; putative oxygen evolving enhancer protein; K02717 photosystem II oxygen-evolving enhancer protein 2 | 4.077 | 9.75 | 2.393 | 4.531 | 6.467 | 4.384 | 4.812 |
| Pbr027049.1 | psbD; photosystem II protein D2; K02706 photosystem II P680 reaction center D2 protein | 1.977 | 1.603 | 3.661 | 9.063 | 4.678 | 28.436 | 14.436 |
| Pbr027050.1 | psbC; photosystem II 44 kDa protein; K02705 photosystem II CP43 chlorophyll apoprotein | 4.571 | 4.274 | 5.209 | 14.462 | 3.44 | 31.635 | 13.296 |
| Pbr027051.1 | psaB; photosystem I P700 chlorophyll a apoprotein A2; K02690 photosystem I P700 chlorophyll a apoprotein A2 | 1.483 | 1.736 | 5.069 | 12.534 | 4.54 | 30.451 | 20.261 |
| Pbr027052.1 | psaA; photosystem I P700 chlorophyll a apoprotein A1; K02689 photosystem I P700 chlorophyll a apoprotein A1 | 2.1 | 1.736 | 3.097 | 12.63 | 4.816 | 31.28 | 16.082 |
| Pbr027054.1 | atpA; ATPase alpha subunit; K02111 F-type H+-transporting ATPase subunit alpha [EC:3.6.3.14] | 2.718 | 2.271 | 6.758 | 14.655 | 8.668 | 26.659 | 11.65 |
| Pbr027057.1 | psbD; photosystem II protein D2; K02706 photosystem II P680 reaction center D2 protein | 1.977 | 1.603 | 3.661 | 9.063 | 4.678 | 28.436 | 14.436 |
| Pbr027058.1 | psbC; photosystem II 44 kDa protein; K02705 photosystem II CP43 chlorophyll apoprotein | 4.571 | 4.274 | 5.209 | 14.462 | 3.44 | 31.635 | 13.296 |
| Pbr027059.1 | psaB; photosystem I P700 chlorophyll a apoprotein A2; K02690 photosystem I P700 chlorophyll a apoprotein A2 | 1.483 | 1.736 | 5.069 | 12.534 | 4.54 | 30.451 | 20.261 |
| Pbr027060.1 | psaA; photosystem I P700 chlorophyll a apoprotein A1; K02689 photosystem I P700 chlorophyll a apoprotein A1 | 2.1 | 1.736 | 3.097 | 12.63 | 4.816 | 31.28 | 16.082 |
| Pbr027062.1 | atpE; ATP synthase epsilon chain; K02114 F-type H+-transporting ATPase subunit epsilon [EC:3.6.3.14] | 0 | 0.401 | 0 | 0.675 | 0.275 | 3.436 | 1.646 |
| Pbr027063.1 | atpB; ATP synthase CF1 beta subunit; K02112 F-type H+-transporting ATPase subunit beta [EC:3.6.3.14] | 0.494 | 0.935 | 1.83 | 2.603 | 1.376 | 14.455 | 5.065 |
| Pbr027066.1 | petA; apocytochrome f precursor; K02634 apocytochrome | 0.371 | 1.069 | 1.549 | 3.375 | 1.926 | 9.36 | 3.419 |
| Pbr027067.1 | psbB; photosystem II P680 chlorophyll A apoprotein; K02704 photosystem II CP47 chlorophyll apoprotein | 1.853 | 1.469 | 2.957 | 9.063 | 2.889 | 19.787 | 11.397 |
| Pbr027080.1 | psbB; photosystem II P680 chlorophyll A apoprotein; K02704 photosystem II CP47 chlorophyll apoprotein | 1.853 | 1.469 | 2.957 | 9.063 | 2.889 | 19.787 | 11.397 |
| Pbr027081.1 | petA; apocytochrome f precursor; K02634 apocytochrome f | 0.371 | 1.069 | 1.549 | 3.375 | 1.926 | 9.36 | 3.419 |
| Pbr027084.1 | atpB; ATP synthase CF1 beta subunit; K02112 F-type H+-transporting ATPase subunit beta [EC:3.6.3.14] | 0.494 | 0.935 | 1.83 | 2.603 | 1.376 | 14.455 | 5.065 |
| Pbr027085.1 | atpE; ATP synthase epsilon chain; K02114 F-type H+-transporting ATPase subunit epsilon [EC:3.6.3.14] | 0 | 0.401 | 0 | 0.675 | 0.275 | 3.436 | 1.646 |
| Pbr027087.1 | psaA; photosystem I P700 chlorophyll a apoprotein A1; K02689 photosystem I P700 chlorophyll a apoprotein A1 | 2.1 | 1.736 | 3.097 | 12.63 | 4.816 | 31.28 | 16.082 |
| Pbr027088.1 | psaB; photosystem I P700 chlorophyll a apoprotein A2; K02690 photosystem I P700 chlorophyll a apoprotein A2 | 1.483 | 1.736 | 5.069 | 12.534 | 4.54 | 30.451 | 20.261 |
| Pbr027089.1 | psbC; photosystem II 44 kDa protein; K02705 photosystem II CP43 chlorophyll apoprotein | 4.571 | 4.274 | 5.209 | 14.462 | 3.44 | 31.635 | 13.296 |
| Pbr027090.1 | psbD; photosystem II protein D2; K02706 photosystem II P680 reaction center D2 protein | 1.977 | 1.603 | 3.661 | 9.063 | 4.678 | 28.436 | 14.436 |
| Pbr027094.1 | atpH; ATPase III subunit; K02110 F-type H+-transporting ATPase subunit c [EC:3.6.3.14] | 0 | 0.134 | 0.704 | 1.928 | 1.101 | 6.398 | 2.279 |
| Pbr027095.1 | atpA; ATPase alpha subunit; K02111 F-type H+-transporting ATPase subunit alpha [EC:3.6.3.14] | 2.718 | 2.271 | 6.758 | 14.655 | 8.668 | 26.659 | 11.65 |
| Pbr027732.1 | chlorophyll A/B binding protein, putative; K08908 light-harvesting complex I chlorophyll a/b binding protein 2 | 21.003 | 51.423 | 20.696 | 14.462 | 22.151 | 25.237 | 24.439 |
| Pbr027854.1 | hypothetical protein; K02109 F-type H+-transporting ATPase subunit b [EC:3.6.3.14] | 76.106 | 147.057 | 95.316 | 98.15 | 71.958 | 75.356 | 63.314 |
| Pbr028189.1 | PSBP2; putative oxygen evolving enhancer protein; K02717 photosystem II oxygen-evolving enhancer protein 2 | 3.83 | 9.216 | 2.816 | 4.628 | 8.393 | 2.844 | 4.052 |
| Pbr028277.1 | hypothetical protein LOC100264091; K02115 F-type H+-transporting ATPase subunit gamma [EC:3.6.3.14] | 8.772 | 6.411 | 2.957 | 5.496 | 7.017 | 1.896 | 3.419 |
| Pbr028626.1 | photosystem I reaction centre subunit VI (H) (PSAH); K02695 photosystem I subunit VI | 183.347 | 288.505 | 168.528 | 122.543 | 129.469 | 139.575 | 121.943 |
| Pbr028645.1 | photosystem I reaction centre subunit VI (H) (PSAH); K02695 photosystem I subunit VI | 183.347 | 288.505 | 168.528 | 122.543 | 129.469 | 139.575 | 121.943 |
| Pbr029189.1 | psbA; photosystem II protein D1; K02703 photosystem II P680 reaction center D1 protein | 24.339 | 10.952 | 15.769 | 140.38 | 9.906 | 91.826 | 68.253 |
| Pbr029190.1 | atpA; ATPase alpha subunit; K02111 F-type H+-transporting ATPase subunit alpha [EC:3.6.3.14] | 2.718 | 2.271 | 6.758 | 14.655 | 8.668 | 26.659 | 11.65 |
| Pbr029193.1 | psbD; photosystem II protein D2; K02706 photosystem II P680 reaction center D2 protein | 1.977 | 1.603 | 3.661 | 9.063 | 4.678 | 28.436 | 14.436 |
| Pbr029194.1 | psbC; photosystem II 44 kDa protein; K02705 photosystem II CP43 chlorophyll apoprotein | 4.571 | 4.274 | 5.209 | 14.462 | 3.44 | 31.635 | 13.296 |
| Pbr029195.1 | psaB; photosystem I P700 chlorophyll a apoprotein A2; K02690 photosystem I P700 chlorophyll a apoprotein A2 | 1.483 | 1.736 | 5.069 | 12.534 | 4.54 | 30.451 | 20.261 |
| Pbr029196.1 | psaA; photosystem I P700 chlorophyll a apoprotein A1; K02689 photosystem I P700 chlorophyll a apoprotein A1 | 2.1 | 1.736 | 3.097 | 12.63 | 4.816 | 31.28 | 16.082 |
| Pbr029198.1 | atpE; ATP synthase epsilon chain; K02114 F-type H+-transporting ATPase subunit epsilon [EC:3.6.3.14] | 0 | 0.401 | 0 | 0.675 | 0.275 | 3.436 | 1.646 |
| Pbr029199.1 | atpB; ATP synthase CF1 beta subunit; K02112 F-type H+-transporting ATPase subunit beta [EC:3.6.3.14] | 0.494 | 0.935 | 1.83 | 2.603 | 1.376 | 14.455 | 5.065 |
| Pbr029202.1 | petA; apocytochrome f precursor; K02634 apocytochrome f | 0.371 | 1.069 | 1.549 | 3.375 | 1.926 | 9.36 | 3.419 |
| Pbr029203.1 | psbB; photosystem II P680 chlorophyll A apoprotein; K02704 photosystem II CP47 chlorophyll apoprotein | 1.853 | 1.469 | 2.957 | 9.063 | 2.889 | 19.787 | 11.397 |
| Pbr029215.1 | psbB; photosystem II P680 chlorophyll A apoprotein; K02704 photosystem II CP47 chlorophyll apoprotein | 1.853 | 1.469 | 2.957 | 9.063 | 2.889 | 19.787 | 11.397 |
| Pbr029216.1 | petA; apocytochrome f precursor; K02634 apocytochrome f | 0.371 | 1.069 | 1.549 | 3.375 | 1.926 | 9.36 | 3.419 |
| Pbr029219.1 | atpB; ATP synthase CF1 beta subunit; K02112 F-type H+-transporting ATPase subunit beta [EC:3.6.3.14] | 0.494 | 0.935 | 1.83 | 2.603 | 1.376 | 14.455 | 5.065 |
| Pbr029220.1 | atpE; ATP synthase epsilon chain; K02114 F-type H+-transporting ATPase subunit epsilon [EC:3.6.3.14] | 0 | 0.401 | 0 | 0.675 | 0.275 | 3.436 | 1.646 |
| Pbr029222.1 | psaA; photosystem I P700 chlorophyll a apoprotein A1; K02689 photosystem I P700 chlorophyll a apoprotein A1 | 2.1 | 1.736 | 3.097 | 12.63 | 4.816 | 31.28 | 16.082 |
| Pbr029223.1 | psaB; photosystem I P700 chlorophyll a apoprotein A2; K02690 photosystem I P700 chlorophyll a apoprotein A2 | 1.483 | 1.736 | 5.069 | 12.534 | 4.54 | 30.451 | 20.261 |
| Pbr029224.1 | psbC; photosystem II 44 kDa protein; K02705 photosystem II CP43 chlorophyll apoprotein | 4.571 | 4.274 | 5.209 | 14.462 | 3.44 | 31.635 | 13.296 |
| Pbr029225.1 | psbD; photosystem II protein D2; K02706 photosystem II P680 reaction center D2 protein | 1.977 | 1.603 | 3.661 | 9.063 | 4.678 | 28.436 | 14.436 |
| Pbr029229.1 | atpH; ATPase III subunit; K02110 F-type H+-transporting ATPase subunit c [EC:3.6.3.14] | 0 | 0.134 | 0.704 | 1.928 | 1.101 | 6.398 | 2.279 |
| Pbr029230.1 | atpA; ATPase alpha subunit; K02111 F-type H+-transporting ATPase subunit alpha [EC:3.6.3.14] | 2.718 | 2.271 | 6.758 | 14.655 | 8.668 | 26.659 | 11.65 |
| Pbr029231.1 | psbA; photosystem II protein D1; K02703 photosystem II P680 reaction center D1 protein | 24.339 | 10.952 | 15.769 | 140.38 | 9.906 | 91.826 | 68.253 |
| Pbr029473.1 | hypothetical protein LOC100264091; K02115 F-type H+-transporting ATPase subunit gamma [EC:3.6.3.14] | 11.367 | 16.028 | 4.787 | 8.774 | 16.51 | 4.976 | 4.559 |
| Pbr029610.1 | Oxygen-evolving enhancer protein 2, chloroplast precursor, putative (EC:1.3.1.74); K02717 photosystem II oxygen-evolving enhancer protein 2 | 325.923 | 739.026 | 445.606 | 324.146 | 337.776 | 471.924 | 366.462 |
| Pbr029644.1 | hypothetical protein LOC100250504; K08912 light-harvesting complex II chlorophyll a/b binding protein 1 | 0.494 | 0.534 | 0.141 | 0.289 | 0.138 | 0.237 | 2.659 |
| Pbr029815.1 | psaA; photosystem I P700 chlorophyll a apoprotein A1; K02689 photosystem I P700 chlorophyll a apoprotein A1 | 0.371 | 0.267 | 0.141 | 1.35 | 0.826 | 3.318 | 2.279 |
| Pbr030142.1 | hypothetical protein; K02113 F-type H+-transporting ATPase subunit delta [EC:3.6.3.14] | 38.053 | 79.072 | 24.216 | 77.228 | 57.374 | 17.299 | 35.456 |
| Pbr030680.1 | Photosystem II 11 kDa protein precursor, putative; K08902 photosystem II Psb27 protein | 10.502 | 47.55 | 8.307 | 16.969 | 21.326 | 17.891 | 9.624 |
| Pbr030867.1 | hypothetical protein; K02113 F-type H+-transporting ATPase subunit delta [EC:3.6.3.14] | 39.659 | 85.616 | 25.765 | 80.313 | 59.713 | 18.839 | 38.622 |
| Pbr030966.1 | psaB; photosystem I P700 chlorophyll a apoprotein A2; K02690 photosystem I P700 chlorophyll a apoprotein A2 | 1.483 | 1.736 | 5.069 | 12.534 | 4.54 | 30.451 | 20.261 |
| Pbr030967.1 | psaA; photosystem I P700 chlorophyll a apoprotein A1; K02689 photosystem I P700 chlorophyll a apoprotein A1 | 2.1 | 1.736 | 3.097 | 12.63 | 4.816 | 31.28 | 16.082 |
| Pbr030969.1 | atpE; ATP synthase epsilon chain; K02114 F-type H+-transporting ATPase subunit epsilon [EC:3.6.3.14] | 0 | 0.401 | 0 | 0.675 | 0.275 | 3.436 | 1.646 |
| Pbr030970.1 | atpB; ATP synthase CF1 beta subunit | 0.494 | 0.801 | 1.83 | 2.507 | 1.376 | 14.337 | 4.939 |
| Pbr030972.1 | petA; apocytochrome f precursor; K02634 apocytochrome f | 0.371 | 1.069 | 1.549 | 3.375 | 1.926 | 9.36 | 3.419 |
| Pbr030973.1 | psbB; photosystem II P680 chlorophyll A apoprotein; K02704 photosystem II CP47 chlorophyll apoprotein | 1.853 | 1.469 | 2.957 | 9.063 | 2.889 | 19.787 | 11.397 |
| Pbr030984.1 | psbB; photosystem II P680 chlorophyll A apoprotein; K02704 photosystem II CP47 chlorophyll apoprotein | 1.853 | 1.469 | 2.957 | 9.063 | 2.889 | 19.787 | 11.397 |
| Pbr030985.1 | petA; apocytochrome f precursor; K02634 apocytochrome f | 0.371 | 1.069 | 1.549 | 3.375 | 1.926 | 9.36 | 3.419 |
| Pbr030989.1 | atpB; ATP synthase CF1 beta subunit; K02112 F-type H+-transporting ATPase subunit beta [EC:3.6.3.14] | 0.494 | 0.935 | 1.83 | 2.603 | 1.376 | 14.455 | 5.065 |
| Pbr030990.1 | atpE; ATP synthase epsilon chain; K02114 F-type H+-transporting ATPase subunit epsilon [EC:3.6.3.14] | 0 | 0.401 | 0 | 0.675 | 0.275 | 3.436 | 1.646 |
| Pbr030992.1 | psaA; photosystem I P700 chlorophyll a apoprotein A1; K02689 photosystem I P700 chlorophyll a apoprotein A1 | 2.1 | 1.736 | 3.097 | 12.63 | 4.816 | 31.28 | 16.082 |
| Pbr030993.1 | psaB; photosystem I P700 chlorophyll a apoprotein A2; K02690 photosystem I P700 chlorophyll a apoprotein A2 | 1.483 | 1.736 | 5.069 | 12.534 | 4.54 | 30.451 | 20.261 |
| Pbr030994.1 | psbC; photosystem II 44 kDa protein; K02705 photosystem II CP43 chlorophyll apoprotein | 4.571 | 4.274 | 5.209 | 14.462 | 3.44 | 31.635 | 13.296 |
| Pbr030995.1 | psbD; photosystem II protein D2; K02706 photosystem II P680 reaction center D2 protein | 1.977 | 1.603 | 3.661 | 9.063 | 4.678 | 28.436 | 14.436 |
| Pbr030999.1 | atpA; ATPase alpha subunit; K02111 F-type H+-transporting ATPase subunit alpha [EC:3.6.3.14] | 2.718 | 2.271 | 6.758 | 14.655 | 8.668 | 26.659 | 11.65 |
| Pbr031000.1 | psbA; photosystem II protein D1; K02703 photosystem II P680 reaction center D1 protein | 24.339 | 10.952 | 15.769 | 140.38 | 9.906 | 91.826 | 68.253 |
| Pbr031897.1 | hypothetical protein LOC100264091; K02115 F-type H+-transporting ATPase subunit gamma [EC:3.6.3.14] | 17.173 | 49.153 | 12.108 | 41.265 | 32.195 | 17.062 | 16.842 |
| Pbr032155.1 | hypothetical protein; K14332 photosystem I subunit PsaO | 71.164 | 123.95 | 115.59 | 73.275 | 48.155 | 78.437 | 76.23 |
| Pbr032160.2 | hypothetical protein; K14332 photosystem I subunit PsaO | 105.882 | 188.73 | 179.932 | 115.601 | 73.609 | 124.172 | 128.021 |
| Pbr032325.1 | PSBP2; putative oxygen evolving enhancer protein; K02717 photosystem II oxygen-evolving enhancer protein 2 | 3.212 | 9.617 | 2.534 | 4.531 | 7.292 | 4.502 | 4.685 |
| Pbr032910.1 | hypothetical protein LOC100260011; K08903 photosystem II 13kDa protein | 14.455 | 30.587 | 4.365 | 15.523 | 15.822 | 10.071 | 8.231 |
| Pbr033219.1 | photosystem I reaction center subunit N PsaN; K02701 photosystem I subunit PsaN | 65.728 | 113.532 | 46.743 | 81.952 | 65.904 | 45.024 | 39.381 |
| Pbr033256.1 | hypothetical protein LOC100260599; K08914 light-harvesting complex II chlorophyll a/b binding protein 3 | 42.007 | 137.44 | 36.324 | 53.896 | 91.22 | 46.801 | 24.059 |
| Pbr033308.1 | psaA; photosystem I P700 chlorophyll a apoprotein A1; K02689 photosystem I P700 chlorophyll a apoprotein A1 | 1.235 | 0.935 | 1.69 | 5.881 | 1.789 | 11.967 | 4.812 |
| Pbr033650.1 | Oxygen-evolving enhancer protein 3-1, chloroplast precursor, putative; K08901 photosystem II oxygen-evolving enhancer protein 3 | 0.865 | 3.339 | 0.845 | 0.964 | 1.101 | 0.948 | 1.013 |
| Pbr033766.1 | photosystem II oxygen-evolving complex 33 KDa subunit; K02716 photosystem II oxygen-evolving enhancer protein 1 | 187.425 | 448.251 | 239.909 | 253.474 | 277.925 | 262.799 | 190.829 |
| Pbr033791.1 | photosystem II oxygen-evolving complex 33 KDa subunit; K02716 photosystem II oxygen-evolving enhancer protein 1 | 187.425 | 448.251 | 239.909 | 253.474 | 277.925 | 262.799 | 190.829 |
| Pbr034833.1 | hypothetical protein; K02641 ferredoxin--NADP+ reductase [EC:1.18.1.2] | 57.574 | 129.56 | 60.541 | 57.463 | 55.035 | 56.636 | 47.106 |
| Pbr034888.1 | atpA; ATPase alpha subunit; K02111 F-type H+-transporting ATPase subunit alpha [EC:3.6.3.14] | 2.718 | 2.271 | 6.758 | 14.655 | 8.668 | 26.659 | 11.65 |
| Pbr034889.1 | psbA; photosystem II protein D1; K02703 photosystem II P680 reaction center D1 protein | 24.339 | 10.952 | 15.769 | 140.38 | 9.906 | 91.826 | 68.253 |
| Pbr034902.1 | psbA; photosystem II protein D1; K02703 photosystem II P680 reaction center D1 protein | 24.339 | 10.952 | 15.769 | 140.38 | 9.906 | 91.826 | 68.253 |
| Pbr034903.1 | atpA; ATPase alpha subunit | 2.718 | 2.271 | 6.758 | 14.655 | 8.668 | 26.659 | 11.65 |
| Pbr036225.1 | hypothetical protein LOC100252545; K02723 photosystem II PsbY protein | 58.439 | 120.878 | 37.028 | 143.368 | 110.482 | 44.669 | 34.443 |
| Pbr036302.1 | chlorophyll A/B binding protein, putative; K08913 light-harvesting complex II chlorophyll a/b binding protein 2 | 274.527 | 667.568 | 231.884 | 376.306 | 607.308 | 246.804 | 170.315 |
| Pbr037072.1 | Photosystem I reaction center subunit II, chloroplast precursor, putative; K02692 photosystem I subunit II | 69.558 | 146.122 | 77.436 | 65.08 | 78.425 | 84.361 | 101.049 |
| Pbr037237.1 | photosystem II subunit R (PsbR); K03541 photosystem II 10kDa protein | 125.65 | 310.142 | 136.568 | 74.432 | 61.776 | 76.66 | 88.387 |
| Pbr037487.1 | ferredoxin--NADP reductase, putative (EC:1.18.1.2); K02641 ferredoxin--NADP+ reductase [EC:1.18.1.2] | 131.58 | 96.302 | 41.956 | 61.609 | 55.31 | 35.19 | 51.664 |
| Pbr037746.1 | hypothetical protein; K14332 photosystem I subunit PsaO | 63.134 | 112.73 | 62.793 | 38.469 | 48.018 | 41.47 | 34.949 |
| Pbr037913.1 | chlorophyll A/B binding protein, putative; K08915 light-harvesting complex II chlorophyll a/b binding protein 4 | 87.102 | 210.368 | 121.644 | 131.895 | 136.073 | 132.466 | 89.779 |
| Pbr038173.1 | psbB; photosystem II P680 chlorophyll A apoprotein; K02704 photosystem II CP47 chlorophyll apoprotein | 0.371 | 0 | 0.986 | 2.989 | 0.963 | 5.687 | 3.672 |
| Pbr038184.1 | psbB; photosystem II P680 chlorophyll A apoprotein; K02704 photosystem II CP47 chlorophyll apoprotein | 1.73 | 1.202 | 2.816 | 7.81 | 2.477 | 17.773 | 9.877 |
| Pbr038186.1 | petA; apocytochrome f precursor; K02634 apocytochrome f | 0.371 | 1.069 | 1.549 | 3.375 | 1.926 | 9.36 | 3.419 |
| Pbr038188.1 | atpB; ATP synthase CF1 beta subunit; K02112 F-type H+-transporting ATPase subunit beta [EC:3.6.3.14] | 0.494 | 0.935 | 1.83 | 2.603 | 1.376 | 14.455 | 5.065 |
| Pbr039554.1 | chlorophyll A/B binding protein, putative; K08912 light-harvesting complex II chlorophyll a/b binding protein 1 | 372.749 | 1112.212 | 827.856 | 669.696 | 729.898 | 724.297 | 419.013 |
| Pbr039555.1 | Lhcb1-1; light-harvesting complex II protein Lhcb1; K08912 light-harvesting complex II chlorophyll a/b binding protein 1 | 381.027 | 1161.231 | 819.972 | 695.824 | 736.778 | 717.306 | 390.522 |
| Pbr041033.1 | hypothetical protein LOC100243967; K03541 photosystem II 10kDa protein | 374.973 | 536.271 | 480.382 | 211.148 | 110.069 | 276.069 | 254.776 |
| Pbr041230.1 | psbA; photosystem II protein D1; K02703 photosystem II P680 reaction center D1 protein | 24.339 | 10.952 | 15.769 | 140.38 | 9.906 | 91.826 | 68.253 |
| Pbr041231.1 | atpA; ATPase alpha subunit; K02111 F-type H+-transporting ATPase subunit alpha [EC:3.6.3.14] | 2.718 | 2.271 | 6.758 | 14.655 | 8.668 | 26.659 | 11.65 |
| Pbr041234.1 | psbD; photosystem II protein D2; K02706 photosystem II P680 reaction center D2 protein | 1.977 | 1.603 | 3.661 | 9.063 | 4.678 | 28.436 | 14.436 |
| Pbr041235.1 | psbC; photosystem II 44 kDa protein; K02705 photosystem II CP43 chlorophyll apoprotein | 4.571 | 4.274 | 5.209 | 14.462 | 3.44 | 31.635 | 13.296 |
| Pbr041236.2 | psaA; photosystem I P700 chlorophyll a apoprotein A1; K02689 photosystem I P700 chlorophyll a apoprotein A1 | 2.1 | 1.736 | 3.097 | 12.63 | 4.816 | 31.28 | 16.082 |
| Pbr041240.1 | atpE; ATP synthase epsilon chain; K02114 F-type H+-transporting ATPase subunit epsilon [EC:3.6.3.14] | 0 | 0.401 | 0 | 0.675 | 0.275 | 3.436 | 1.646 |
| Pbr041241.1 | atpB; ATP synthase CF1 beta subunit; K02112 F-type H+-transporting ATPase subunit beta [EC:3.6.3.14] | 0.494 | 0.935 | 1.83 | 2.603 | 1.376 | 14.455 | 5.065 |
| Pbr041244.1 | petA; apocytochrome f precursor; K02634 apocytochrome f | 0.371 | 1.069 | 1.549 | 3.375 | 1.926 | 9.36 | 3.419 |
| Pbr041327.1 | Photosystem I reaction center subunit V, chloroplast precursor, putative; K08905 photosystem I subunit V | 62.763 | 208.632 | 58.71 | 123.218 | 106.355 | 69.55 | 42.42 |
| Pbr042101.1 | ferredoxin--NADP reductase, putative (EC:1.18.1.2); K02641 ferredoxin--NADP+ reductase [EC:1.18.1.2] | 66.964 | 51.423 | 23.09 | 27.575 | 28.893 | 18.484 | 20.514 |
| Pbr042127.1 | hypothetical protein LOC100261762; K02698 photosystem I subunit X | 36.941 | 78.537 | 25.906 | 43.483 | 35.91 | 30.806 | 21.274 |
| Pbr042266.1 | psaA; photosystem I P700 chlorophyll a apoprotein A1; K02689 photosystem I P700 chlorophyll a apoprotein A1 | 0.618 | 0.668 | 0.422 | 5.206 | 1.651 | 9.005 | 5.192 |
| Pbr042362.1 | atpE; ATP synthase epsilon chain; | 0 | 0.401 | 0 | 0.675 | 0.275 | 3.436 | 1.646 |
| Pbr042363.1 | atpB; ATP synthase CF1 beta subunit; K02112 F-type H+-transporting ATPase subunit beta [EC:3.6.3.14] | 0.494 | 0.935 | 1.83 | 2.603 | 1.376 | 14.455 | 5.065 |
| Pbr042366.1 | petA; apocytochrome f precursor; K02634 apocytochrome | 0.371 | 1.069 | 1.549 | 3.375 | 1.926 | 9.36 | 3.419 |
| Pbr042367.1 | psbB; photosystem II P680 chlorophyll A apoprotein; K02704 photosystem II CP47 chlorophyll apoprotein | 1.853 | 1.469 | 2.957 | 9.063 | 2.889 | 19.787 | 11.397 |
| Pbr042379.1 | psbB; photosystem II P680 chlorophyll A apoprotein; K02704 photosystem II CP47 chlorophyll apoprotein | 1.853 | 1.469 | 2.957 | 9.063 | 2.889 | 19.787 | 11.397 |
| Pbr042380.1 | petA; apocytochrome f precursor; K02634 apocytochrome f | 0.371 | 1.069 | 1.549 | 3.375 | 1.926 | 9.36 | 3.419 |
| Pbr042383.1 | atpB; ATP synthase CF1 beta subunit; K02112 F-type H+-transporting ATPase subunit beta [EC:3.6.3.14] | 0.494 | 0.935 | 1.83 | 2.603 | 1.376 | 14.455 | 5.065 |
| Pbr042384.1 | atpE; ATP synthase epsilon chain; K02114 F-type H+-transporting ATPase subunit epsilon [EC:3.6.3.14] | 0 | 0.401 | 0 | 0.675 | 0.275 | 3.436 | 1.646 |
| Pbr042856.1 | photosystem I reaction center subunit IV A, chloroplast precursor; K02693 photosystem I subunit IV | 128.739 | 230.002 | 116.576 | 105.092 | 79.25 | 89.574 | 73.571 |
|  |  |  |  |  |  |  |  |  |
| Hormone/signaling | | | | | | | | |
| Gene ID | Gene Description | TPM (transcripts per million clean tag) | | | | | | |
|  |  | C1 | C2 | C3 | C4 | C5 | C6 | C7 |
| Pbr000392.1 | Auxin-repressed 12.5 kDa protein, putative | 494.692 | 841.205 | 1843.67 | 736.607 | 544.156 | 1161.505 | 1883.596 |
| Pbr000415.1 | Auxin response factor, putative; K14486 auxin response factor | 3.089 | 6.945 | 3.942 | 5.978 | 7.842 | 4.739 | 3.419 |
| Pbr001262.1 | SAUR18; SAUR family protein; K14488 SAUR family protein | 4.571 | 6.678 | 10.7 | 7.424 | 3.577 | 8.057 | 11.143 |
| Pbr001267.1 | SAUR18; SAUR family protein; K14488 SAUR family protein | 4.571 | 6.678 | 10.7 | 7.424 | 3.577 | 8.057 | 11.143 |
| Pbr002342.1 | hypothetical protein LOC100242923 | 87.349 | 76.4 | 60.681 | 82.531 | 99.475 | 27.133 | 31.277 |
| Pbr003287.1 | Auxin response factor, putative | 1.73 | 2.004 | 2.534 | 2.314 | 2.477 | 1.066 | 0.76 |
| Pbr003402.1 | Indole-3-acetic acid-induced protein ARG7, putative; K14488 SAUR family protein | 1.606 | 1.336 | 2.112 | 0.482 | 0.963 | 0.948 | 0.633 |
| Pbr004491.1 | SAUR1; SAUR family protein; K14488 SAUR family protein | 0.741 | 0.134 | 0.141 | 1.061 | 0.138 | 0.237 | 0 |
| Pbr005563.1 | Auxin response factor, putative; K14486 auxin response factor | 15.444 | 23.508 | 8.729 | 21.018 | 52.421 | 6.517 | 3.925 |
| Pbr005564.1 | Auxin response factor, putative; K14486 auxin response factor | 16.185 | 26.713 | 9.433 | 28.442 | 53.246 | 6.991 | 3.799 |
| Pbr005852.1 | hypothetical protein LOC100247221; K14488 SAUR family protein | 2.347 | 3.606 | 1.126 | 1.543 | 3.302 | 2.014 | 4.052 |
| Pbr005854.1 | Auxin response factor, putative; K14486 auxin response factor | 34.717 | 55.03 | 19.57 | 52.45 | 112.546 | 13.863 | 7.344 |
| Pbr007550.1 | hypothetical protein LOC100250741; K14487 auxin responsive GH3 gene family | 77.466 | 37.265 | 1.971 | 17.065 | 35.773 | 4.858 | 5.572 |
| Pbr007631.1 | Bipolar kinesin KRP-130, putative | 3.089 | 12.021 | 4.928 | 3.375 | 18.299 | 11.967 | 6.458 |
| Pbr007826.1 | SAUR24; SAUR family protein; K14488 SAUR family protein | 1.483 | 1.469 | 1.126 | 2.025 | 0.963 | 0.237 | 0.76 |
| Pbr008163.2 | PtrAUX8; auxin influx carrier component; K13946 auxin influx carrier (AUX1 LAX family) | 2.347 | 4.274 | 4.224 | 4.531 | 6.742 | 3.91 | 5.952 |
| Pbr008352.1 | Auxin response factor, putative | 16.432 | 14.158 | 11.545 | 9.641 | 7.842 | 6.28 | 6.965 |
| Pbr008550.1 | Auxin response factor, putative | 7.289 | 15.894 | 11.686 | 7.52 | 14.722 | 5.095 | 7.598 |
| Pbr009498.1 | LAX1; AUX-1-like protein; K13946 auxin influx carrier (AUX1 LAX family) | 45.219 | 52.091 | 77.154 | 72.889 | 87.23 | 41.588 | 38.875 |
| Pbr009914.1 | SAUR69; SAUR family protein; K14488 SAUR family protein | 0.741 | 0.267 | 4.365 | 1.35 | 0.413 | 7.465 | 5.065 |
| Pbr009923.1 | SAUR69; SAUR family protein; K14488 SAUR family protein | 0.247 | 0.267 | 0.141 | 1.157 | 0.413 | 0 | 0.127 |
| Pbr009926.1 | SAUR69; SAUR family protein; K14488 SAUR family protein | 0.247 | 0.267 | 0.141 | 1.157 | 0.413 | 0 | 0.127 |
| Pbr009936.1 | hypothetical protein LOC100256532; K14488 SAUR family protein | 4.201 | 2.538 | 5.772 | 6.171 | 0.55 | 1.066 | 4.179 |
| Pbr009937.1 | SAUR69; SAUR family protein; K14488 SAUR family protein | 0.865 | 0.267 | 4.505 | 1.735 | 0.688 | 9.005 | 7.091 |
| Pbr011330.1 | LAX1; AUX-1-like protein; K13946 auxin influx carrier (AUX1 LAX family) | 20.386 | 23.508 | 37.169 | 35.577 | 46.229 | 31.872 | 24.059 |
| Pbr011750.1 | hypothetical protein; K07088 | 155.055 | 24.309 | 10.278 | 44.061 | 9.356 | 2.962 | 8.864 |
| Pbr011928.1 | hypothetical protein LOC100263801 | 26.069 | 44.745 | 38.577 | 29.406 | 27.38 | 14.455 | 17.095 |
| Pbr012348.1 | PtrAUX2; auxin influx carrier component; K13946 auxin influx carrier (AUX1 LAX family) | 104.399 | 105.919 | 98.132 | 164.29 | 115.16 | 41.233 | 59.642 |
| Pbr012651.1 | similar to auxin response factor 8 | 58.439 | 71.058 | 53.36 | 59.584 | 85.717 | 28.91 | 22.413 |
| Pbr013424.1 | PIN6; auxin efflux carrier component; K13947 auxin efflux carrier | 111.565 | 52.759 | 32.523 | 89.183 | 27.93 | 6.28 | 19.501 |
| Pbr013430.1 | PIN6; auxin efflux carrier component; K13947 auxin efflux carrier family | 111.565 | 52.759 | 32.523 | 89.183 | 27.93 | 6.28 | 19.501 |
| Pbr013531.1 | hypothetical protein LOC100241753; K14488 SAUR family protein | 12.602 | 1.736 | 3.379 | 7.617 | 4.953 | 2.37 | 2.912 |
| Pbr013674.1 | auxin efflux carrier component, auxin transport protein | 62.145 | 40.471 | 56.176 | 50.81 | 22.427 | 20.498 | 25.579 |
| Pbr013892.1 | Auxin efflux carrier component, putative; K13947 auxin efflux carrier family | 3.83 | 10.018 | 3.097 | 2.989 | 9.356 | 2.725 | 1.14 |
| Pbr016201.1 | hypothetical protein LOC100245251; K14486 auxin response factor | 4.324 | 6.678 | 6.758 | 6.653 | 14.447 | 4.028 | 5.065 |
| Pbr016230.1 | hypothetical protein LOC100260521 | 414.632 | 804.34 | 1992.487 | 895.016 | 461.604 | 1258.189 | 2030.232 |
| Pbr016467.1 | Auxin response factor, putative | 19.891 | 9.884 | 7.04 | 7.906 | 4.678 | 2.725 | 3.546 |
| Pbr018370.1 | hypothetical protein LOC100265555 | 18.409 | 41.673 | 23.231 | 23.718 | 50.082 | 12.441 | 12.03 |
| Pbr018404.1 | SAUR24; SAUR family protein; K14488 SAUR family protein | 5.189 | 6.278 | 4.646 | 9.641 | 3.99 | 2.725 | 2.279 |
| Pbr018494.1 | Auxin-induced protein X10A, putative; K14488 SAUR family protein | 85.99 | 52.893 | 15.628 | 13.787 | 12.933 | 3.555 | 8.737 |
| Pbr018629.1 | hypothetical protein LOC100248869; K14487 auxin responsive GH3 gene family | 1.359 | 0.267 | 0 | 0.771 | 0 | 0 | 0 |
| Pbr021104.1 | hypothetical protein LOC100242923 | 179.517 | 189.665 | 176.975 | 181.645 | 179.826 | 53.792 | 73.191 |
| Pbr021158.1 | hypothetical protein LOC100250242; K14487 auxin responsive GH3 gene family | 2.1 | 4.274 | 1.126 | 6.074 | 11.557 | 1.896 | 1.646 |
| Pbr021608.1 | PtrAUX6; auxin influx carrier component; K13946 auxin influx carrier (AUX1 LAX family) | 1.73 | 6.411 | 2.253 | 10.316 | 29.444 | 5.332 | 1.773 |
| Pbr021609.1 | PtrAUX6; auxin influx carrier component; K13946 auxin influx carrier (AUX1 LAX family) | 1.73 | 6.411 | 2.253 | 10.316 | 29.444 | 5.332 | 1.773 |
| Pbr022122.1 | hypothetical protein LOC100252339; K14488 SAUR family protein | 1.359 | 0.401 | 0.141 | 2.121 | 2.064 | 0 | 0 |
| Pbr022379.1 | SAUR38; SAUR family protein; K14488 SAUR family protein | 4.448 | 8.281 | 5.069 | 23.043 | 6.879 | 2.37 | 5.698 |
| Pbr023255.1 | Auxin-induced protein 6B, putative; K14488 SAUR family protein | 8.278 | 8.281 | 8.729 | 23.236 | 7.017 | 7.938 | 12.156 |
| Pbr023257.1 | hypothetical protein LOC100257133; K14488 SAUR family protein | 3.089 | 3.606 | 2.393 | 4.435 | 3.302 | 1.066 | 6.838 |
| Pbr023265.1 | hypothetical protein LOC100256532; K14488 SAUR family protein | 0.124 | 0 | 0.422 | 0.289 | 0 | 0 | 1.899 |
| Pbr025212.1 | Indole-3-acetic acid-amido synthetase GH3.3, putative; K14487 auxin responsive GH3 gene family | 0 | 0.668 | 0.282 | 1.639 | 2.614 | 0 | 0 |
| Pbr025597.1 | hypothetical protein LOC100243320 | 3.83 | 14.292 | 11.123 | 5.399 | 17.198 | 11.138 | 9.37 |
| Pbr026615.1 | hypothetical protein LOC100264303; K14486 auxin response factor | 12.726 | 26.981 | 23.512 | 17.837 | 24.766 | 22.749 | 17.855 |
| Pbr026872.1 | Indole-3-acetic acid-amido synthetase GH3.3, putative; K14487 auxin responsive GH3 gene family | 1.112 | 0.534 | 0.563 | 1.157 | 1.513 | 0.474 | 0.253 |
| Pbr026873.1 | Indole-3-acetic acid-amido synthetase GH3.3, putative; K14487 auxin responsive GH3 gene family | 1.112 | 0.534 | 0.563 | 1.157 | 1.513 | 0.474 | 0.253 |
| Pbr026939.1 | SAUR1; SAUR family protein; K14488 SAUR family protein | 2.595 | 1.202 | 1.126 | 6.171 | 0.275 | 0.474 | 0.38 |
| Pbr027334.1 | hypothetical protein LOC100250242; K14487 auxin responsive GH3 gene family | 1.73 | 1.336 | 0.282 | 5.881 | 9.631 | 0.474 | 0.76 |
| Pbr027421.1 | hypothetical protein LOC100265555 | 18.409 | 41.673 | 23.231 | 23.718 | 50.082 | 12.441 | 12.03 |
| Pbr027725.1 | Auxin response factor, putative | 7.537 | 16.562 | 14.502 | 11.281 | 16.098 | 6.398 | 5.825 |
| Pbr028075.1 | Auxin-induced protein 6B, putative; K14488 SAUR family protein | 5.436 | 7.613 | 0.282 | 4.242 | 8.255 | 0.474 | 0.38 |
| Pbr028076.1 | Auxin response factor, putative; K14486 auxin response factor | 98.716 | 57.968 | 63.356 | 117.144 | 58.887 | 10.071 | 13.929 |
| Pbr028379.1 | PIN7; auxin efflux carrier component; K13947 auxin efflux carrier family | 11.119 | 32.724 | 10.278 | 13.016 | 44.441 | 11.73 | 9.877 |
| Pbr028490.1 | auxin efflux carrier component, auxin transport protein | 55.968 | 38.067 | 37.732 | 34.516 | 20.088 | 17.773 | 19.754 |
| Pbr028598.1 | hypothetical protein LOC100244520; K13947 auxin efflux carrier family | 7.784 | 7.48 | 4.646 | 8.195 | 4.54 | 2.844 | 3.039 |
| Pbr028713.1 | PIN6; auxin efflux carrier component; K13947 auxin efflux carrier family | 66.47 | 50.622 | 28.581 | 51.775 | 48.293 | 27.37 | 43.813 |
| Pbr029742.1 | LAX1; AUX-1-like protein; K13946 auxin influx carrier (AUX1 LAX family) | 30.64 | 37.399 | 31.819 | 57.945 | 39.763 | 14.337 | 20.007 |
| Pbr030571.1 | K14487 auxin responsive GH3 gene family | 26.316 | 91.093 | 8.448 | 11.666 | 30.957 | 11.848 | 11.776 |
| Pbr030587.1 | hypothetical protein LOC100250741; K14487 auxin responsive GH3 gene family | 26.316 | 91.093 | 8.448 | 11.666 | 30.957 | 11.848 | 11.776 |
| Pbr030688.1 | Indole-3-acetic acid-amido synthetase GH3.5, putative; K14487 auxin responsive GH3 gene family | 1.853 | 5.076 | 0.986 | 2.892 | 4.678 | 2.607 | 1.013 |
| Pbr030776.1 | PIN6; auxin efflux carrier component; K13947 auxin efflux carrier family | 23.474 | 15.494 | 10.419 | 17.162 | 14.859 | 10.545 | 15.575 |
| Pbr030777.1 | PIN6; auxin efflux carrier component; K13947 auxin efflux carrier family | 33.111 | 27.248 | 15.487 | 25.743 | 26.142 | 15.048 | 23.046 |
| Pbr031621.1 | amino acid transporter, putative; K13946 auxin influx carrier (AUX1 LAX family) | 0.988 | 4.675 | 2.253 | 11.184 | 34.809 | 4.147 | 0.886 |
| Pbr033508.2 | PtrAUX8; auxin influx carrier component; K13946 auxin influx carrier (AUX1 LAX family) | 2.1 | 6.945 | 6.195 | 5.496 | 7.292 | 4.739 | 6.711 |
| Pbr033588.1 | hypothetical protein | 37.312 | 70.39 | 44.631 | 43.29 | 72.371 | 33.768 | 27.858 |
| Pbr033897.1 | Auxin efflux carrier component, putative; K13947 auxin efflux carrier family | 3.459 | 9.884 | 3.52 | 3.278 | 9.218 | 2.488 | 1.266 |
| Pbr034084.1 | hypothetical protein LOC100250437; K14487 auxin responsive GH3 gene family | 0.371 | 0.801 | 0 | 0.964 | 2.339 | 0.118 | 0.127 |
| Pbr034085.1 | Indole-3-acetic acid-amido synthetase GH3.17, putative; K14487 | 0.124 | 0.668 | 0 | 0.675 | 1.926 | 0.118 | 0.127 |
| Pbr035230.1 | hypothetical protein LOC100259491; K13947 auxin efflux carrier family | 2.1 | 4.942 | 5.913 | 2.7 | 6.329 | 1.54 | 2.912 |
| Pbr035975.1 | LAX1; AUX-1-like protein; K13946 auxin influx carrier (AUX1 LAX family) | 31.382 | 37.799 | 32.805 | 59.584 | 40.175 | 13.981 | 20.261 |
| Pbr036005.1 | Indole-3-acetic acid-induced protein ARG7, putative; K14488 SAUR family protein | 1.483 | 1.469 | 1.971 | 0.289 | 0.963 | 0.829 | 0.76 |
| Pbr037271.1 | Auxin response factor, putative | 1.235 | 3.473 | 1.126 | 1.543 | 2.889 | 1.54 | 2.026 |
| Pbr037343.1 | auxin:hydrogen symporter, putative; K07088 | 24.463 | 6.812 | 5.913 | 11.377 | 15.135 | 8.768 | 7.218 |
| Pbr037398.1 | hypothetical protein LOC100266455; K14488 SAUR family protein | 0.741 | 0.935 | 1.83 | 0.578 | 1.513 | 0.592 | 0.253 |
| Pbr037412.1 | Auxin response factor, putative | 11.861 | 32.19 | 13.798 | 17.451 | 35.91 | 13.152 | 13.676 |
| Pbr037834.1 | GH3-7; GH3 family protein; K14487 auxin responsive GH3 gene family | 5.56 | 2.137 | 0.282 | 3.664 | 0.275 | 0 | 0 |
| Pbr038852.1 | PIN1; auxin efflux carrier component; K13947 auxin efflux carrier family | 5.807 | 17.898 | 4.646 | 11.666 | 25.729 | 6.635 | 5.698 |
| Pbr039066.1 | hypothetical protein LOC100265118 | 4.818 | 14.826 | 0.986 | 2.41 | 2.477 | 1.777 | 1.773 |
| Pbr039380.2 | hypothetical protein; K00001 alcohol dehydrogenase [EC:1.1.1.1] | 38.547 | 82.678 | 46.039 | 13.884 | 21.739 | 56.28 | 28.111 |
| Pbr039624.1 | hypothetical protein LOC100242923 | 179.023 | 188.73 | 175.004 | 179.91 | 179.276 | 53.437 | 72.811 |
| Pbr040072.1 | hypothetical protein LOC100241753; K14488 SAUR family protein | 33.482 | 18.833 | 41.674 | 23.911 | 4.265 | 12.678 | 27.098 |
| Pbr040903.1 | hypothetical protein LOC100256532; K14488 SAUR family protein | 0.618 | 1.87 | 2.393 | 10.22 | 1.376 | 0.355 | 1.773 |
| Pbr040904.2 | SAUR69; SAUR family protein; K14488 SAUR family protein | 1.235 | 1.202 | 0.845 | 3.76 | 1.789 | 0 | 0.127 |
| Pbr040905.1 | hypothetical protein LOC100256532; K14488 SAUR family protein | 0.371 | 0.534 | 0.563 | 1.543 | 0 | 0.118 | 0.253 |
| Pbr040906.1 | SAUR82; SAUR family protein; K14488 SAUR family protein | 0.618 | 1.202 | 0.282 | 2.7 | 0.55 | 0.118 | 0.253 |
| Pbr041132.1 | hypothetical protein LOC100250242; K14487 auxin responsive GH3 gene family | 0.988 | 0.801 | 0.282 | 4.917 | 6.329 | 0.355 | 0.507 |
| Pbr041251.1 | hypothetical protein LOC100263801 | 11.861 | 30.72 | 27.595 | 8.292 | 18.574 | 16.351 | 15.575 |
| Pbr041836.1 | hypothetical protein LOC100243320 | 2.595 | 11.086 | 7.321 | 7.038 | 16.235 | 6.517 | 6.711 |
| Pbr042214.1 | SAUR69; SAUR family protein; K14488 SAUR family protein | 0.247 | 0.267 | 0.141 | 1.061 | 0.413 | 0 | 0.127 |
| Pbr042223.1 | SAUR69; SAUR family protein; K14488 SAUR family protein | 4.077 | 1.87 | 6.758 | 6.267 | 0.275 | 1.185 | 4.939 |
| Pbr042224.1 | SAUR69; SAUR family protein; K14488 SAUR family protein | 0.865 | 0.267 | 4.505 | 1.735 | 0.688 | 9.005 | 7.091 |
| Pbr042229.1 | SAUR38; SAUR family protein; K14488 SAUR family protein | 4.201 | 8.014 | 4.505 | 21.983 | 6.467 | 2.014 | 4.939 |
| Pbr042396.1 | hypothetical protein | 5.189 | 8.682 | 1.267 | 2.41 | 3.577 | 2.014 | 2.406 |
| Pbr000396.1 | similar to putative ethylene responsive element binding protein 1 | 25.945 | 52.358 | 26.61 | 16.776 | 43.065 | 38.508 | 46.599 |
| Pbr000435.1 | Ethylene-responsive transcription factor, putative | 14.826 | 4.408 | 3.238 | 8.195 | 9.769 | 2.37 | 4.179 |
| Pbr001287.2 | methylenetetrahydrofolate dehydrogenase, putative (EC:3.5.4.9) | 6.054 | 5.343 | 1.83 | 5.592 | 9.081 | 2.607 | 3.925 |
| Pbr002199.1 | ethylene receptor, putative; K14509 ethylene receptor | 55.968 | 114.6 | 40.126 | 30.563 | 117.912 | 36.019 | 28.871 |
| Pbr004323.1 | ethylene receptor, putative; K14509 ethylene receptor [EC:2.7.13.-] | 10.008 | 8.148 | 11.404 | 8.002 | 20.776 | 12.204 | 7.724 |
| Pbr004535.1 | hypothetical protein LOC100248434 | 30.146 | 26.313 | 22.386 | 17.547 | 25.041 | 13.27 | 29.378 |
| Pbr004574.1 | hypothetical protein LOC100248434 | 22.61 | 20.302 | 19.148 | 14.366 | 20.363 | 10.427 | 24.059 |
| Pbr005656.1 | s-adenosyl-l-methionine:delta24-sterol-C-methyltransferase, putative (EC:2.1.1.143); K08242 24-methylenesterol C-methyltransferase [EC:2.1.1.143] | 18.285 | 35.128 | 23.231 | 12.92 | 31.232 | 23.223 | 24.693 |
| Pbr005788.1 | s-adenosyl-l-methionine:delta24-sterol-C-methyltransferase, putative (EC:2.1.1.143); K08242 24-methylenesterol C-methyltransferase [EC:2.1.1.143] | 280.704 | 488.855 | 314.951 | 192.058 | 461.466 | 357.587 | 389.762 |
| Pbr008360.1 | hypothetical protein LOC100256742; K14513 ethylene-insensitive protein 2 | 33.976 | 33.792 | 38.577 | 34.131 | 35.91 | 14.1 | 17.728 |
| Pbr010445.1 | hypothetical protein LOC100250496; K14514 ethylene-insensitive protein 3 | 7.166 | 12.288 | 17.74 | 12.052 | 11.42 | 10.545 | 14.436 |
| Pbr010446.1 | hypothetical protein | 59.674 | 80.007 | 115.309 | 101.814 | 71.82 | 65.404 | 123.209 |
| Pbr010447.1 | hypothetical protein | 59.674 | 80.007 | 115.309 | 101.814 | 71.82 | 65.404 | 123.209 |
| Pbr010448.1 | hypothetical protein LOC100250496 | 7.166 | 12.288 | 17.74 | 12.052 | 11.42 | 10.545 | 14.436 |
| Pbr011796.1 | ethylene receptor, putative; K14509 ethylene receptor [EC:2.7.13.-] | 55.968 | 114.6 | 40.126 | 30.563 | 117.912 | 36.019 | 28.871 |
| Pbr016185.1 | Ethylene-responsive transcription factor, putative | 114.777 | 47.416 | 40.83 | 77.324 | 81.727 | 23.341 | 50.018 |
| Pbr016224.1 | similar to putative ethylene responsive element binding protein 1 | 20.262 | 56.632 | 31.115 | 24.296 | 48.293 | 44.787 | 59.895 |
| Pbr016462.1 | EIN2 -like protein, nramp transporter; K14513 ethylene-insensitive protein 2 | 46.207 | 30.72 | 36.324 | 36.927 | 30.819 | 14.455 | 15.322 |
| Pbr019396.1 | ETR4; ethylene receptor 4; K14509 ethylene receptor [EC:2.7.13.-] | 37.188 | 30.587 | 27.173 | 38.469 | 28.343 | 15.521 | 17.855 |
| Pbr022706.1 | ETR1; ethylene receptor 1; K14509 ethylene receptor [EC:2.7.13.-] | 61.528 | 101.377 | 69.692 | 51.967 | 69.206 | 36.019 | 72.052 |
| Pbr023044.1 | Ethylene-responsive transcription factor 1B, putative; K14516 ethylene-responsive transcription factor 1 | 22.115 | 18.299 | 16.754 | 79.638 | 87.505 | 25 | 49.765 |
| Pbr023072.1 | ethylene receptor, putative; K14509 ethylene receptor [EC:2.7.13.-] | 43.613 | 131.697 | 63.216 | 32.01 | 91.77 | 43.128 | 33.683 |
| Pbr024467.1 | Ethylene-responsive transcription factor, putative; K09286 EREBP-like factor | 70.423 | 59.17 | 29.144 | 29.31 | 47.192 | 22.868 | 24.946 |
| Pbr024739.1 | hypothetical protein LOC100250496; K14514 ethylene-insensitive protein 3 | 332.224 | 266.466 | 426.599 | 375.342 | 303.104 | 179.504 | 341.896 |
| Pbr025056.1 | ETR4; ethylene receptor 4; K14509 ethylene receptor [EC:2.7.13.-] | 50.655 | 43.009 | 41.674 | 61.512 | 53.109 | 28.436 | 24.819 |
| Pbr025988.1 | Ethylene-responsive transcription factor, putative | 0.618 | 0 | 0 | 0.578 | 1.789 | 0 | 0 |
| Pbr026588.2 | methylenetetrahydrofolate dehydrogenase, putative (EC:3.5.4.9) | 5.56 | 7.613 | 2.675 | 5.688 | 10.869 | 4.739 | 7.091 |
| Pbr030542.1 | Ethylene-responsive transcription factor 1B, putative; K14516 | 4.818 | 2.271 | 2.112 | 35.673 | 12.245 | 2.844 | 4.939 |
| Pbr031284.1 | MTHFR2; methylenetetrahydrofolate reductase (EC:1.5.1.20) | 8.648 | 8.548 | 25.202 | 8.388 | 13.621 | 27.133 | 19.627 |
| Pbr035774.1 | Ethylene-responsive transcription factor, putative; K09286 EREBP-like factor | 4.695 | 5.877 | 2.393 | 3.857 | 3.44 | 5.687 | 13.929 |
| Pbr035788.1 | Ethylene-responsive transcription factor, putative; K09286 EREBP-like factor | 5.066 | 6.812 | 2.112 | 4.242 | 3.99 | 6.517 | 15.829 |
| Pbr001095.1 | ACS3; 1-aminocyclopropane-1-carboxylate (EC:4.4.1.14) | 0 | 0 | 2.253 | 0.193 | 0 | 1.185 | 1.773 |
| Pbr002233.1 | acc synthase, putative (EC:4.4.1.14); K01762 1-aminocyclopropane-1-carboxylate synthase [EC:4.4.1.14] | 38.3 | 17.631 | 12.249 | 14.173 | 30.269 | 5.213 | 4.939 |
| Pbr007947.1 | ACS2; 1-aminocyclopropane-1-carboxylate; K01762 1-aminocyclopropane-1-carboxylate synthase [EC:4.4.1.14] | 8.031 | 1.336 | 0 | 4.628 | 0.413 | 0 | 0 |
| Pbr007959.1 | ACS2; 1-aminocyclopropane-1-carboxylate; K01762 1-aminocyclopropane-1-carboxylate synthase [EC:4.4.1.14] | 1.483 | 0.534 | 0 | 0.964 | 0.138 | 0 | 0 |
| Pbr029891.1 | ACS3; 1-aminocyclopropane-1-carboxylate (EC:4.4.1.14); K01762 1-aminocyclopropane-1-carboxylate synthase [EC:4.4.1.14] | 0.124 | 0.668 | 9.433 | 1.832 | 0.275 | 6.28 | 13.929 |
| Pbr030234.1 | ACS3; 1-aminocyclopropane-1-carboxylate (EC:4.4.1.14); K01762 1-aminocyclopropane-1-carboxylate synthase [EC:4.4.1.14] | 0.247 | 0.668 | 10.278 | 2.314 | 0.138 | 6.754 | 17.601 |
| Pbr032688.1 | ACS2; 1-aminocyclopropane-1-carboxylate; K01762 | 45.219 | 6.411 | 0 | 5.014 | 4.128 | 0.355 | 0.253 |
| Pbr036692.1 | acc synthase, putative (EC:4.4.1.14); K01762 1-aminocyclopropane-1-carboxylate synthase [EC:4.4.1.14] | 38.3 | 18.833 | 11.123 | 15.041 | 29.581 | 3.91 | 4.052 |
| Pbr032690.1 | ACS2; 1-aminocyclopropane-1-carboxylate; K01762 1-aminocyclopropane-1-carboxylate synthase [EC:4.4.1.14] | 2.347 | 0.534 | 0.141 | 0.096 | 0.275 | 0.118 | 0 |
| Pbr000093.1 | ACO1; 1-aminocyclopropane-1-carboxylic acid oxidase 1; K05933 aminocyclopropanecarboxylate oxidase [EC:1.14.17.4] | 19.027 | 37.933 | 25.906 | 35.095 | 43.202 | 23.223 | 14.309 |
| Pbr005179.1 | ACO3; 1-aminocyclopropane-1-carboxylate (EC:1.14.11.9); K05933 aminocyclopropanecarboxylate oxidase [EC:1.14.17.4] | 701.143 | 971.299 | 514.031 | 1149.744 | 1835.409 | 697.045 | 531.079 |
| Pbr031954.1 | ACO7; 1-aminocyclopropane-1-carboxylate (EC:1.14.11.9); K05933 aminocyclopropanecarboxylate oxidase [EC:1.14.17.4] | 9.266 | 14.425 | 71.381 | 86.677 | 17.611 | 7.228 | 7.724 |
| Pbr011796.1 | ethylene receptor, putative; K14509 ethylene receptor [EC:2.7.13.-] | 55.968 | 114.6 | 40.126 | 30.563 | 117.912 | 36.019 | 28.871 |
| Pbr025145.1 | zeaxanthin epoxidase, putative (EC:1.14.13.90); K09838 zeaxanthin epoxidase [EC:1.14.13.90] | 147.147 | 82.277 | 227.801 | 156.866 | 18.437 | 27.725 | 49.892 |
| Pbr035432.1 | zeaxanthin epoxidase, putative (EC:1.14.13.90); K09838 zeaxanthin epoxidase [EC:1.14.13.90] | 35.582 | 29.919 | 58.006 | 50.521 | 17.886 | 9.834 | 16.842 |
| Pbr040200.1 | zeaxanthin epoxidase, putative (EC:1.14.13.90); K09838 zeaxanthin epoxidase [EC:1.14.13.90] | 32.741 | 28.583 | 51.952 | 46.183 | 18.437 | 9.953 | 14.562 |
| Pbr000140.1 | gaba(A) receptor-associated protein, putative; K08341 GABA(A) receptor-associated protein (autophagy-related protein 8) | 39.659 | 32.724 | 53.36 | 48.882 | 20.363 | 24.289 | 29.631 |
| Pbr004279.1 | hydroxyacyl-ACP Dehydrase (EC:4.2.1.-); K02372 3R-hydroxymyristoyl ACP dehydrase [EC:4.2.1.-] | 28.911 | 38.868 | 18.303 | 26.9 | 39.35 | 26.541 | 20.134 |
| Pbr007589.1 | hypothetical protein; K14432 ABA responsive element binding factor | 1.977 | 3.873 | 4.787 | 2.507 | 2.614 | 6.28 | 5.952 |
| Pbr013362.1 | GABA-specific permease, putative | 42.13 | 20.836 | 15.487 | 44.929 | 18.299 | 4.028 | 7.598 |
| Pbr014010.1 | gaba(A) receptor-associated protein, putative; K08341 GABA(A) receptor-associated protein (autophagy-related protein 8) | 34.347 | 35.529 | 48.996 | 35.288 | 19.675 | 47.394 | 44.826 |
| Pbr014011.1 | gaba(A) receptor-associated protein, putative; K08341 GABA(A) receptor-associated protein (autophagy-related protein 8) | 34.347 | 35.529 | 48.996 | 35.288 | 19.675 | 47.394 | 44.826 |
| Pbr018094.1 | glutamate receptor 3 plant, putative | 16.432 | 28.583 | 23.231 | 23.236 | 40.726 | 13.981 | 16.335 |
| Pbr018095.1 | hypothetical protein LOC100255191 | 39.165 | 37.799 | 46.18 | 59.584 | 63.152 | 17.417 | 18.741 |
| Pbr018096.3 | glutamate receptor 3 plant, putative | 8.648 | 14.292 | 8.588 | 11.473 | 18.437 | 6.517 | 7.471 |
| Pbr028554.1 | hypothetical protein; K08341 GABA(A) receptor-associated protein (autophagy-related protein 8) | 30.393 | 39.002 | 51.952 | 33.938 | 18.299 | 34.005 | 40.521 |
| Pbr031901.1 | hypothetical protein LOC100246909; K08341 GABA(A) receptor-associated protein (autophagy-related protein 8) | 49.543 | 43.409 | 64.342 | 59.584 | 23.115 | 30.924 | 38.115 |
| Pbr040390.1 | DNA binding protein, putative; K14432 ABA responsive element binding factor | 19.521 | 29.251 | 31.397 | 11.184 | 26.967 | 24.052 | 19.501 |
| Pbr042917.1 | ABA3; ABA3 (ABA DEFICIENT 3); Mo-molybdopterin cofactor sulfurase/ selenocysteine lyase | 2.471 | 1.069 | 1.267 | 2.121 | 1.513 | 1.303 | 0.633 |
|  |  |  |  |  |  |  |  |  |
| Cell wall modification | | | | | | | | |
| Gene ID | Gene Description | TPM (transcripts per million clean tag) | | | | | | |
|  |  | C1 C2 C3 C4 C5 C6 C7 | | | | | | |
| Pbr001831.1 | hypothetical protein LOC100255960 | 26.81 | 8.815 | 1.126 | 9.063 | 5.228 | 2.133 | 2.153 |
| Pbr002929.1 | Alpha-expansin 8 precursor, putative | 44.231 | 21.104 | 25.202 | 40.591 | 25.591 | 85.783 | 63.567 |
| Pbr002980.1 | Alpha-expansin 8 precursor, putative | 50.285 | 24.71 | 28.722 | 46.183 | 30.682 | 101.423 | 78.763 |
| Pbr003748.1 | Alpha-expansin 8 precursor, putative | 49.42 | 30.72 | 47.165 | 34.806 | 39.35 | 142.537 | 276.176 |
| Pbr003883.1 | Alpha-expansin 4 precursor, putative | 15.197 | 21.237 | 5.491 | 30.756 | 81.589 | 7.82 | 6.585 |
| Pbr004072.1 | Alpha-expansin 4 precursor, putative | 8.278 | 15.627 | 4.365 | 12.823 | 43.065 | 7.702 | 5.698 |
| Pbr005347.1 | Alpha-expansin 4 precursor, putative | 7.907 | 14.959 | 4.928 | 13.016 | 43.202 | 7.346 | 5.572 |
| Pbr005798.1 | PtrEXPA17; alpha-expansin | 0.618 | 0.668 | 0.563 | 5.11 | 1.513 | 0.829 | 0.127 |
| Pbr006644.1 | PtEXPA16; hypothetical protein | 0.618 | 1.736 | 0.282 | 0.868 | 2.477 | 0.711 | 0.633 |
| Pbr006866.1 | PtrEXPA22; hypothetical protein | 0.618 | 1.336 | 0.422 | 1.157 | 3.164 | 0.711 | 1.266 |
| Pbr006867.1 | PtrEXPA22; hypothetical protein | 0.618 | 1.336 | 0.422 | 1.157 | 3.164 | 0.711 | 1.266 |
| Pbr007290.1 | hypothetical protein LOC100258655 | 5.313 | 10.819 | 0.986 | 3.567 | 4.265 | 2.607 | 3.039 |
| Pbr008452.1 | hypothetical protein LOC100249605 | 7.289 | 11.754 | 9.433 | 7.713 | 18.849 | 12.796 | 12.283 |
| Pbr008469.1 | hypothetical protein LOC100249605 | 7.289 | 11.754 | 9.433 | 7.713 | 18.849 | 12.796 | 12.283 |
| Pbr009385.1 | PtEXPA7; hypothetical protein | 81.913 | 94.031 | 40.689 | 103.356 | 161.665 | 126.66 | 58.376 |
| Pbr011421.1 | Alpha-expansin 15 precursor, putative | 7.66 | 3.206 | 0.845 | 3.953 | 6.879 | 2.133 | 2.406 |
| Pbr012636.1 | Alpha-expansin 13 precursor, putative | 7.042 | 17.764 | 12.108 | 8.292 | 20.776 | 17.536 | 14.562 |
| Pbr012700.1 | hypothetical protein LOC100264455 | 0.247 | 1.736 | 1.69 | 2.314 | 3.302 | 4.147 | 2.912 |
| Pbr013129.1 | Alpha-expansin 8 precursor, putative | 0.618 | 2.671 | 3.52 | 4.049 | 2.477 | 9.597 | 6.585 |
| Pbr013752.1 | hypothetical protein LOC100243043 | 8.278 | 6.678 | 0.282 | 18.801 | 0.413 | 0.474 | 2.659 |
| Pbr014568.1 | Major pollen allergen Ory s 1 precursor, putative | 5.807 | 1.336 | 2.112 | 5.785 | 1.926 | 0.355 | 1.773 |
| Pbr015713.1 | hypothetical protein LOC100264455 | 0.247 | 1.736 | 1.69 | 2.314 | 3.302 | 4.147 | 2.912 |
| Pbr015714.1 | hypothetical protein LOC100264455 | 0.247 | 0.668 | 0.704 | 1.543 | 1.789 | 2.488 | 2.279 |
| Pbr017015.1 | hypothetical protein LOC100255960 | 4.818 | 0.935 | 0.141 | 1.157 | 0.413 | 0.355 | 0.127 |
| Pbr017279.1 | Alpha-expansin 8 precursor, putative | 2.595 | 9.884 | 16.05 | 7.231 | 9.356 | 40.403 | 24.059 |
| Pbr019282.1 | Alpha-expansin 5 precursor, putative | 50.532 | 228.399 | 23.512 | 51.775 | 78.837 | 75.356 | 35.329 |
| Pbr020785.1 | Alpha-expansin 4 precursor, putative | 7.907 | 14.959 | 4.928 | 13.016 | 43.202 | 7.346 | 5.572 |
| Pbr022160.1 | Major pollen allergen Ory s 1 precursor, putative | 0.494 | 0.534 | 0.141 | 1.543 | 0 | 0.118 | 0.507 |
| Pbr025427.1 | hypothetical protein LOC100259398 | 10.378 | 5.61 | 3.801 | 6.556 | 1.238 | 1.422 | 1.52 |
| Pbr030256.1 | Major pollen allergen Ory s 1 precursor, putative | 5.93 | 1.336 | 2.112 | 5.978 | 1.926 | 0.355 | 1.773 |
| Pbr032622.1 | Beta-expansin 3 precursor, putative | 1.235 | 5.61 | 0.986 | 4.146 | 11.832 | 4.858 | 1.14 |
| Pbr033313.1 | Beta-expansin 1a precursor, putative | 163.827 | 69.856 | 189.365 | 93.136 | 147.493 | 139.93 | 167.783 |
| Pbr033539.1 | PtEXPA16; hypothetical protein | 119.472 | 185.524 | 57.865 | 83.109 | 287.557 | 131.044 | 118.144 |
| Pbr034745.1 | Beta-expansin 3 precursor, putative | 2.224 | 7.48 | 3.238 | 3.278 | 12.933 | 7.702 | 1.266 |
| Pbr035527.1 | PtrEXPA21; hypothetical protein | 2.595 | 5.209 | 2.112 | 4.049 | 13.071 | 5.332 | 5.952 |
| Pbr038442.1 | hypothetical protein LOC100244103 | 18.532 | 88.688 | 9.433 | 22.079 | 35.497 | 29.503 | 14.816 |
| Pbr039073.1 | PtEXPA4; hypothetical protein | 112.677 | 202.621 | 55.19 | 41.362 | 153.685 | 124.053 | 105.481 |
| Pbr000187.1 | Xyloglucan endotransglucosylase/hydrolase protein A precursor, putative (EC:2.4.1.207); K08235 xyloglucan:xyloglucosyl transferase [EC:2.4.1.207] | 1.483 | 2.805 | 1.267 | 2.121 | 5.091 | 0.237 | 0.253 |
| Pbr002336.1 | hypothetical protein; K08235 xyloglucan:xyloglucosyl transferase [EC:2.4.1.207] | 72.4 | 18.032 | 17.881 | 43.483 | 4.816 | 1.54 | 3.166 |
| Pbr005295.1 | hypothetical protein LOC100255547; K08235 xyloglucan:xyloglucosyl transferase [EC:2.4.1.207] | 2.224 | 7.48 | 0.141 | 0.386 | 3.715 | 1.659 | 2.659 |
| Pbr005296.1 | hypothetical protein LOC100255547; K08235 xyloglucan:xyloglucosyl transferase [EC:2.4.1.207] | 4.448 | 12.021 | 0.141 | 0.964 | 7.292 | 3.436 | 4.305 |
| Pbr015488.1 | hypothetical protein LOC100250908; K08235 xyloglucan:xyloglucosyl transferase [EC:2.4.1.207] | 0.247 | 1.87 | 1.267 | 0.386 | 1.651 | 2.37 | 1.013 |
| Pbr016300.1 | hypothetical protein; K08235 xyloglucan:xyloglucosyl transferase | 219.918 | 62.109 | 52.234 | 128.521 | 14.722 | 4.265 | 9.117 |
| Pbr018052.1 | hypothetical protein; K08235 xyloglucan:xyloglucosyl transferase [EC:2.4.1.207] | 416.485 | 1086.166 | 1102.823 | 845.074 | 1589.679 | 1849.19 | 1865.235 |
| Pbr018608.1 | hypothetical protein; K08235 xyloglucan:xyloglucosyl transferase [EC:2.4.1.207] | 101.311 | 253.51 | 101.511 | 70.093 | 236.374 | 211.732 | 180.952 |
| Pbr025659.1 | hypothetical protein LOC100261688; K08235 xyloglucan:xyloglucosyl transferase [EC:2.4.1.207] | 0.247 | 0 | 1.126 | 1.832 | 0.826 | 0.237 | 0.38 |
| Pbr028398.1 | hypothetical protein; K08235 xyloglucan:xyloglucosyl transferase [EC:2.4.1.207] | 83.519 | 206.762 | 188.52 | 153.492 | 304.892 | 319.909 | 292.511 |
| Pbr029887.1 | hypothetical protein; K08235 xyloglucan:xyloglucosyl transferase [EC:2.4.1.207] | 10.502 | 19.234 | 6.054 | 15.812 | 3.852 | 6.635 | 9.37 |
| Pbr032465.1 | hypothetical protein LOC100252949; K08235 xyloglucan:xyloglucosyl transferase [EC:2.4.1.207] | 8.031 | 4.141 | 2.816 | 1.253 | 2.477 | 0.474 | 0.76 |
| Pbr032469.1 | hypothetical protein LOC100252949; K08235 xyloglucan:xyloglucosyl transferase [EC:2.4.1.207] | 8.031 | 4.141 | 2.816 | 1.253 | 2.477 | 0.474 | 0.76 |
| Pbr032471.1 | hypothetical protein; K14504 xyloglucan:xyloglucosyl transferase TCH4 [EC:2.4.1.207] | 4.201 | 3.606 | 1.69 | 0.771 | 0.55 | 0.948 | 1.646 |
| Pbr032472.1 | hypothetical protein; K14504 xyloglucan:xyloglucosyl transferase TCH4 [EC:2.4.1.207] | 4.201 | 3.606 | 1.69 | 0.771 | 0.55 | 0.948 | 1.646 |
| Pbr032474.1 | hypothetical protein LOC100252949; K08235 xyloglucan:xyloglucosyl transferase [EC:2.4.1.207] | 8.031 | 4.141 | 2.816 | 1.253 | 2.477 | 0.474 | 0.76 |
| Pbr033336.1 | hypothetical protein; K08235 xyloglucan:xyloglucosyl transferase [EC:2.4.1.207] | 417.844 | 1109.006 | 1104.794 | 863.103 | 1617.196 | 1857.01 | 1884.989 |
| Pbr037164.1 | hypothetical protein; K08235 xyloglucan:xyloglucosyl transferase [EC:2.4.1.207] | 73.759 | 19.1 | 19.007 | 43.965 | 5.091 | 1.54 | 3.166 |
| Pbr037672.1 | hypothetical protein; K14504 xyloglucan:xyloglucosyl transferase TCH4 [EC:2.4.1.207] | 4.201 | 3.606 | 1.69 | 0.771 | 0.55 | 0.948 | 1.646 |
| Pbr037814.1 | hypothetical protein; K08235 xyloglucan:xyloglucosyl transferase [EC:2.4.1.207] | 170.745 | 87.753 | 55.19 | 98.825 | 82.139 | 72.868 | 72.052 |
| Pbr041156.1 | hypothetical protein LOC100261688; K08235 xyloglucan:xyloglucosyl transferase [EC:2.4.1.207] | 0.371 | 0 | 1.408 | 1.928 | 1.101 | 0.237 | 0.507 |
| Pbr042842.1 | Xyloglucan endotransglucosylase/hydrolase protein A precursor, putative (EC:2.4.1.207); K08235 xyloglucan:xyloglucosyl transferase [EC:2.4.1.207] | 6.177 | 6.278 | 2.534 | 4.339 | 11.007 | 1.303 | 0.886 |
| Pbr010451.1 | Pectinesterase-2 precursor, putative (EC:3.1.1.11); K01051 pectinesterase [EC:3.1.1.11] | 7.166 | 14.826 | 5.35 | 2.025 | 6.742 | 8.057 | 8.611 |
| Pbr011701.1 | Pectinesterase PPE8B precursor, putative (EC:3.1.1.11) | 0.124 | 0.534 | 0.704 | 1.061 | 2.477 | 2.251 | 1.52 |
| Pbr011702.1 | pectinesterase family protein (EC:3.1.1.11); K01051 pectinesterase [EC:3.1.1.11] | 0.865 | 2.538 | 0 | 0.964 | 5.916 | 0.829 | 0.253 |
| Pbr012442.1 | hypothetical protein LOC100255703; K01051 pectinesterase [EC:3.1.1.11] | 0.124 | 0 | 0 | 1.061 | 0.275 | 0 | 0 |
| Pbr013577.1 | pectinesterase family protein (EC:3.1.1.11) | 53.62 | 120.344 | 103.06 | 62.573 | 192.071 | 153.201 | 71.672 |
| Pbr014900.1 | Pectinesterase precursor, putative (EC:3.1.1.11) | 6.177 | 2.538 | 1.408 | 0 | 0.275 | 0.118 | 0 |
| Pbr015033.1 | pectinesterase family protein (EC:3.1.1.11) | 1.359 | 0 | 0.141 | 0.289 | 0 | 0.474 | 0.127 |
| Pbr015536.1 | Pectinesterase PPE8B precursor, putative (EC:3.1.1.11); K01051 pectinesterase [EC:3.1.1.11] | 0.247 | 1.469 | 1.126 | 0.578 | 1.376 | 2.844 | 0.38 |
| Pbr018272.1 | pectinesterase family protein (EC:3.1.1.11) | 0.371 | 3.072 | 0.422 | 1.157 | 4.678 | 1.422 | 0 |
| Pbr022717.1 | pectinesterase family protein (EC:3.1.1.11) | 2.224 | 0.267 | 0 | 1.446 | 0.275 | 0 | 0 |
| Pbr022719.1 | Pectinesterase-2 precursor, putative (EC:3.1.1.11); K01051 pectinesterase [EC:3.1.1.11] | 9.019 | 2.137 | 0.282 | 2.41 | 1.651 | 0 | 0 |
| Pbr022732.1 | pectinesterase family protein (EC:3.1.1.11) | 24.216 | 52.893 | 96.724 | 34.131 | 35.497 | 53.911 | 61.541 |
| Pbr024742.1 | Pectinesterase-2 precursor, putative (EC:3.1.1.11); K01051 pectinesterase [EC:3.1.1.11] | 1.483 | 3.473 | 0.422 | 0.868 | 2.064 | 0.829 | 2.026 |
| Pbr026333.1 | Pectinesterase-2 precursor, putative (EC:3.1.1.11); K01051 pectinesterase [EC:3.1.1.11] | 17.05 | 5.209 | 0.141 | 3.953 | 5.916 | 0.474 | 0.38 |
| Pbr026334.1 | Pectinesterase-2 precursor, putative (EC:3.1.1.11); K01051 pectinesterase [EC:3.1.1.11] | 2.718 | 0.267 | 0 | 2.025 | 0.55 | 0 | 0 |
| Pbr026344.1 | Pectinesterase-2 precursor, putative (EC:3.1.1.11); K01051 pectinesterase [EC:3.1.1.11] | 2.718 | 0.267 | 0 | 2.025 | 0.55 | 0 | 0 |
| Pbr026345.1 | Pectinesterase-2 precursor, putative (EC:3.1.1.11); K01051 pectinesterase [EC:3.1.1.11] | 18.038 | 5.476 | 0.141 | 4.049 | 6.054 | 0.355 | 0.507 |
| Pbr028958.1 | pectinesterase family protein (EC:3.1.1.11) | 2.842 | 2.137 | 9.574 | 7.328 | 6.742 | 2.251 | 2.533 |
| Pbr030314.1 | hypothetical protein LOC100255703; K01051 pectinesterase [EC:3.1.1.11] | 0.124 | 0 | 0 | 1.061 | 0.275 | 0 | 0 |
| Pbr030942.1 | Pectinesterase U1 precursor, putative (EC:3.1.1.11) | 0.371 | 0.134 | 1.69 | 1.157 | 5.091 | 1.185 | 1.52 |
| Pbr031522.1 | pectinesterase family protein (EC:3.1.1.11) | 28.169 | 61.574 | 109.536 | 38.566 | 43.065 | 61.494 | 69.392 |
| Pbr037378.1 | pectinesterase family protein (EC:3.1.1.11); K01051 pectinesterase [EC:3.1.1.11] | 9.143 | 32.724 | 2.253 | 6.749 | 43.34 | 9.36 | 1.899 |
| Pbr037379.1 | Pectinesterase PPE8B precursor, putative (EC:3.1.1.11) | 5.436 | 20.169 | 5.069 | 8.388 | 32.333 | 14.455 | 5.318 |
| Pbr005280.1 | Pectate lyase precursor, putative (EC:4.2.2.2); K01728 pectate lyase [EC:4.2.2.2] | 5.807 | 0.935 | 1.549 | 17.547 | 9.493 | 1.54 | 0.633 |
| Pbr010726.1 | hypothetical protein; K01728 pectate lyase [EC:4.2.2.2] | 15.691 | 40.204 | 18.021 | 27.092 | 65.079 | 20.142 | 17.095 |
| Pbr010727.1 | hypothetical protein LOC100246124; K01728 pectate lyase [EC:4.2.2.2] | 10.749 | 25.645 | 8.025 | 15.234 | 34.809 | 13.626 | 8.484 |
| Pbr010756.1 | Pectate lyase precursor, putative (EC:4.2.2.2); K01728 pectate lyase [EC:4.2.2.2] | 31.011 | 74.797 | 29.989 | 48.111 | 115.16 | 38.626 | 30.897 |
| Pbr011950.1 | hypothetical protein; K01728 pectate lyase [EC:4.2.2.2] | 26.934 | 38.334 | 18.303 | 34.709 | 65.904 | 13.626 | 10.89 |
| Pbr020778.1 | Pectate lyase precursor, putative (EC:4.2.2.2); K01728 pectate lyase [EC:4.2.2.2] | 2.1 | 14.826 | 1.408 | 8.292 | 37.974 | 1.896 | 0.633 |
| Pbr020977.1 | hypothetical protein; K01728 pectate lyase [EC:4.2.2.2] | 6.548 | 10.952 | 6.195 | 6.171 | 17.198 | 10.545 | 12.03 |
| Pbr021157.1 | hypothetical protein; K01728 pectate lyase [EC:4.2.2.2] | 19.15 | 15.494 | 15.206 | 22.657 | 30.682 | 9.36 | 6.965 |
| Pbr024016.1 | hypothetical protein; K01728 pectate lyase [EC:4.2.2.2] | 99.952 | 31.789 | 42.097 | 188.587 | 81.176 | 115.878 | 45.333 |
| Pbr026466.1 | hypothetical protein LOC100246761; K01728 pectate lyase [EC:4.2.2.2] | 10.996 | 13.49 | 12.812 | 10.606 | 8.255 | 11.967 | 18.108 |
| Pbr032243.1 | hypothetical protein; K01728 pectate lyase [EC:4.2.2.2] | 15.444 | 12.422 | 8.307 | 17.933 | 32.608 | 11.73 | 14.562 |
| Pbr032986.2 | Pectate lyase precursor, putative (EC:4.2.2.2); K01728 pectate lyase [EC:4.2.2.2] | 3.336 | 1.069 | 3.379 | 9.738 | 12.658 | 7.465 | 2.406 |
| Pbr035229.1 | hypothetical protein LOC100262177; K01728 pectate lyase [EC:4.2.2.2] | 3.583 | 1.336 | 0.563 | 0.096 | 0.138 | 0 | 0 |
| Pbr035305.1 | Pectate lyase precursor, putative (EC:4.2.2.2); K01728 pectate lyase [EC:4.2.2.2] | 3.336 | 5.743 | 2.112 | 5.014 | 20.776 | 1.54 | 1.52 |
| Pbr036221.1 | Pectate lyase precursor, putative (EC:4.2.2.2) | 13.714 | 30.72 | 2.534 | 10.22 | 20.225 | 3.792 | 1.646 |
| Pbr036977.1 | hypothetical protein; K01728 pectate lyase [EC:4.2.2.2] | 49.296 | 34.327 | 37.31 | 129.581 | 73.334 | 111.968 | 33.936 |
| Pbr039133.1 | Pectate lyase precursor, putative (EC:4.2.2.2); K01728 pectate lyase [EC:4.2.2.2] | 10.378 | 4.007 | 1.83 | 0.289 | 0.275 | 0.237 | 0.38 |
| Pbr041275.2 | hypothetical protein; K01728 pectate lyase [EC:4.2.2.2] | 85.743 | 65.715 | 50.685 | 97.282 | 120.801 | 33.294 | 26.339 |
| Pbr042465.1 | hypothetical protein; K01728 pectate lyase [EC:4.2.2.2] | 5.56 | 8.949 | 6.758 | 8.484 | 17.474 | 9.716 | 8.737 |
| Pbr010853.1 | polygalacturonase, putative (EC:3.2.1.15) | 45.343 | 14.959 | 18.162 | 119.747 | 9.906 | 7.109 | 6.585 |
| Pbr017969.1 | Polygalacturonase precursor, putative (EC:3.2.1.67); K01184 polygalacturonase [EC:3.2.1.15] | 0 | 0.801 | 0 | 2.603 | 2.339 | 0.829 | 0.127 |
| Pbr018658.1 | polygalacturonase, putative (EC:3.2.1.15) | 8.401 | 2.671 | 3.379 | 19.765 | 1.926 | 1.066 | 0.76 |
| Pbr021519.1 | polygalacturonase, putative (EC:3.2.1.15) | 4.571 | 2.271 | 1.83 | 10.22 | 0.826 | 0.829 | 0.127 |
| Pbr021568.1 | polygalacturonase, putative (EC:3.2.1.15) | 4.571 | 2.271 | 1.83 | 10.22 | 0.826 | 0.829 | 0.127 |
| Pbr027032.1 | Polygalacturonase precursor, putative (EC:3.2.1.67); K01184 polygalacturonase [EC:3.2.1.15] | 0 | 0.801 | 0 | 2.603 | 2.339 | 0.829 | 0.127 |
| Pbr027731.1 | polygalacturonase, putative (EC:3.2.1.15) | 27.922 | 36.197 | 128.684 | 50.425 | 45.541 | 122.039 | 74.457 |
| Pbr036382.1 | Polygalacturonase precursor, putative (EC:3.2.1.67); K01184 polygalacturonase [EC:3.2.1.15] | 17.668 | 1.603 | 0.282 | 25.839 | 2.064 | 0.474 | 0.127 |
|  |  |  |  |  |  |  |  |  |
| Transcription factors | | | | | | | | |
| Gene ID | Gene Description | TPM (transcripts per million clean tag)  C1 C2 C3 C4 C5 C6 C7 | | | | | | |
| Pbr000268.1 | hypothetical protein LOC100256820; | 35.459 | 12.822 | 5.772 | 8.87 | 13.071 | 2.133 | 5.065 |
| Pbr000351.1 | tfiih, polypeptide, putative | 4.448 | 9.75 | 7.603 | 5.881 | 9.906 | 10.427 | 7.091 |
| Pbr000753.1 | DNA binding protein, putative; K09286 EREBP-like factor | 2.1 | 2.137 | 3.097 | 5.399 | 0.688 | 1.185 | 1.14 |
| Pbr000874.1 | hypothetical protein LOC100260239 | 9.143 | 6.678 | 7.18 | 8.581 | 11.695 | 5.332 | 5.318 |
| Pbr000876.1 | r2r3-myb transcription factor | 24.833 | 5.61 | 8.729 | 20.44 | 4.678 | 5.095 | 3.419 |

| Pbr000980.1 | Protein AINTEGUMENTA, putative (EC:1.3.1.74); K09284 AP2-like factor, euAP2 lineage | | 6.919 | | 3.379 | | 2.41 | 4.403 | | | 1.185 | | 0.76 | | 0.76 |
| --- | --- | --- | --- | --- | --- | --- | --- | --- | --- | --- | --- | --- | --- | --- | --- |
| Pbr001520.1 | hypothetical protein LOC100256820; K09422 myb proto-oncogene protein, plant | | 97.11 | | 7.884 | | 22.85 | 8.987 | | | 2.607 | | 5.825 | | 5.825 |
| Pbr001638.1 | MYB030; hypothetical protein; K09422 myb proto-oncogene | | 0.494 | | 3.379 | | 0.675 | 9.631 | | | 1.303 | | 2.153 | | 2.153 |
| Pbr001709.1 | MYB210; hypothetical protein; K09422 myb proto-oncogene protein, plant | | 1.606 | | 0.845 | | 1.35 | 0 | | | 0 | | 0 | | 0 |
| Pbr001888.1 | MYB074; hypothetical protein; K09422 myb proto-oncogene protein, plant | | 3.706 | | 10.419 | | 6.749 | 6.879 | | | 14.692 | | 15.069 | | 15.069 |
| Pbr002006.1 | MYB052; hypothetical protein | | 0.247 | | 1.549 | | 2.314 | 8.943 | | | 2.725 | | 1.393 | | 1.393 |
| Pbr002014.1 | MYB053; hypothetical protein; K09422 myb proto-oncogene protein, plant | | 1.606 | | 0.986 | | 0.964 | 0.413 | | | 0.237 | | 1.52 | | 1.52 |
| Pbr002230.1 | hypothetical protein LOC100251862; K13426 WRKY transcription factor 29 | | 36.818 | | 0.422 | | 11.955 | 6.329 | | | 0.474 | | 1.52 | | 1.52 |
| Pbr002239.1 | r2r3-myb transcription factor, putative; K09422 myb proto-oncogene protein, plant | | 0 | | 5.491 | | 2.892 | 0.275 | | | 5.213 | | 4.685 | | 4.685 |
| Pbr002338.1 | hypothetical protein LOC100243644; K09060 plant G-box-binding factor | | 14.702 | | 10.137 | | 11.666 | 14.859 | | | 7.583 | | 7.344 | | 7.344 |
| Pbr002427.2 | MADS1; MAD-box transcripion factor; K09264 MADS-box transcription factor, plant | | 1.112 | | 5.632 | | 4.531 | 2.614 | | | 4.976 | | 2.786 | | 2.786 |
| Pbr002460.1 | hypothetical protein LOC100252531; K09286 EREBP-like factor | | 0.371 | | 1.603 | | 0.141 | 0.193 | | | 1.238 | | 0.118 | | 0 |
| Pbr003370.1 | MYB5b; K09422 myb proto-oncogene protein, plant | | 14.702 | | 11.487 | | 5.772 | 8.002 | | | 9.218 | | 6.872 | | 9.624 |
| Pbr003518.1 | hypothetical protein; K14431 transcription factor TGA | | 0.988 | | 0 | | 2.675 | 0.675 | | | 0.138 | | 0 | | 1.14 |
| Pbr003636.1 | hypothetical protein LOC100256589; K09285 AP2-like factor | | 4.695 | | 0.267 | | 1.549 | 2.218 | | | 2.339 | | 0.711 | | 0.76 |
| Pbr003659.1 | MYB144; hypothetical protein; K09422 myb proto-oncogene protein, plant | | 0.988 | | 0.935 | | 2.675 | 3.375 | | | 0 | | 0 | | 0.127 |
| Pbr003927.1 | hypothetical protein LOC100262843; K09287 RAV-like factor | | 0 | | 0.401 | | 0.141 | 2.218 | | | 6.329 | | 0 | | 0.253 |
| Pbr003928.1 | hypothetical protein LOC100262843; K09287 RAV-like factor | | 0.124 | | 0 | | 0.141 | 0.289 | | | 2.614 | | 0 | | 0.127 |
| Pbr004276.1 | MYB168; hypothetical protein; K09422 myb proto-oncogene protein, plant | | 9.76 | | 3.206 | | 1.69 | 3.953 | | | 4.816 | | 2.725 | | 1.646 |
| Pbr004690.1 | hypothetical protein LOC100256589; K09285 AP2-like factor, ANT lineage | | 0.371 | | 0.401 | | 0.422 | 0.675 | | | 1.789 | | 0.237 | | 0.127 |
| Pbr004921.1 | MYB003; hypothetical protein; K09422 myb proto-oncogene protein, plant | | 0.124 | | 0.534 | | 3.52 | 2.025 | | | 1.651 | | 4.976 | | 3.672 |
| Pbr005213.1 | hypothetical protein LOC100263756; K03131 transcription initiation factor TFIID subunit 6 | | 9.266 | | 19.901 | | 9.292 | 9.352 | | | 15.272 | | 12.559 | | 11.017 |
| Pbr005982.2 | MYB170; hypothetical protein; K09422 myb proto-oncogene | | 4.695 | | 9.617 | | 9.855 | 5.592 | | | 9.493 | | 9.597 | | 16.588 |
| Pbr006046.1 | hypothetical protein LOC100248302; K09060 plant G-box-binding factor | | 10.131 | | 5.61 | | 7.462 | 4.242 | | | 6.054 | | 1.659 | | 2.912 |
| Pbr006264.1 | hypothetical protein LOC100256181; K09422 myb proto-oncogene protein, plant | | 6.177 | | 4.942 | | 11.404 | 4.242 | | | 3.99 | | 3.91 | | 5.698 |
| Pbr006271.1 | hypothetical protein LOC100256209; K09422 myb proto-oncogene protein, plant | | 4.324 | | 11.353 | | 2.112 | 3.567 | | | 12.52 | | 2.014 | | 4.559 |
| Pbr006380.1 | hypothetical protein LOC100256849; K13425 WRKY transcription factor 22 | | 2.965 | | 3.873 | | 1.408 | 2.121 | | | 2.339 | | 0.118 | | 0.886 |
| Pbr006434.1 | transcription initiation factor iib, putative; K03124 transcription initiation factor TFIIB | | 18.162 | | 5.343 | | 11.545 | 4.049 | | | 3.99 | | 9.005 | | 9.117 |
| Pbr006435.1 | transcription initiation factor iib, putative; K03124 transcription initiation factor TFIIB | | 18.162 | | 5.343 | | 11.545 | 4.049 | | | 3.99 | | 9.005 | | 9.117 |
| Pbr006499.1 | transcription initiation factor iib, putative; K03124 transcription initiation factor TFIIB | | 17.544 | | 5.343 | | 10.841 | 3.953 | | | 3.99 | | 8.649 | | 8.611 |
| Pbr006626.1 | MYB167; hypothetical protein; K09422 myb proto-oncogene protein, plant | | 3.089 | | 0.134 | | 3.238 | 1.639 | | | 1.651 | | 2.725 | | 2.786 |
| Pbr006685.1 | MYB170; hypothetical protein; K09422 myb proto-oncogene protein, plant | | 15.691 | | 26.046 | | 33.227 | 18.319 | | | 20.5 | | 31.043 | | 54.197 |
| Pbr006814.1 | hypothetical protein LOC100263039; K07466 replication factor A1 | | 5.313 | | 5.476 | | 11.686 | 8.484 | | | 5.366 | | 7.82 | | 10.51 |
| Pbr006862.1 | hypothetical protein LOC100252531; K09286 EREBP-like factor | | 0.124 | | 0.935 | | 0 | 0.096 | | | 1.101 | | 0.118 | | 0 |
| Pbr007127.1 | hypothetical protein; K03128 transcription initiation factor TFIID subunit 2 | | 2.1 | | 3.206 | | 2.393 | 3.182 | | | 4.128 | | 1.066 | | 0.507 |
| Pbr007180.1 | mads box protein, putative (EC:1.3.1.74) | | 55.35 | | 83.746 | | 77.999 | 33.167 | | | 42.927 | | 45.617 | | 56.223 |
| Pbr007206.1 | hypothetical protein LOC100255412; K07466 replication factor A1 | | 0.741 | | 0.801 | | 0.141 | 2.121 | | | 3.302 | | 0.948 | | 0.633 |
| Pbr007589.1 | hypothetical protein; K14432 ABA responsive element binding factor | | 1.977 | | 3.873 | | 4.787 | 2.507 | | | 2.614 | | 6.28 | | 5.952 |
| Pbr007949.1 | hypothetical protein LOC100256849; K13425 WRKY transcription factor 22 | | 3.954 | | 4.141 | | 1.408 | 2.314 | | | 3.027 | | 0.237 | | 0.886 |
| Pbr007956.1 | hypothetical protein LOC100256849; K13425 WRKY transcription factor 22 | | 3.954 | | 4.141 | | 1.408 | 2.314 | | | 3.027 | | 0.237 | | 0.886 |
| Pbr008557.1 | hypothetical protein LOC100255706; K14431 transcription factor TGA | | 42.13 | | 10.418 | | 19.992 | 29.792 | | | 1.376 | | 0.474 | | 1.14 |
| Pbr008630.1 | MYB210; hypothetical protein; K09422 myb proto-oncogene protein, plant | | 14.332 | | 5.476 | | 6.054 | 7.617 | | | 0.55 | | 0 | | 0.253 |
| Pbr008796.1 | DREB81; AP2/ERF domain-containing transcription factor; K09286 EREBP-like factor | | 0.988 | | 1.069 | | 0.704 | 2.892 | | | 2.339 | | 2.133 | | 0.253 |
| Pbr009772.1 | hypothetical protein LOC100254115; K03125 transcription initiation factor TFIID subunit 1 | | 30.393 | | 33.125 | | 38.155 | 36.541 | | | 35.635 | | 21.09 | | 14.942 |
| Pbr009778.1 | hypothetical protein LOC100254115; K03125 transcription initiation factor TFIID subunit 1 | | 30.393 | | 33.125 | | 38.155 | 36.541 | | | 35.635 | | 21.09 | | 14.942 |
| Pbr009823.1 | MYB30; R2R3 Myb30 transcription factor; K09422 myb proto-oncogene protein, plant | | 3.706 | | 9.35 | | 4.224 | 1.543 | | | 4.678 | | 5.806 | | 7.724 |
| Pbr010210.1 | MYB008; hypothetical protein; K09422 myb proto-oncogene protein, plant | | 0.247 | | 0.267 | | 0.422 | 0.096 | | | 1.651 | | 0.592 | | 1.013 |
| Pbr010445.1 | hypothetical protein LOC100250496; K14514 ethylene-insensitive protein 3 | | 7.166 | | 12.288 | | 17.74 | 12.052 | | | 11.42 | | 10.545 | | 14.436 |
| Pbr010448.1 | hypothetical protein LOC100250496; K14514 ethylene-insensitive protein 3 | | 7.166 | | 12.288 | | 17.74 | 12.052 | | | 11.42 | | 10.545 | | 14.436 |
| Pbr011095.1 | MYB220; hypothetical protein; K09422 myb proto-oncogene protein, plant | | 3.459 | | 1.736 | | 0.141 | 1.928 | | | 5.366 | | 0.237 | | 0.127 |
| Pbr011145.1 | DNA binding protein, putative; K09286 EREBP-like factor | | 242.157 | | 322.163 | | 107.706 | 184.827 | | | 151.208 | | 101.067 | | 102.949 |
| Pbr011262.1 | MYB049; hypothetical protein; K09422 myb proto-oncogene protein, plant | | 1.359 | | 6.144 | | 0 | 0.675 | | | 3.302 | | 0.711 | | 1.013 |
| Pbr011268.1 | r2r3-myb transcription factor, putative; K09422 myb proto-oncogene protein, plant | | 0.618 | | 0.668 | | 0.704 | 0.482 | | | 4.128 | | 0 | | 0.76 |
| Pbr011369.1 | hypothetical protein LOC100240975; K09422 myb proto-oncogene protein, plant | | 1.73 | | 3.339 | | 1.126 | 0.964 | | | 3.577 | | 1.777 | | 2.026 |
| Pbr011515.1 | hypothetical protein; K03124 transcription initiation factor TFIIB | | 0 | | 1.202 | | 0.563 | 0.289 | | | 0.413 | | 0.355 | | 0.127 |
| Pbr012624.1 | MYB018; hypothetical protein; K09422 myb proto-oncogene protein, plant | | 4.201 | | 7.88 | | 6.476 | 6.653 | | | 11.282 | | 11.256 | | 12.663 |
| Pbr012750.1 | MYB199, MYB200; hypothetical protein | | 0.371 | | 0.801 | | 24.075 | 3.278 | | | 1.376 | | 23.578 | | 24.693 |
| Pbr012968.1 | MYB032; hypothetical protein; K09422 myb proto-oncogene protein, plant | | 0 | | 0.134 | | 0.704 | 0 | | | 1.651 | | 0.118 | | 0.633 |
| Pbr013092.1 | similar to DNA-binding protein; K13424 WRKY transcription factor 33 | | 141.341 | | 116.203 | | 117.561 | 90.533 | | | 67.28 | | 60.427 | | 90.919 |
| Pbr013149.1 | hypothetical protein LOC100246641; K09286 EREBP-like factor | | 38.671 | | 35.128 | | 24.639 | 34.806 | | | 76.361 | | 36.493 | | 42.041 |
| Pbr013255.1 | DNA-binding protein RAV1, putative; K09287 RAV-like factor | | 108.476 | | 139.444 | | 377.041 | 265.526 | | | 207.756 | | 269.079 | | 594.773 |
| Pbr013267.1 | Transcription factor HBP-1b(c1), putative; K14431 transcription factor TGA | | 8.154 | | 5.209 | | 4.646 | 5.014 | | | 4.128 | | 1.303 | | 2.659 |
| Pbr013315.1 | MYB034; hypothetical protein; K09422 myb proto-oncogene protein, plant | | 56.956 | | 22.039 | | 24.639 | 44.544 | | | 24.078 | | 9.834 | | 19.501 |
| Pbr013714.1 | hypothetical protein LOC100262843; K09287 RAV-like factor | | 0.124 | | 0 | | 0.141 | 0.289 | | | 2.614 | | 0 | | 0.127 |
| Pbr013715.1 | hypothetical protein LOC100262843; K09287 RAV-like factor | | 0 | | 0.401 | | 0.141 | 2.218 | | | 6.329 | | 0 | | 0.253 |
| Pbr013860.1 | MYB130; hypothetical protein; K09422 myb proto-oncogene protein, plant | | 0.494 | | 0.134 | | 0 | 0.096 | | | 1.101 | | 0.118 | | 0.253 |
| Pbr014161.1 | MYB144; hypothetical protein; K09422 myb proto-oncogene protein, plant | | 0.988 | | 0.935 | | 2.816 | 3.182 | | | 0 | | 0 | | 0.127 |
| Pbr014186.2 | Protein AINTEGUMENTA, putative (EC:1.3.1.74); K09284 AP2-like factor, euAP2 lineage | | 6.919 | | 3.606 | | 3.379 | 2.41 | | | 4.403 | | 1.185 | | 0.76 |
| Pbr014381.1 | hypothetical protein LOC100251783; K09422 myb proto-oncogene protein, plant | | 3.83 | | 5.076 | | 0.704 | 2.796 | | | 3.302 | | 0.948 | | 0.507 |
| Pbr014594.1 | hypothetical protein; K14431 transcription factor TGA | | 0.865 | | 0 | | 2.675 | 0.675 | | | 0.138 | | 0 | | 1.266 |
| Pbr014994.1 | MYB212; hypothetical protein; K09422 myb proto-oncogene protein, plant | | 28.787 | | 11.887 | | 8.448 | 27.96 | | | 12.796 | | 4.621 | | 7.724 |
| Pbr015230.1 | hypothetical protein LOC100244605; K09422 myb proto-oncogene protein, plant | | 4.818 | | 10.819 | | 1.971 | 5.206 | | | 14.997 | | 4.858 | | 7.851 |
| Pbr015263.1 | transcription factor, putative; K09422 myb proto-oncogene protein, plant | | 4.695 | | 11.22 | | 6.195 | 7.135 | | | 9.081 | | 5.095 | | 6.331 |
| Pbr015291.2 | AP2; transcription factor APETALA2; K09284 AP2-like factor, euAP2 lineage | | 57.45 | | 47.55 | | 36.043 | 43.579 | | | 25.591 | | 14.337 | | 19.754 |
| Pbr015293.1 | DREB33; AP2/ERF domain-containing transcription factor; K09286 EREBP-like factor | | 3.336 | | 4.408 | | 8.448 | 1.157 | | | 4.678 | | 4.858 | | 8.231 |
| Pbr015309.1 | SREBP; sucrose responsive element binding protein | | 117.743 | | 53.827 | | 134.597 | 129.485 | | | 95.898 | | 50.119 | | 73.191 |
| Pbr015502.1 | hypothetical protein LOC100252531; K09286 EREBP-like factor | | 50.161 | | 80.808 | | 0.845 | 7.906 | | | 15.135 | | 1.422 | | 4.939 |
| Pbr015763.1 | MYB199, MYB200; hypothetical protein; K09422 myb proto-oncogene protein, plant | | 0.371 | | 0.801 | | 22.949 | 3.471 | | | 1.513 | | 23.341 | | 24.693 |
| Pbr015939.1 | hypothetical protein; K13424 WRKY transcription factor 33 | | 241.292 | | 296.251 | | 74.901 | 104.995 | | | 102.502 | | 68.721 | | 93.958 |
| Pbr016049.2 | hypothetical protein LOC100245515; K09286 EREBP-like factor | | 262.79 | | 438.367 | | 230.476 | 239.687 | | | 331.172 | | 235.903 | | 146.636 |
| Pbr016302.1 | hypothetical protein LOC100243644; K09060 plant G-box-binding factor | | 22.115 | | 18.699 | | 15.628 | 17.451 | | | 15.41 | | 11.848 | | 9.497 |
| Pbr016404.1 | hypothetical protein LOC100260239; K09191 general transcription factor IIIA | | 9.39 | | 6.545 | | 7.321 | 8.87 | | | 11.97 | | 5.45 | | 5.445 |
| Pbr016625.1 | MYB018; hypothetical protein; K09422 myb proto-oncogene protein, plant | | 3.83 | | 6.011 | | 7.18 | 3.953 | | | 9.218 | | 6.161 | | 8.104 |
| Pbr016851.1 | MYB052; hypothetical protein; K09422 myb proto-oncogene protein, plant | | 0.618 | | 0.668 | | 2.957 | 4.242 | | | 10.044 | | 1.777 | | 0.38 |
| Pbr017262.1 | hypothetical protein LOC100258205; K09060 plant G-box-binding factor | | 16.556 | | 14.158 | | 21.4 | 18.415 | | | 16.098 | | 8.531 | | 13.802 |
| Pbr017297.1 | hypothetical protein LOC100246641; K09286 EREBP-like factor | | 24.833 | | 33.258 | | 12.53 | 25.55 | | | 62.602 | | 35.545 | | 24.819 |
| Pbr017533.1 | hypothetical protein LOC100244033; K09422 myb proto-oncogene protein, plant | | 1.73 | | 0.668 | | 5.35 | 3.76 | | | 7.292 | | 8.412 | | 6.458 |
| Pbr017791.1 | hypothetical protein LOC100242486; K03126 transcription initiation factor TFIID subunit 12 | | 3.954 | | 5.877 | | 3.801 | 4.531 | | | 10.594 | | 4.384 | | 4.939 |
| Pbr017813.1 | hypothetical protein LOC100243210; K09422 myb proto-oncogene protein, plant | | 0 | | 1.603 | | 19.57 | 13.691 | | | 5.641 | | 31.872 | | 17.981 |
| Pbr017966.1 | MYB24; R2R3 Myb24 transcription factor; K09422 myb proto-oncogene protein, plant | | 2.965 | | 1.87 | | 0 | 1.832 | | | 0.275 | | 0 | | 0.253 |
| Pbr018024.1 | hypothetical protein LOC100249492; K09422 myb proto-oncogene protein, plant | | 4.324 | | 0.935 | | 0.845 | 1.061 | | | 1.651 | | 0.355 | | 1.266 |
| Pbr018029.1 | MYB049; hypothetical protein; K09422 myb proto-oncogene protein, plant | | 1.853 | | 3.873 | | 0 | 0.771 | | | 1.238 | | 0.829 | | 0.507 |
| Pbr018952.1 | hypothetical protein LOC100241598; K03122 transcription initiation factor TFIIA large subunit | | 25.945 | | 25.912 | | 30.552 | 34.227 | | | 42.927 | | 19.55 | | 30.138 |
| Pbr019262.1 | hypothetical protein LOC100257755 | | 13.961 | | 30.186 | | 7.321 | 6.942 | | | 11.695 | | 7.346 | | 15.829 |
| Pbr019293.1 | MYB083; hypothetical protein | | 2.347 | | 7.613 | | 0.986 | 2.7 | | | 6.329 | | 4.147 | | 5.318 |
| Pbr019788.1 | hypothetical protein LOC100252316; K09285 AP2-like factor, ANT lineage | | 9.019 | | 19.367 | | 2.534 | 15.041 | | | 44.991 | | 4.976 | | 3.039 |
| Pbr019801.1 | hypothetical protein LOC100262671; K09422 myb proto-oncogene protein, plant | | 0.494 | | 0.935 | | 1.126 | 0.771 | | | 12.383 | | 1.303 | | 1.013 |
| Pbr019902.1 | hypothetical protein LOC100264614; K09422 myb proto-oncogene protein, plant | | 13.343 | | 2.271 | | 0.282 | 8.581 | | | 7.842 | | 0.829 | | 0.76 |
| Pbr019950.2 | hypothetical protein LOC100247660; K03134 transcription initiation factor TFIID subunit 10 | | 1.235 | | 3.74 | | 3.379 | 2.892 | | | 2.752 | | 2.133 | | 2.912 |
| Pbr020163.1 | hypothetical protein LOC100255412; K07466 replication factor A1 | | 0.371 | | 2.004 | | 0.563 | 2.121 | | | 5.091 | | 0.948 | | 0.507 |
| Pbr020189.1 | mads box protein, putative (EC:1.3.1.74); K09264 MADS-box transcription factor, plant | | 30.393 | | 36.063 | | 46.321 | 25.646 | | | 21.188 | | 19.195 | | 27.352 |
| Pbr020245.2 | hypothetical protein; K03128 transcription initiation factor | | 19.397 | | 23.107 | | 27.032 | 21.693 | | | 29.994 | | 15.758 | | 12.789 |
| Pbr020295.1 | hypothetical protein LOC100242243; K09422 myb proto-oncogene protein, plant | | 6.672 | | 2.938 | | 4.365 | 15.908 | | | 1.926 | | 0.237 | | 1.013 |
| Pbr020345.1 | hypothetical protein LOC100247566; K02202 cyclin-dependent kinase 7 [EC:2.7.11.22] | | 7.042 | | 7.213 | | 5.913 | 4.724 | | | 8.393 | | 2.962 | | 5.445 |
| Pbr020666.1 | hypothetical protein LOC100243210; K09422 myb proto-oncogene protein, plant | | 0.124 | | 0.668 | | 1.83 | 0.964 | | | 0.826 | | 1.659 | | 2.153 |
| Pbr020726.1 | r2r3-myb transcription factor, putative; K09422 myb proto-oncogene protein, plant | | 20.88 | | 13.49 | | 13.516 | 27.575 | | | 19.125 | | 7.82 | | 13.549 |
| Pbr020733.1 | r2r3-myb transcription factor, putative; K09422 myb proto-oncogene protein, plant | | 20.88 | | 13.49 | | 13.516 | 27.575 | | | 19.125 | | 7.82 | | 13.549 |
| Pbr020751.1 | hypothetical protein LOC100250017 | | 6.795 | | 2.671 | | 0.141 | 2.025 | | | 4.265 | | 0.237 | | 0.76 |
| Pbr021041.1 | hypothetical protein LOC100248302; K09060 plant G-box-binding factor | | 59.427 | | 24.71 | | 45.053 | 17.162 | | | 19.675 | | 14.811 | | 18.614 |
| Pbr021178.1 | MYB219; hypothetical protein; K09422 myb proto-oncogene protein, plant | | 12.973 | | 2.805 | | 0.563 | 18.319 | | | 3.44 | | 0.118 | | 0.127 |
| Pbr021193.1 | MYB219; hypothetical protein; K09422 myb proto-oncogene protein, plant | | 12.973 | | 2.805 | | 0.563 | 18.319 | | | 3.44 | | 0.118 | | 0.127 |
| Pbr022355.2 | hypothetical protein; K09250 cellular nucleic acid-binding protein | | 19.274 | | 29.919 | | 16.895 | 27.092 | | | 36.323 | | 25.474 | | 17.981 |
| Pbr022669.1 | MYB12, mybF1; R2R3 MYB 12 transcription factor; K09422 myb proto-oncogene protein, plant | | 1.235 | | 1.736 | | 0.282 | 1.832 | | | 4.265 | | 1.422 | | 1.14 |
| Pbr023044.1 | Ethylene-responsive transcription factor 1B, putative; K14516 ethylene-responsive transcription factor 1 | | 22.115 | | 18.299 | | 16.754 | 79.638 | | | 87.505 | | 25 | | 49.765 |
| Pbr023482.1 | hypothetical protein LOC100259031; K09422 myb proto-oncogene protein, plant | | 0.371 | | 0.267 | | 8.729 | 4.049 | | | 0.826 | | 8.412 | | 7.344 |
| Pbr023487.1 | hypothetical protein LOC100259031; K09422 myb proto-oncogene protein, plant | | 0.371 | | 0.267 | | 8.729 | 4.049 | | | 0.826 | | 8.412 | | 7.344 |
| Pbr023831.1 | MYB003; hypothetical protein; K09422 myb proto-oncogene protein, plant | | 0.247 | | 0.267 | | 2.253 | 0.964 | | | 1.376 | | 2.37 | | 1.393 |
| Pbr023902.1 | hypothetical protein LOC100259747; K09286 EREBP-like factor | | 112.306 | | 84.414 | | 53.783 | 94.583 | | | 45.541 | | 64.337 | | 110.42 |
| Pbr024078.1 | ERF82; AP2/ERF domain-containing transcription factor; K09286 EREBP-like factor | | 4.695 | | 5.877 | | 10.7 | 5.881 | | | 5.779 | | 8.886 | | 10.384 |
| Pbr024467.1 | Ethylene-responsive transcription factor, putative; K09286 EREBP-like factor | | 70.423 | | 59.17 | | 29.144 | 29.31 | | | 47.192 | | 22.868 | | 24.946 |
| Pbr024492.1 | MYB115; hypothetical protein; K09422 myb proto-oncogene protein, plant | | 0.865 | | 4.274 | | 3.379 | 2.314 | | | 5.779 | | 5.924 | | 3.925 |
| Pbr024739.1 | hypothetical protein LOC100250496 | | 332.224 | | 266.466 | | 426.599 | 375.342 | | | 303.104 | | 179.504 | | 341.896 |
| Pbr024978.1 | hypothetical protein LOC100264614; K09422 myb proto-oncogene protein, plant | | 14.332 | | 1.603 | | 0.141 | 2.7 | | | 4.678 | | 0.355 | | 0.253 |
| Pbr025174.1 | AP2; transcription factor APETALA2; K09284 AP2-like factor, euAP2 lineage | | 15.938 | | 35.262 | | 26.469 | 17.547 | | | 16.235 | | 12.322 | | 12.536 |
| Pbr025176.1 | DREB33; AP2/ERF domain-containing transcription factor; K09286 EREBP-like factor | | 2.347 | | 1.202 | | 6.054 | 1.35 | | | 1.651 | | 1.185 | | 1.899 |
| Pbr025199.1 | r2r3-myb transcription factor, putative; K09422 myb proto-oncogene protein, plant | | 62.392 | | 29.385 | | 81.519 | 62.38 | | | 58.337 | | 29.266 | | 45.333 |
| Pbr025360.1 | MYB190; hypothetical protein; K09422 myb proto-oncogene protein, plant | | 31.258 | | 22.306 | | 0.845 | 9.449 | | | 49.669 | | 1.066 | | 0.886 |
| Pbr025889.1 | CCA1; CCA1 (CIRCADIAN CLOCK ASSOCIATED 1); DNA binding / transcription activator/ transcription factor/ transcription repressor; K12134 circadian clock associated 1 | | 5.066 | | 3.072 | | 12.108 | 14.848 | | | 14.997 | | 2.37 | | 3.546 |
| Pbr026080.1 | MYB170; hypothetical protein; K09422 myb proto-oncogene | | 5.313 | | 9.216 | | 10.982 | 5.206 | | | 8.943 | | 9.479 | | 17.221 |
| Pbr026417.1 | MYB025; hypothetical protein; K09422 myb proto-oncogene protein, plant | | 2.965 | | 3.606 | | 1.971 | 4.724 | | | 4.953 | | 2.014 | | 2.533 |
| Pbr026913.1 | hypothetical protein LOC100249156; K14431 transcription factor TGA | | 12.602 | | 10.552 | | 11.827 | 19.283 | | | 8.806 | | 4.502 | | 7.218 |
| Pbr027035.1 | MYB24; R2R3 Myb24 transcription factor; K09422 myb proto-oncogene protein, plant | | 2.965 | | 1.87 | | 0 | 1.832 | | | 0.275 | | 0 | | 0.253 |
| Pbr027468.1 | hypothetical protein LOC100249239; K14431 transcription factor TGA | | 5.807 | | 2.938 | | 1.83 | 1.928 | | | 2.477 | | 0.237 | | 1.266 |
| Pbr027478.1 | hypothetical protein LOC100262843; K09287 RAV-like factor | | 158.885 | | 183.254 | | 300.028 | 257.33 | | | 228.669 | | 222.04 | | 502.841 |
| Pbr027596.1 | hypothetical protein LOC100260239; K09191 general transcription factor IIIA | | 6.177 | | 6.812 | | 6.899 | 7.52 | | | 5.641 | | 2.844 | | 3.799 |
| Pbr028210.1 | hypothetical protein LOC100251241; K09250 cellular nucleic acid-binding protein | | 12.231 | | 7.747 | | 4.365 | 8.292 | | | 3.164 | | 2.725 | | 4.559 |
| Pbr028319.1 | MYB167; hypothetical protein; K09422 myb proto-oncogene protein, plant | | 0.865 | | 0.267 | | 2.253 | 0.868 | | | 2.339 | | 2.133 | | 2.153 |
| Pbr028356.1 | hypothetical protein; K12236 transcriptional repressor NF-X1 [EC:6.3.2.-] | | 43.366 | | 48.084 | | 56.458 | 22.657 | | | 38.662 | | 30.569 | | 40.268 |
| Pbr028725.1 | MYB165; hypothetical protein; K09422 myb proto-oncogene protein, plant | | 2.471 | | 1.736 | | 1.83 | 5.881 | | | 2.889 | | 0.592 | | 3.546 |
| Pbr028812.1 | MYB164; hypothetical protein; K09422 myb proto-oncogene protein, plant | | 100.075 | | 26.179 | | 29.426 | 54.281 | | | 2.614 | | 0.829 | | 4.432 |
| Pbr028904.1 | MYB032; hypothetical protein; K09422 myb proto-oncogene protein, plant | | 0 | | 0.134 | | 1.126 | 0.386 | | | 5.228 | | 0.711 | | 1.393 |
| Pbr028978.1 | similar to MYB transcription factor MYB139; K09422 myb proto-oncogene protein, plant | | 0.741 | | 0.801 | | 4.928 | 0.964 | | | 1.238 | | 0.474 | | 1.14 |
| Pbr029248.1 | hypothetical protein LOC100262671; K09422 myb proto-oncogene protein, plant | | 5.066 | | 4.808 | | 1.69 | 6.074 | | | 5.916 | | 2.844 | | 3.672 |
| Pbr029249.1 | hypothetical protein LOC100262671; K09422 myb proto-oncogene protein, plant | | 0.247 | | 0.134 | | 0.563 | 0.096 | | | 3.715 | | 0.829 | | 0.76 |
| Pbr029686.2 | MADS1; MAD-box transcripion factor; K09264 MADS-box transcription factor, plant | | 4.818 | | 4.675 | | 16.895 | 8.292 | | | 13.071 | | 16.114 | | 13.549 |
| Pbr029876.1 | hypothetical protein LOC100244588; K09422 myb | | 0.247 | | 0.134 | | 0.141 | 0 | | | 3.577 | | 0.118 | | 1.013 |
| Pbr029990.1 | AP1; apetala1; K09264 MADS-box transcription factor, plant | | 80.184 | | 125.686 | | 193.73 | 79.446 | | | 70.582 | | 152.49 | | 182.218 |
| Pbr030542.1 | Ethylene-responsive transcription factor 1B, putative; K14516 ethylene-responsive transcription factor 1 | | 4.818 | | 2.271 | | 2.112 | 35.673 | | | 12.245 | | 2.844 | | 4.939 |
| Pbr030553.1 | hypothetical protein LOC100240829 | | 2.842 | | 3.072 | | 1.267 | 1.735 | | | 2.201 | | 1.422 | | 0.633 |
| Pbr030636.1 | hypothetical protein LOC100262843; K09287 RAV-like factor | | 0 | | 0 | | 0 | 0.193 | | | 2.201 | | 0 | | 0.253 |
| Pbr031306.1 | hypothetical protein LOC100241951; K09422 myb proto-oncogene protein, plant | | 0.741 | | 2.671 | | 0.563 | 0.771 | | | 4.953 | | 1.777 | | 0.633 |
| Pbr031456.1 | hypothetical protein LOC100267125; K09377 cysteine and glycine-rich protein | | 1.235 | | 0.134 | | 0.422 | 0.096 | | | 0 | | 0.237 | | 0.127 |
| Pbr031692.2 | hypothetical protein LOC100268119; K07466 replication factor A1 | | 18.162 | | 33.525 | | 16.332 | 19.283 | | | 18.437 | | 18.247 | | 15.069 |
| Pbr031830.1 | hypothetical protein LOC100246641; K09286 EREBP-like factor | | 4.942 | | 6.411 | | 11.827 | 7.713 | | | 7.017 | | 10.545 | | 12.03 |
| Pbr032023.1 | transcription factor, putative; K09422 myb proto-oncogene protein, plant | | 5.066 | | 12.689 | | 7.462 | 8.388 | | | 9.081 | | 5.806 | | 7.471 |
| Pbr032528.1 | hypothetical protein LOC100257755; K09422 myb proto-oncogene protein, plant | | 15.197 | | 22.172 | | 5.491 | 7.135 | | | 9.906 | | 9.834 | | 22.666 |
| Pbr032698.1 | WRKY transcription factor, putative; K13425 WRKY transcription factor 22 | | 36.324 | | 28.717 | | 6.476 | 10.895 | | | 15.96 | | 4.384 | | 7.471 |
| Pbr033396.1 | hypothetical protein LOC100248027; K09286 EREBP-like factor | | 559.185 | | 557.776 | | 815.185 | 423.453 | | | 708.16 | | 925.721 | | 993.653 |
| Pbr033430.1 | hypothetical protein LOC100248027; K09286 EREBP-like factor | | 502.105 | | 499.941 | | 726.909 | 373.51 | | | 632.074 | | 830.696 | | 887.665 |
| Pbr033541.1 | hypothetical protein LOC100256209; K09422 myb proto-oncogene protein, plant | | 27.552 | | 65.314 | | 10.559 | 19.186 | | | 60.263 | | 9.834 | | 12.536 |
| Pbr033560.1 | MYB167; hypothetical protein; K09422 myb proto-oncogene protein, plant | | 3.089 | | 0.134 | | 3.097 | 1.639 | | | 1.513 | | 2.607 | | 2.912 |
| Pbr033618.1 | MYB028; hypothetical protein; K09422 myb proto-oncogene protein, plant | | 5.436 | | 0 | | 0 | 1.735 | | | 0.275 | | 0 | | 0.127 |
| Pbr033937.1 | Protein AINTEGUMENTA, putative (EC:1.3.1.74); K09284 AP2-like factor, euAP2 lineage | | 8.896 | | 8.415 | | 6.195 | 6.845 | | | 16.098 | | 1.896 | | 3.039 |
| Pbr034455.1 | MYB184; hypothetical protein; K09422 myb proto-oncogene protein, plant | | 1.483 | | 0.134 | | 0.422 | 0.386 | | | 0.963 | | 0 | | 0 |
| Pbr035120.1 | transcription factor, putative; K13424 WRKY transcription factor 33 | | 2.1 | | 2.671 | | 2.253 | 1.446 | | | 1.651 | | 0.711 | | 2.026 |
| Pbr035294.1 | MADS9, PI; PISTILLATA-like MADS-box protein-like; K09264 MADS-box transcription factor, plant | | 3.706 | | 4.541 | | 0 | 1.157 | | | 1.101 | | 0.948 | | 1.646 |
| Pbr035515.1 | MYB052; hypothetical protein; K09422 myb proto-oncogene protein, plant | | 0.247 | | 1.469 | | 1.126 | 2.41 | | | 9.631 | | 3.555 | | 1.52 |
| Pbr035695.1 | AP2; transcription factor APETALA2; K09284 AP2-like factor, euAP2 lineage | | 7.166 | | 6.545 | | 4.928 | 5.592 | | | 3.852 | | 1.659 | | 2.406 |
| Pbr035774.1 | Ethylene-responsive transcription factor, putative; K09286 EREBP-like factor | | 4.695 | | 5.877 | | 2.393 | 3.857 | | | 3.44 | | 5.687 | | 13.929 |
| Pbr035788.1 | Ethylene-responsive transcription factor, putative; K09286 EREBP-like factor | | 5.066 | | 6.812 | | 2.112 | 4.242 | | | 3.99 | | 6.517 | | 15.829 |
| Pbr036590.1 | hypothetical protein LOC100243210; K09422 myb proto-oncogene protein, plant | | 0 | | 1.469 | | 17.177 | 11.57 | | | 4.265 | | 25.593 | | 14.436 |
| Pbr036591.1 | hypothetical protein LOC100243210; K09422 myb proto-oncogene protein, plant | | 0 | | 1.469 | | 17.177 | 11.57 | | | 4.265 | | 25.593 | | 14.436 |
| Pbr036605.1 | hypothetical protein; K14431 transcription factor TGA | | 0.124 | | 0 | | 1.69 | 0.675 | | | 0.275 | | 0.355 | | 0.886 |
| Pbr036688.1 | hypothetical protein LOC100251862; K13426 WRKY transcription factor 29 | | 38.177 | | 6.812 | | 0.422 | 12.437 | | | 6.467 | | 0.474 | | 1.52 |
| Pbr037679.1 | similar to MYC transcription factor; K13422 transcription factor MYC2 | | 167.904 | | 270.072 | | 49.277 | 108.756 | | | 161.527 | | 82.465 | | 72.178 |
| Pbr038408.1 | hypothetical protein LOC100261075; K07735 putative transcriptional regulator | | 4.942 | | 21.771 | | 10.137 | 27.864 | | | 6.879 | | 8.294 | | 6.965 |
| Pbr038434.1 | MYB083; hypothetical protein; K09422 myb proto-oncogene protein, plant | | 2.965 | | 7.48 | | 0.986 | 3.76 | | | 4.403 | | 2.962 | | 2.406 |
| Pbr038701.2 | r2r3-myb transcription factor | | 0.124 | | 0 | | 15.909 | 3.857 | | | 0.138 | | 14.929 | | 12.789 |
| Pbr038874.1 | hypothetical protein LOC100268025; K09422 myb proto-oncogene protein, plant | | 2.224 | | 2.137 | | 0 | 1.928 | | | 2.064 | | 0.592 | | 1.393 |
| Pbr038922.1 | hypothetical protein LOC100242243; K09422 myb proto-oncogene protein, plant | | 73.635 | | 34.594 | | 37.31 | 201.314 | | | 35.91 | | 2.725 | | 11.776 |
| Pbr038973.1 | hypothetical protein LOC100250334; K09250 cellular nucleic acid-binding protein | | 10.502 | | 17.631 | | 7.462 | 14.751 | | | 18.849 | | 12.796 | | 9.624 |
| Pbr039075.1 | hypothetical protein LOC100253313; K09422 myb proto-oncogene protein, plant | | 0.494 | | 3.206 | | 0.986 | 1.832 | | | 2.064 | | 0.711 | | 0.38 |
| Pbr039118.1 | hypothetical protein LOC100246639; | | 7.784 | | 8.949 | | 2.957 | 7.713 | | | 7.155 | | 1.422 | | 1.646 |
| Pbr039503.1 | hypothetical protein; K09264 MADS-box transcription factor, plant | | 41.389 | | 50.088 | | 18.162 | 25.839 | | | 37.561 | | 15.995 | | 22.033 |
| Pbr039833.1 | MYB025; hypothetical protein; K09422 myb proto-oncogene protein, plant | | 3.089 | | 3.473 | | 2.112 | 4.821 | | | 5.091 | | 1.896 | | 2.406 |
| Pbr039864.1 | MYB074; hypothetical protein; K09422 myb proto-oncogene protein, plant | | 3.954 | | 7.079 | | 10.559 | 7.231 | | | 7.017 | | 15.166 | | 15.575 |
| Pbr040390.1 | DNA binding protein, putative; K14432 ABA responsive element binding factor | | 19.521 | | 29.251 | | 31.397 | 11.184 | | | 26.967 | | 24.052 | | 19.501 |
| Pbr041094.1 | MYB028; hypothetical protein; K09422 myb proto-oncogene protein, plant | | 6.919 | | 0.401 | | 0.282 | 1.35 | | | 2.614 | | 0.355 | | 0.253 |
| Pbr041517.1 | hypothetical protein LOC100268119; K07466 replication factor A1 | | 3.089 | | 1.87 | | 2.534 | 1.253 | | | 1.238 | | 1.066 | | 1.013 |
| Pbr041525.1 | hypothetical protein LOC100244605; | | 1.235 | | 0.935 | | 0.141 | 0.675 | | | 2.752 | | 0.355 | | 0.38 |
| Pbr041889.1 | hypothetical protein LOC100259031; | | 0.124 | | 0.134 | | 13.094 | 4.049 | | | 0.826 | | 6.28 | | 8.104 |
| Pbr002230.1 | hypothetical protein LOC100251862; K13426 WRKY transcription factor 29 | | 36.818 | | 6.545 | | 0.422 | 11.955 | | | 6.329 | | 0.474 | | 1.52 |
|  |  | |  | |  | |  |  | | |  | |  | |  |
| Carbohydrate biosynthesis and metabolism | | | | | | | | | | | | | | | |
| Gene ID | Gene Description | TPM (transcripts per million clean tag) | | | | | | | | | | | | | |
|  |  | C1 | | C2 | | C3 | | | C4 | C5 | | C6 | | C7 | |
| Pbr000004.1 | Glycosyltransferase QUASIMODO1, putative; K13648 alpha-1,4-galacturonosyltransferase [EC:2.4.1.43] | 8.031 | | | 17.764 | 9.996 | | 7.81 | | 17.611 | | 11.967 | | 10.004 | |
| Pbr000007.1 | phosphofructokinase, putative (EC:2.7.1.11); K00850 6-phosphofructokinase [EC:2.7.1.11] | 30.393 | | | 19.768 | 63.92 | | 20.826 | | 1.513 | | 12.085 | | 34.57 | |
| Pbr000052.1 | L-galactose-1-phosphate phosphatase (EC:3.1.3.25); K10047 inositol-phosphate phosphatase / L-galactose 1-phosphate phosphatase [EC:3.1.3.25 3.1.3.-] | 40.648 | | | 71.458 | 47.728 | | 25.164 | | 39.35 | | 45.498 | | 51.411 | |
| Pbr000183.1 | pectinesterase family protein (EC:3.1.1.11); K01051 pectinesterase [EC:3.1.1.11] | 0.494 | | | 0.401 | 27.736 | | 2.025 | | 1.651 | | 8.886 | | 12.41 | |
| Pbr000988.3 | ascorbate peroxidase; K00434 L-ascorbate peroxidase [EC:1.11.1.11] | 541.517 | | | 834.125 | 652.711 | | 358.084 | | 687.384 | | 818.018 | | 766.861 | |
| Pbr001252.1 | hypothetical protein; K00001 alcohol dehydrogenase [EC:1.1.1.1] | 44.231 | | | 96.035 | 54.205 | | 15.619 | | 24.078 | | 65.167 | | 31.784 | |
| Pbr001360.1 | hypothetical protein LOC100242517; K00850 6-phosphofructokinase [EC:2.7.1.11] | 12.478 | | | 7.88 | 8.729 | | 14.269 | | 6.329 | | 4.028 | | 4.685 | |
| Pbr001447.1 | hypothetical protein LOC100261773; K00849 galactokinase [EC:2.7.1.6] | 3.459 | | | 4.007 | 6.336 | | 2.7 | | 5.503 | | 6.754 | | 4.052 | |
| Pbr001448.1 | hypothetical protein LOC100261773; K00849 galactokinase [EC:2.7.1.6] | 4.695 | | | 8.548 | 5.632 | | 3.471 | | 5.228 | | 7.109 | | 5.825 | |
| Pbr001483.1 | hypothetical protein; K11517 (S)-2-hydroxy-acid oxidase [EC:1.1.3.15] | 176.058 | | | 349.545 | 272.855 | | 166.026 | | 171.296 | | 221.092 | | 215.015 | |
| Pbr001560.1 | hydroxymethylglutaryl-CoA synthase, putative (EC:2.3.3.10); K01641 hydroxymethylglutaryl-CoA synthase [EC:2.3.3.10] | 151.719 | | | 130.361 | 40.407 | | 62.669 | | 175.148 | | 37.56 | | 56.983 | |
| Pbr001616.1 | hypothetical protein LOC100252799; K00695 sucrose synthase [EC:2.4.1.13] | 1.483 | | | 2.004 | 6.476 | | 8.292 | | 7.567 | | 2.725 | | 1.14 | |
| Pbr001726.1 | hypothetical protein LOC100245269; K06611 stachyose synthetase [EC:2.4.1.67] | 106.5 | | | 49.82 | 346.489 | | 40.301 | | 52.283 | | 128.437 | | 182.598 | |
| Pbr001745.1 | sucrose-phosphate synthase 1; K00696 sucrose-phosphate synthase [EC:2.4.1.14] | 1.73 | | | 4.274 | 6.476 | | 1.928 | | 6.742 | | 2.133 | | 1.773 | |
| Pbr001967.1 | phosphofructokinase, putative (EC:2.7.1.11); K00850 6-phosphofructokinase [EC:2.7.1.11] | 8.401 | | | 7.613 | 3.097 | | 10.22 | | 7.842 | | 3.673 | | 3.292 | |
| Pbr001995.1 | rbcL; ribulose-1,5-bisphosphate carboxylase/oxygenase large subunit (EC:4.1.1.39); K01601 ribulose-bisphosphate carboxylase large chain [EC:4.1.1.39] | 4.448 | | | 4.007 | 6.054 | | 27.575 | | 4.953 | | 46.091 | | 21.274 | |
| Pbr002010.1 | ATCBR; ATCBR (ARABIDOPSIS THALIANA NADH:CYTOCHROME B5 REDUCTASE 1); cytochrome-b5 reductase; K00326 cytochrome-b5 reductase [EC:1.6.2.2] | 73.759 | | | 139.31 | 162.192 | | 59.391 | | 113.784 | | 221.092 | | 139.798 | |
| Pbr002017.1 | UDP-glucuronate 5-epimerase, putative (EC:5.1.3.12); K08679 UDP-glucuronate 4-epimerase [EC:5.1.3.6] | 552.019 | | | 562.317 | 313.403 | | 155.324 | | 242.703 | | 333.416 | | 457.382 | |
| Pbr002191.1 | hypothetical protein; K01114 phospholipase C [EC:3.1.4.3] | 0.618 | | | 2.004 | 3.097 | | 1.446 | | 1.926 | | 2.37 | | 3.166 | |
| Pbr002423.1 | K12450 UDP-glucose 4,6-dehydratase [EC:4.2.1.76] | 18.285 | | | 36.731 | 25.202 | | 25.646 | | 49.944 | | 48.46 | | 32.797 | |
| Pbr002426.1 | Glycosyltransferase QUASIMODO1, putative; K13648 alpha-1,4-galacturonosyltransferase [EC:2.4.1.43] | 5.56 | | | 5.743 | 1.549 | | 3.76 | | 6.879 | | 2.133 | | 1.393 | |
| Pbr002553.1 | malate dehydrogenase, putative (EC:1.1.1.37); K00025 malate dehydrogenase [EC:1.1.1.37] | 1.73 | | | 1.069 | 3.097 | | 4.531 | | 3.44 | | 1.659 | | 0.633 | |
| Pbr002557.1 | hypothetical protein LOC100262240; K00135 succinate-semialdehyde dehydrogenase (NADP+) [EC:1.2.1.16] | 5.436 | | | 13.49 | 7.321 | | 10.22 | | 15.685 | | 9.005 | | 11.27 | |
| Pbr002689.1 | hypothetical protein LOC100253745; K12450 UDP-glucose 4,6-dehydratase [EC:4.2.1.76] | 0.371 | | | 2.938 | 0 | | 0.675 | | 1.238 | | 0.711 | | 0.127 | |
| Pbr002800.2 | malic enzyme, putative (EC:1.1.1.40); K00029 malate dehydrogenase (oxaloacetate-decarboxylating)(NADP+) [EC:1.1.1.40] | 151.719 | | | 221.187 | 125.727 | | 148.382 | | 218.35 | | 112.916 | | 109.28 | |
| Pbr002935.1 | hypothetical protein LOC100248541; K07407 alpha-galactosidase [EC:3.2.1.22] | 126.885 | | | 110.059 | 177.116 | | 207.195 | | 48.568 | | 85.072 | | 80.409 | |
| Pbr002973.1 | hypothetical protein LOC100248541; K07407 alpha-galactosidase [EC:3.2.1.22] | 134.422 | | | 120.21 | 188.943 | | 221.561 | | 52.971 | | 90.404 | | 86.487 | |
| Pbr003011.1 | alpha-amylase, putative (EC:3.2.1.60); K01176 alpha-amylase [EC:3.2.1.1] | 293.801 | | | 76.667 | 33.086 | | 255.113 | | 32.058 | | 24.882 | | 25.959 | |
| Pbr003019.1 | alpha-amylase, putative (EC:3.2.1.60); K01176 alpha-amylase [EC:3.2.1.1] | 293.801 | | | 76.667 | 33.086 | | 255.113 | | 32.058 | | 24.882 | | 25.959 | |
| Pbr003394.1 | PtrSuSY2; sucrose synthase (EC:2.4.1.13); K00695 sucrose synthase [EC:2.4.1.13] | 6.425 | | | 8.548 | 64.201 | | 21.308 | | 15.685 | | 122.513 | | 54.197 | |
| Pbr003395.1 | PtrSuSY2; sucrose synthase (EC:2.4.1.13); K00695 sucrose synthase [EC:2.4.1.13] | 6.795 | | | 11.22 | 52.938 | | 17.258 | | 18.987 | | 95.736 | | 46.093 | |
| Pbr003449.1 | hypothetical protein LOC100246616; K00382 dihydrolipoamide dehydrogenase [EC:1.8.1.4] | 24.092 | | | 34.594 | 15.206 | | 19.958 | | 48.293 | | 29.147 | | 19.121 | |
| Pbr003592.1 | glucose-1-phosphate adenylyltransferase, putative (EC:2.7.7.27); K00975 glucose-1-phosphate adenylyltransferase [EC:2.7.7.27] | 6.301 | | | 19.768 | 5.632 | | 10.124 | | 13.759 | | 5.806 | | 7.091 | |
| Pbr004366.1 | dtdp-glucose 4-6-dehydratase, putative (EC:5.1.3.18); K10046 GDP-D-mannose 3', 5'-epimerase [EC:5.1.3.18 5.1.3.-] | 187.919 | | | 392.153 | 140.651 | | 138.162 | | 280.402 | | 267.42 | | 270.858 | |
| Pbr004627.1 | hypothetical protein LOC100245165; K01176 alpha-amylase | 43.366 | | | 11.887 | 6.336 | | 41.073 | | 5.779 | | 1.422 | | 2.153 | |
| Pbr004628.1 | hypothetical protein LOC100245165; K01176 alpha-amylase [EC:3.2.1.1] | 45.096 | | | 20.569 | 16.332 | | 34.998 | | 18.299 | | 6.754 | | 11.017 | |
| Pbr004785.2 | hypothetical protein LOC100262362; K12449 UDP-apiose/xylose synthase | 199.532 | | | 234.677 | 115.731 | | 208.255 | | 303.379 | | 229.149 | | 126.882 | |
| Pbr004998.1 | hypothetical protein LOC100240836; K12373 hexosaminidase [EC:3.2.1.52] | 16.185 | | | 14.559 | 8.166 | | 10.606 | | 11.97 | | 10.071 | | 5.952 | |
| Pbr005161.1 | pyrophosphate-dependent phosphofructokinase (EC:2.7.1.90); K00895 pyrophosphate--fructose-6-phosphate 1-phosphotransferase [EC:2.7.1.90] | 14.332 | | | 22.706 | 17.317 | | 14.173 | | 19.262 | | 9.479 | | 9.624 | |
| Pbr005280.1 | Pectate lyase precursor, putative (EC:4.2.2.2); K01728 pectate lyase [EC:4.2.2.2] | 5.807 | | | 0.935 | 1.549 | | 17.547 | | 9.493 | | 1.54 | | 0.633 | |
| Pbr005388.1 | trehalose-6-phosphate synthase, putative (EC:2.4.1.15 3.1.3.12); K00697 alpha,alpha-trehalose-phosphate synthase (UDP-forming) [EC:2.4.1.15]; K01087 trehalose-phosphatase [EC:3.1.3.12] | 45.59 | | | 30.587 | 15.769 | | 55.246 | | 28.343 | | 18.721 | | 19.627 | |
| Pbr005501.1 | hypothetical protein LOC100245998; K01623 fructose-bisphosphate aldolase, class I [EC:4.1.2.13] | 27.552 | | | 77.202 | 118.688 | | 18.704 | | 14.722 | | 154.741 | | 68.886 | |
| Pbr005502.1 | fructose-bisphosphate aldolase, putative (EC:4.1.2.13); K01623 fructose-bisphosphate aldolase, class I [EC:4.1.2.13] | 429.211 | | | 611.737 | 543.034 | | 321.832 | | 442.067 | | 734.605 | | 646.058 | |
| Pbr005571.1 | similar to Citrate synthase, glyoxysomal; K01647 citrate synthase [EC:2.3.3.1] | 178.158 | | | 106.453 | 94.331 | | 156.192 | | 51.045 | | 57.11 | | 92.439 | |
| Pbr005714.1 | phosphoenolpyruvate carboxykinase (ATP) (EC:4.1.1.49); K01610 phosphoenolpyruvate carboxykinase (ATP) [EC:4.1.1.49] | 7.537 | | | 17.631 | 9.855 | | 6.171 | | 7.842 | | 11.019 | | 11.017 | |
| Pbr005718.1 | Beta-fructofuranosidase, insoluble isoenzyme 3 precursor, putative (EC:3.2.1.26); K01193 beta-fructofuranosidase | 10.131 | | | 3.473 | 0.704 | | 0.386 | | 0.688 | | 0 | | 0.886 | |
| Pbr005730.1 | hypothetical protein; K14190 GDP-L-galactose phosphorylase [EC:2.7.7.69] | 87.967 | | | 110.059 | 84.616 | | 91.979 | | 81.176 | | 50.948 | | 53.564 | |
| Pbr005767.1 | alcohol dehydrogenase, putative (EC:1.1.1.284); K00121 S-(hydroxymethyl)glutathione dehydrogenase / alcohol dehydrogenase [EC:1.1.1.284 1.1.1.1] | 14.208 | | | 35.262 | 14.642 | | 15.137 | | 21.601 | | 20.261 | | 13.423 | |
| Pbr005841.1 | hypothetical protein LOC100255753; K00844 hexokinase | 6.548 | | | 15.227 | 11.123 | | 11.57 | | 15.822 | | 18.128 | | 15.069 | |
| Pbr006178.1 | similar to glucose-6-phosphate isomerase; K01810 glucose-6-phosphate isomerase [EC:5.3.1.9] | 63.999 | | | 120.611 | 101.089 | | 75.3 | | 62.464 | | 127.253 | | 78.51 | |
| Pbr006369.1 | hypothetical protein; K01193 beta-fructofuranosidase [EC:3.2.1.26] | 9.513 | | | 4.408 | 8.729 | | 32.974 | | 19.125 | | 3.081 | | 9.75 | |
| Pbr006403.1 | hypothetical protein LOC100242366; K13648 alpha-1,4-galacturonosyltransferase [EC:2.4.1.43] | 17.915 | | | 9.483 | 6.336 | | 7.81 | | 9.218 | | 2.844 | | 2.279 | |
| Pbr006420.1 | Biotin carboxyl carrier protein subunit of of Het-ACCase (BCCP2) (EC:6.4.1.2); K02160 acetyl-CoA carboxylase biotin carboxyl carrier protein | 7.289 | | | 12.288 | 4.224 | | 10.895 | | 18.437 | | 8.886 | | 6.205 | |
| Pbr006528.1 | hypothetical protein LOC100245165; K01176 alpha-amylase [EC:3.2.1.1] | 143.688 | | | 47.016 | 18.585 | | 173.835 | | 26.829 | | 3.91 | | 7.091 | |
| Pbr006591.1 | rbcL; ribulose-1,5-bisphosphate carboxylase/oxygenase large subunit (EC:4.1.1.39); K01601 ribulose-bisphosphate carboxylase large chain [EC:4.1.1.39] | 4.448 | | | 4.007 | 6.054 | | 27.575 | | 4.953 | | 46.091 | | 21.274 | |
| Pbr006623.4 | hypothetical protein; K01580 glutamate decarboxylase [EC:4.1.1.15] | 186.313 | | | 295.45 | 233.011 | | 261.476 | | 427.07 | | 268.131 | | 193.235 | |
| Pbr007072.2 | pyruvate kinase, putative (EC:2.7.1.40); K00873 pyruvate kinase [EC:2.7.1.40] | 1.235 | | | 1.069 | 0.282 | | 0.386 | | 0.55 | | 0 | | 0.127 | |
| Pbr007150.1 | enolase, putative (EC:4.2.1.11); K01689 enolase [EC:4.2.1.11] | 15.444 | | | 22.439 | 7.744 | | 14.366 | | 30.269 | | 16.232 | | 16.208 | |
| Pbr007177.1 | hypothetical protein LOC100262181; K13648 alpha-1,4-galacturonosyltransferase [EC:2.4.1.43] | 9.019 | | | 4.408 | 3.661 | | 3.182 | | 5.779 | | 1.303 | | 2.786 | |
| Pbr007232.1 | hypothetical protein; K00012 UDPglucose 6-dehydrogenase [EC:1.1.1.22] | 22.98 | | | 7.613 | 9.433 | | 16.005 | | 26.142 | | 30.451 | | 11.397 | |
| Pbr007298.1 | malate dehydrogenase (EC:1.1.1.37); K00026 malate dehydrogenase [EC:1.1.1.37] | 69.929 | | | 35.662 | 22.104 | | 42.422 | | 57.786 | | 25.356 | | 26.972 | |
| Pbr007409.1 | Polygalacturonase precursor, putative (EC:3.2.1.67); K01184 polygalacturonase [EC:3.2.1.15] | 63.875 | | | 2.004 | 0.141 | | 93.329 | | 4.265 | | 0.118 | | 0.127 | |
| Pbr007632.1 | Beta-fructofuranosidase, insoluble isoenzyme 3 precursor, putative (EC:3.2.1.26); K01193 beta-fructofuranosidase [EC:3.2.1.26] | 10.255 | | | 3.74 | 0.704 | | 0.386 | | 0.688 | | 0 | | 0.76 | |
| Pbr007764.1 | hypothetical protein; K01176 alpha-amylase [EC:3.2.1.1] | 1.359 | | | 0.801 | 0.141 | | 1.253 | | 0.138 | | 0 | | 0.253 | |
| Pbr007834.1 | isocitrate dehydrogenase (NAD+) (EC:1.1.1.41) | 2.471 | | | 1.736 | 1.971 | | 2.314 | | 3.715 | | 1.896 | | 1.266 | |
| Pbr007869.2 | phosphoglycerate/bisphosphoglycerate mutase family protein; K01834 phosphoglycerate mutase [EC:5.4.2.1] | 11.49 | | | 15.227 | 10.559 | | 11.763 | | 18.299 | | 8.294 | | 10.89 | |
| Pbr007870.2 | phosphoglycerate/bisphosphoglycerate mutase family protein; K01834 phosphoglycerate mutase [EC:5.4.2.1] | 7.413 | | | 5.209 | 2.957 | | 3.471 | | 9.081 | | 3.673 | | 2.659 | |
| Pbr008035.1 | sucrose phosphate syntase, putative (EC:2.4.1.14); K00696 sucrose-phosphate synthase [EC:2.4.1.14] | 2.842 | | | 7.88 | 10.7 | | 4.435 | | 11.97 | | 3.555 | | 3.546 | |
| Pbr008172.1 | hypothetical protein LOC100250604; K00889 1-phosphatidylinositol-4-phosphate 5-kinase [EC:2.7.1.68] | 17.915 | | | 21.371 | 11.686 | | 11.088 | | 20.225 | | 9.597 | | 5.825 | |
| Pbr008291.1 | hypothetical protein; K00434 L-ascorbate peroxidase [EC:1.11.1.11] | 152.213 | | | 148.526 | 223.296 | | 31.624 | | 19.262 | | 111.257 | | 122.576 | |
| Pbr008441.1 | alpha-galactosidase (EC:2.4.1.67); K07407 alpha-galactosidase [EC:3.2.1.22] | 19.891 | | | 39.135 | 19.007 | | 25.164 | | 57.099 | | 22.986 | | 16.082 | |
| Pbr008457.1 | hypothetical protein LOC100266759; K00695 sucrose synthase [EC:2.4.1.13] | 1.73 | | | 1.736 | 2.112 | | 1.735 | | 3.027 | | 0.711 | | 1.013 | |
| Pbr008464.1 | hypothetical protein LOC100266759; K00695 sucrose synthase [EC:2.4.1.13] | 1.606 | | | 1.603 | 1.971 | | 1.735 | | 3.027 | | 0.711 | | 1.013 | |
| Pbr008471.1 | utp-glucose-1-phosphate uridylyltransferase, putative (EC:2.7.7.9); K00963 UTP--glucose-1-phosphate uridylyltransferase [EC:2.7.7.9] | 161.726 | | | 204.625 | 292.566 | | 143.176 | | 155.473 | | 375.952 | | 267.819 | |
| Pbr008479.1 | hypothetical protein LOC100266215; K01580 glutamate decarboxylase [EC:4.1.1.15] | 139.611 | | | 188.062 | 79.266 | | 117.337 | | 183.678 | | 109.717 | | 108.141 | |
| Pbr008480.1 | hypothetical protein; K01580 glutamate decarboxylase [EC:4.1.1.15] | 146.777 | | | 192.203 | 84.334 | | 121 | | 185.605 | | 118.485 | | 117.258 | |
| Pbr008481.1 | hypothetical protein; K01580 glutamate decarboxylase [EC:4.1.1.15] | 73.635 | | | 113.532 | 32.101 | | 52.257 | | 104.704 | | 50.474 | | 56.729 | |
| Pbr008522.1 | hypothetical protein LOC100250604; K00889 1-phosphatidylinositol-4-phosphate 5-kinase [EC:2.7.1.68] | 10.131 | | | 9.35 | 4.646 | | 9.256 | | 13.208 | | 3.436 | | 3.292 | |
| Pbr008761.1 | phosphofructokinase, putative (EC:2.7.1.11); K00850 6-phosphofructokinase [EC:2.7.1.11] | 46.207 | | | 74.53 | 26.047 | | 20.633 | | 42.377 | | 54.621 | | 31.91 | |
| Pbr009063.1 | Glycosyltransferase QUASIMODO1, putative; K13648 alpha-1,4-galacturonosyltransferase [EC:2.4.1.43] | 17.05 | | | 6.144 | 7.884 | | 5.399 | | 3.44 | | 2.844 | | 4.052 | |
| Pbr009268.1 | hypothetical protein LOC100267958; K12446 L-arabinokinase | 39.289 | | | 35.662 | 50.404 | | 58.62 | | 15.822 | | 16.943 | | 16.715 | |
| Pbr009290.1 | hypothetical protein LOC100253745; K12450 UDP-glucose 4,6-dehydratase [EC:4.2.1.76] | 0.371 | | | 2.938 | 0 | | 0.675 | | 1.238 | | 0.711 | | 0.127 | |
| Pbr009527.1 | phosphoinositide phospholipase C (EC:3.1.4.11); K05857 phospholipase C, delta [EC:3.1.4.11] | 10.625 | | | 10.418 | 4.083 | | 12.534 | | 9.769 | | 3.792 | | 6.711 | |
| Pbr009528.1 | 1-phosphatidylinositol-4,5-bisphosphate phosphodiesterase, | 20.262 | | | 43.81 | 85.742 | | 38.277 | | 40.726 | | 89.93 | | 77.37 | |
| Pbr009578.1 | hypothetical protein; K00696 sucrose-phosphate synthase [EC:2.4.1.14] | 20.015 | | | 20.836 | 19.57 | | 16.39 | | 11.145 | | 6.161 | | 6.838 | |
| Pbr009623.1 | rbcL; ribulose-1,5-bisphosphate carboxylase/oxygenase large subunit (EC:4.1.1.39); K01601 ribulose-bisphosphate carboxylase large chain [EC:4.1.1.39] | 4.448 | | | 4.007 | 5.772 | | 27.189 | | 4.953 | | 45.143 | | 20.767 | |
| Pbr009764.1 | hypothetical protein; K01183 chitinase [EC:3.2.1.14] | 131.457 | | | 49.687 | 13.798 | | 74.432 | | 136.624 | | 4.739 | | 15.322 | |
| Pbr009765.1 | hypothetical protein; K01183 chitinase [EC:3.2.1.14] | 129.603 | | | 47.416 | 13.094 | | 72.793 | | 133.872 | | 4.384 | | 15.195 | |
| Pbr009785.1 | hypothetical protein; K01183 chitinase [EC:3.2.1.14] | 64.74 | | | 25.511 | 5.913 | | 34.227 | | 69.894 | | 2.607 | | 8.484 | |
| Pbr009786.1 | hypothetical protein; K01183 chitinase [EC:3.2.1.14] | 131.457 | | | 49.687 | 13.798 | | 74.432 | | 136.624 | | 4.739 | | 15.322 | |
| Pbr010008.1 | rbcL; ribulose-1,5-bisphosphate carboxylase/oxygenase large subunit (EC:4.1.1.39); K01601 ribulose-bisphosphate carboxylase large chain [EC:4.1.1.39] | 4.448 | | | 4.007 | 6.054 | | 27.575 | | 4.953 | | 46.091 | | 21.274 | |
| Pbr010442.1 | Pectinesterase-2 precursor, putative (EC:3.1.1.11); K01051 pectinesterase [EC:3.1.1.11] | 7.166 | | | 14.826 | 5.35 | | 2.025 | | 6.742 | | 8.057 | | 8.611 | |
| Pbr010451.1 | Pectinesterase-2 precursor, putative (EC:3.1.1.11); K01051 pectinesterase [EC:3.1.1.11] | 7.166 | | | 14.826 | 5.35 | | 2.025 | | 6.742 | | 8.057 | | 8.611 | |
| Pbr010484.1 | rbcL; ribulose-1,5-bisphosphate carboxylase/oxygenase large subunit (EC:4.1.1.39); K01601 ribulose-bisphosphate carboxylase large chain [EC:4.1.1.39] | 4.448 | | | 4.007 | 6.054 | | 27.575 | | 4.953 | | 46.091 | | 21.274 | |
| Pbr010532.1 | fructose-bisphosphatase (EC:3.1.3.11); K03841 fructose-1,6-bisphosphatase I [EC:3.1.3.11] | 33.482 | | | 58.502 | 57.865 | | 35.866 | | 23.665 | | 39.455 | | 39.888 | |
| Pbr010726.1 | hypothetical protein; K01728 pectate lyase [EC:4.2.2.2] | 15.691 | | | 40.204 | 18.021 | | 27.092 | | 65.079 | | 20.142 | | 17.095 | |
| Pbr010727.1 | hypothetical protein LOC100246124; K01728 pectate lyase [EC:4.2.2.2] | 10.749 | | | 25.645 | 8.025 | | 15.234 | | 34.809 | | 13.626 | | 8.484 | |
| Pbr010732.1 | hypothetical protein LOC100243846 | 22.239 | | | 27.915 | 20.133 | | 34.516 | | 47.467 | | 20.616 | | 24.566 | |
| Pbr000004.1 | Glycosyltransferase QUASIMODO1, | 8.031 | | | 17.764 | 9.996 | | 7.81 | | 17.611 | | 11.967 | | 10.004 | |
| Pbr000007.1 | phosphofructokinase, putative (EC:2.7.1.11); K00850 6-phosphofructokinase [EC:2.7.1.11] | 30.393 | | | 19.768 | 63.92 | | 20.826 | | 1.513 | | 12.085 | | 34.57 | |
| Pbr000052.1 | L-galactose-1-phosphate phosphatase (EC:3.1.3.25); K10047 inositol-phosphate phosphatase / L-galactose 1-phosphate phosphatase [EC:3.1.3.25 3.1.3.-] | 40.648 | | | 71.458 | 47.728 | | 25.164 | | 39.35 | | 45.498 | | 51.411 | |
| Pbr000183.1 | pectinesterase family protein (EC:3.1.1.11); K01051 pectinesterase [EC:3.1.1.11] | 0.494 | | | 0.401 | 27.736 | | 2.025 | | 1.651 | | 8.886 | | 12.41 | |
| Pbr000988.3 | ascorbate peroxidase; K00434 L-ascorbate peroxidase [EC:1.11.1.11] | 541.517 | | | 834.125 | 652.711 | | 358.084 | | 687.384 | | 818.018 | | 766.861 | |
| Pbr001252.1 | hypothetical protein; K00001 alcohol dehydrogenase [EC:1.1.1.1] | 44.231 | | | 96.035 | 54.205 | | 15.619 | | 24.078 | | 65.167 | | 31.784 | |
| Pbr001360.1 | hypothetical protein LOC100242517; K00850 6-phosphofructokinase [EC:2.7.1.11] | 12.478 | | | 7.88 | 8.729 | | 14.269 | | 6.329 | | 4.028 | | 4.685 | |
| Pbr001447.1 | hypothetical protein LOC100261773; K00849 galactokinase [EC:2.7.1.6] | 3.459 | | | 4.007 | 6.336 | | 2.7 | | 5.503 | | 6.754 | | 4.052 | |
| Pbr001448.1 | hypothetical protein LOC100261773; K00849 galactokinase [EC:2.7.1.6] | 4.695 | | | 8.548 | 5.632 | | 3.471 | | 5.228 | | 7.109 | | 5.825 | |
| Pbr001483.1 | hypothetical protein; K11517 (S)-2-hydroxy-acid oxidase [EC:1.1.3.15] | 176.058 | | | 349.545 | 272.855 | | 166.026 | | 171.296 | | 221.092 | | 215.015 | |
| Pbr001560.1 | hydroxymethylglutaryl-CoA synthase, putative (EC:2.3.3.10); K01641 hydroxymethylglutaryl-CoA synthase [EC:2.3.3.10] | 151.719 | | | 130.361 | 40.407 | | 62.669 | | 175.148 | | 37.56 | | 56.983 | |
| Pbr001616.1 | hypothetical protein LOC100252799; K00695 sucrose synthase [EC:2.4.1.13] | 1.483 | | | 2.004 | 6.476 | | 8.292 | | 7.567 | | 2.725 | | 1.14 | |
| Pbr001726.1 | hypothetical protein LOC100245269 | 106.5 | | | 49.82 | 346.489 | | 40.301 | | 52.283 | | 128.437 | | 182.598 | |
| Pbr001745.1 | sucrose-phosphate synthase 1; K00696 sucrose-phosphate synthase [EC:2.4.1.14] | 1.73 | | | 4.274 | 6.476 | | 1.928 | | 6.742 | | 2.133 | | 1.773 | |
| Pbr001967.1 | phosphofructokinase, putative (EC:2.7.1.11); K00850 6-phosphofructokinase [EC:2.7.1.11] | 8.401 | | | 7.613 | 3.097 | | 10.22 | | 7.842 | | 3.673 | | 3.292 | |
| Pbr001995.1 | rbcL; ribulose-1,5-bisphosphate carboxylase/oxygenase large subunit (EC:4.1.1.39); K01601 ribulose-bisphosphate carboxylase large chain [EC:4.1.1.39] | 4.448 | | | 4.007 | 6.054 | | 27.575 | | 4.953 | | 46.091 | | 21.274 | |
| Pbr002010.1 | ATCBR; ATCBR (ARABIDOPSIS THALIANA NADH | 73.759 | | | 139.31 | 162.192 | | 59.391 | | 113.784 | | 221.092 | | 139.798 | |
| Pbr002017.1 | UDP-glucuronate 5-epimerase, putative (EC:5.1.3.12); K08679 UDP-glucuronate 4-epimerase [EC:5.1.3.6] | 552.019 | | | 562.317 | 313.403 | | 155.324 | | 242.703 | | 333.416 | | 457.382 | |
| Pbr002191.1 | hypothetical protein; K01114 phospholipase C [EC:3.1.4.3] | 0.618 | | | 2.004 | 3.097 | | 1.446 | | 1.926 | | 2.37 | | 3.166 | |
| Pbr002423.1 | hypothetical protein LOC100260586; K12450 UDP-glucose 4,6-dehydratase [EC:4.2.1.76] | 18.285 | | | 36.731 | 25.202 | | 25.646 | | 49.944 | | 48.46 | | 32.797 | |
| Pbr002426.1 | Glycosyltransferase QUASIMODO1, putative; K13648 alpha-1,4-galacturonosyltransferase [EC:2.4.1.43] | 5.56 | | | 5.743 | 1.549 | | 3.76 | | 6.879 | | 2.133 | | 1.393 | |
| Pbr002553.1 | malate dehydrogenase, putative (EC:1.1.1.37); K00025 malate dehydrogenase [EC:1.1.1.37] | 1.73 | | | 1.069 | 3.097 | | 4.531 | | 3.44 | | 1.659 | | 0.633 | |
| Pbr002557.1 | hypothetical protein LOC100262240; K00135 succinate-semialdehyde dehydrogenase (NADP+) [EC:1.2.1.16] | 5.436 | | | 13.49 | 7.321 | | 10.22 | | 15.685 | | 9.005 | | 11.27 | |
| Pbr002689.1 | hypothetical protein LOC100253745; K12450 UDP-glucose 4,6-dehydratase [EC:4.2.1.76] | 0.371 | | | 2.938 | 0 | | 0.675 | | 1.238 | | 0.711 | | 0.127 | |
| Pbr002800.2 | malic enzyme, putative (EC:1.1.1.40); K00029 malate dehydrogenase (oxaloacetate-decarboxylating) | 151.719 | | | 221.187 | 125.727 | | 148.382 | | 218.35 | | 112.916 | | 109.28 | |
| Pbr002935.1 | hypothetical protein LOC100248541; K07407 alpha-galactosidase [EC:3.2.1.22] | 126.885 | | | 110.059 | 177.116 | | 207.195 | | 48.568 | | 85.072 | | 80.409 | |
| Pbr002973.1 | hypothetical protein LOC100248541; K07407 alpha-galactosidase [EC:3.2.1.22] | 134.422 | | | 120.21 | 188.943 | | 221.561 | | 52.971 | | 90.404 | | 86.487 | |
| Pbr003011.1 | alpha-amylase, putative (EC:3.2.1.60); K01176 alpha-amylase [EC:3.2.1.1] | 293.801 | | | 76.667 | 33.086 | | 255.113 | | 32.058 | | 24.882 | | 25.959 | |
| Pbr003019.1 | alpha-amylase, putative (EC:3.2.1.60); K01176 alpha-amylase [EC:3.2.1.1] | 293.801 | | | 76.667 | 33.086 | | 255.113 | | 32.058 | | 24.882 | | 25.959 | |
| Pbr003394.1 | PtrSuSY2; sucrose synthase (EC:2.4.1.13); K00695 sucrose synthase [EC:2.4.1.13] | 6.425 | | | 8.548 | 64.201 | | 21.308 | | 15.685 | | 122.513 | | 54.197 | |
| Pbr003395.1 | PtrSuSY2; sucrose synthase (EC:2.4.1.13); K00695 sucrose synthase [EC:2.4.1.13] | 6.795 | | | 11.22 | 52.938 | | 17.258 | | 18.987 | | 95.736 | | 46.093 | |
| Pbr003449.1 | hypothetical protein LOC100246616; K00382 dihydrolipoamide dehydrogenase [EC:1.8.1.4] | 24.092 | | | 34.594 | 15.206 | | 19.958 | | 48.293 | | 29.147 | | 19.121 | |
| Pbr003592.1 | glucose-1-phosphate adenylyltransferase, putative (EC:2.7.7.27); K00975 glucose-1-phosphate | 6.301 | | | 19.768 | 5.632 | | 10.124 | | 13.759 | | 5.806 | | 7.091 | |
| Pbr004366.1 | dtdp-glucose 4-6-dehydratase, putative (EC:5.1.3.18); K10046 GDP-D-mannose 3', 5'-epimerase [EC:5.1.3.18 5.1.3.-] | 187.919 | | | 392.153 | 140.651 | | 138.162 | | 280.402 | | 267.42 | | 270.858 | |
| Pbr004627.1 | hypothetical protein LOC100245165; K01176 alpha-amylase [EC:3.2.1.1] | 43.366 | | | 11.887 | 6.336 | | 41.073 | | 5.779 | | 1.422 | | 2.153 | |
| Pbr004628.1 | hypothetical protein LOC100245165; K01176 alpha-amylase [EC:3.2.1.1] | 45.096 | | | 20.569 | 16.332 | | 34.998 | | 18.299 | | 6.754 | | 11.017 | |
| Pbr004785.2 | hypothetical protein LOC100262362; K12449 UDP-apiose/xylose synthase | 199.532 | | | 234.677 | 115.731 | | 208.255 | | 303.379 | | 229.149 | | 126.882 | |
| Pbr004998.1 | hypothetical protein LOC100240836; K12373 hexosaminidase | 16.185 | | | 14.559 | 8.166 | | 10.606 | | 11.97 | | 10.071 | | 5.952 | |
| Pbr005161.1 | pyrophosphate-dependent phosphofructokinase (EC:2.7.1.90); K00895 pyrophosphate--fructose-6-phosphate 1-phosphotransferase [EC:2.7.1.90] | 14.332 | | | 22.706 | 17.317 | | 14.173 | | 19.262 | | 9.479 | | 9.624 | |
| Pbr005280.1 | Pectate lyase precursor, putative (EC:4.2.2.2); K01728 pectate lyase [EC:4.2.2.2] | 5.807 | | | 0.935 | 1.549 | | 17.547 | | 9.493 | | 1.54 | | 0.633 | |
| Pbr005388.1 | trehalose-6-phosphate synthase, putative (EC:2.4.1.15 3.1.3.12); K00697 alpha,alpha-trehalose-phosphate synthase (UDP-forming) [EC:2.4.1.15]; K01087 trehalose-phosphatase [EC:3.1.3.12] | 45.59 | | | 30.587 | 15.769 | | 55.246 | | 28.343 | | 18.721 | | 19.627 | |
| Pbr005501.1 | hypothetical protein LOC100245998; K01623 fructose-bisphosphate aldolase, class I [EC:4.1.2.13] | 27.552 | | | 77.202 | 118.688 | | 18.704 | | 14.722 | | 154.741 | | 68.886 | |
| Pbr005502.1 | fructose-bisphosphate aldolase, putative (EC:4.1.2.13); K01623 fructose-bisphosphate aldolase, class I [EC:4.1.2.13] | 429.211 | | | 611.737 | 543.034 | | 321.832 | | 442.067 | | 734.605 | | 646.058 | |
| Pbr005571.1 | similar to Citrate synthase, glyoxysomal; K01647 citrate synthase [EC:2.3.3.1] | 178.158 | | | 106.453 | 94.331 | | 156.192 | | 51.045 | | 57.11 | | 92.439 | |
| Pbr005714.1 | phosphoenolpyruvate carboxykinase (ATP) (EC:4.1.1.49); K01610 phosphoenolpyruvate carboxykinase (ATP) [EC:4.1.1.49] | 7.537 | | | 17.631 | 9.855 | | 6.171 | | 7.842 | | 11.019 | | 11.017 | |
| Pbr005718.1 | Beta-fructofuranosidase, insoluble isoenzyme 3 precursor, putative (EC:3.2.1.26); K01193 beta-fructofuranosidase [EC:3.2.1.26] | 10.131 | | | 3.473 | 0.704 | | 0.386 | | 0.688 | | 0 | | 0.886 | |
| Pbr005730.1 | hypothetical protein; K14190 GDP-L-galactose phosphorylase [EC:2.7.7.69] | 87.967 | | | 110.059 | 84.616 | | 91.979 | | 81.176 | | 50.948 | | 53.564 | |
| Pbr005767.1 | alcohol dehydrogenase, putative (EC:1.1.1.284); K00121 S-(hydroxymethyl)glutathione dehydrogenase / alcohol dehydrogenase [EC:1.1.1.284 1.1.1.1] | 14.208 | | | 35.262 | 14.642 | | 15.137 | | 21.601 | | 20.261 | | 13.423 | |
| Pbr005841.1 | hypothetical protein LOC100255753; K00844 hexokinase [EC:2.7.1.1] | 6.548 | | | 15.227 | 11.123 | | 11.57 | | 15.822 | | 18.128 | | 15.069 | |
| Pbr006178.1 | similar to glucose-6-phosphate isomerase; K01810 glucose-6-phosphate isomerase [EC:5.3.1.9] | 63.999 | | | 120.611 | 101.089 | | 75.3 | | 62.464 | | 127.253 | | 78.51 | |
| Pbr006369.1 | hypothetical protein; K01193 beta-fructofuranosidase [EC:3.2.1.26] | 9.513 | | | 4.408 | 8.729 | | 32.974 | | 19.125 | | 3.081 | | 9.75 | |
| Pbr006403.1 | hypothetical protein LOC100242366; K13648 alpha-1,4-galacturonosyltransferase [EC:2.4.1.43] | 17.915 | | | 9.483 | 6.336 | | 7.81 | | 9.218 | | 2.844 | | 2.279 | |
| Pbr006420.1 | Biotin carboxyl carrier protein subunit of of Het-ACCase (BCCP2) (EC:6.4.1.2); K02160 acetyl-CoA carboxylase biotin carboxyl carrier protein | 7.289 | | | 12.288 | 4.224 | | 10.895 | | 18.437 | | 8.886 | | 6.205 | |
| Pbr006528.1 | hypothetical protein LOC100245165; K01176 alpha-amylase [EC:3.2.1.1] | 143.688 | | | 47.016 | 18.585 | | 173.835 | | 26.829 | | 3.91 | | 7.091 | |
| Pbr006591.1 | rbcL; ribulose-1,5-bisphosphate carboxylase/oxygenase large subunit (EC:4.1.1.39); K01601 ribulose-bisphosphate carboxylase large chain [EC:4.1.1.39] | 4.448 | | | 4.007 | 6.054 | | 27.575 | | 4.953 | | 46.091 | | 21.274 | |
| Pbr006623.4 | hypothetical protein; K01580 glutamate decarboxylase [EC:4.1.1.15] | 186.313 | | | 295.45 | 233.011 | | 261.476 | | 427.07 | | 268.131 | | 193.235 | |
| Pbr007072.2 | pyruvate kinase, putative (EC:2.7.1.40); K00873 pyruvate kinase [EC:2.7.1.40] | 1.235 | | | 1.069 | 0.282 | | 0.386 | | 0.55 | | 0 | | 0.127 | |
| Pbr007150.1 | enolase, putative (EC:4.2.1.11); K01689 enolase [EC:4.2.1.11] | 15.444 | | | 22.439 | 7.744 | | 14.366 | | 30.269 | | 16.232 | | 16.208 | |
| Pbr007177.1 | hypothetical protein LOC100262181; K13648 alpha-1,4-galacturonosyltransferase [EC:2.4.1.43] | 9.019 | | | 4.408 | 3.661 | | 3.182 | | 5.779 | | 1.303 | | 2.786 | |
| Pbr007232.1 | hypothetical protein; K00012 UDPglucose 6-dehydrogenase [EC:1.1.1.22] | 22.98 | | | 7.613 | 9.433 | | 16.005 | | 26.142 | | 30.451 | | 11.397 | |
| Pbr007298.1 | malate dehydrogenase (EC:1.1.1.37) | 69.929 | | | 35.662 | 22.104 | | 42.422 | | 57.786 | | 25.356 | | 26.972 | |
| Pbr007409.1 | Polygalacturonase precursor, putative (EC:3.2.1.67); K01184 polygalacturonase [EC:3.2.1.15] | 63.875 | | | 2.004 | 0.141 | | 93.329 | | 4.265 | | 0.118 | | 0.127 | |
| Pbr007632.1 | Beta-fructofuranosidase, insoluble isoenzyme 3 precursor | 10.255 | | | 3.74 | 0.704 | | 0.386 | | 0.688 | | 0 | | 0.76 | |
| Pbr007764.1 | hypothetical protein; K01176 alpha-amylase [EC:3.2.1.1] | 1.359 | | | 0.801 | 0.141 | | 1.253 | | 0.138 | | 0 | | 0.253 | |
| Pbr007834.1 | isocitrate dehydrogenase (NAD+) (EC:1.1.1.41); K00030 isocitrate dehydrogenase (NAD+) [EC:1.1.1.41] | 2.471 | | | 1.736 | 1.971 | | 2.314 | | 3.715 | | 1.896 | | 1.266 | |
| Pbr007869.2 | phosphoglycerate/bisphosphoglycerate mutase family protein; K01834 phosphoglycerate mutase [EC:5.4.2.1] | 11.49 | | | 15.227 | 10.559 | | 11.763 | | 18.299 | | 8.294 | | 10.89 | |
| Pbr007870.2 | phosphoglycerate/bisphosphoglycerate mutase family protein; K01834 phosphoglycerate mutase [EC:5.4.2.1] | 7.413 | | | 5.209 | 2.957 | | 3.471 | | 9.081 | | 3.673 | | 2.659 | |
| Pbr008035.1 | sucrose phosphate syntase, putative (EC:2.4.1.14); K00696 sucrose-phosphate synthase [EC:2.4.1.14] | 2.842 | | | 7.88 | 10.7 | | 4.435 | | 11.97 | | 3.555 | | 3.546 | |
| Pbr008172.1 | hypothetical protein LOC100250604; K00889 1-phosphatidylinositol-4-phosphate 5-kinase [EC:2.7.1.68] | 17.915 | | | 21.371 | 11.686 | | 11.088 | | 20.225 | | 9.597 | | 5.825 | |
| Pbr008291.1 | hypothetical protein; K00434 L-ascorbate peroxidase [EC:1.11.1.11] | 152.213 | | | 148.526 | 223.296 | | 31.624 | | 19.262 | | 111.257 | | 122.576 | |
| Pbr008441.1 | alpha-galactosidase (EC:2.4.1.67); K07407 alpha-galactosidase [EC:3.2.1.22] | 19.891 | | | 39.135 | 19.007 | | 25.164 | | 57.099 | | 22.986 | | 16.082 | |
| Pbr008457.1 | hypothetical protein LOC100266759; K00695 sucrose synthase [EC:2.4.1.13] | 1.73 | | | 1.736 | 2.112 | | 1.735 | | 3.027 | | 0.711 | | 1.013 | |
| Pbr008464.1 | hypothetical protein LOC100266759; K00695 sucrose synthase [EC:2.4.1.13] | 1.606 | | | 1.603 | 1.971 | | 1.735 | | 3.027 | | 0.711 | | 1.013 | |
| Pbr008471.1 | utp-glucose-1-phosphate uridylyltransferase, putative (EC:2.7.7.9); K00963 UTP--glucose-1-phosphate | 161.726 | | | 204.625 | 292.566 | | 143.176 | | 155.473 | | 375.952 | | 267.819 | |
| Pbr008479.1 | hypothetical protein LOC100266215; K01580 glutamate decarboxylase [EC:4.1.1.15] | 139.611 | | | 188.062 | 79.266 | | 117.337 | | 183.678 | | 109.717 | | 108.141 | |
| Pbr008480.1 | hypothetical protein; K01580 glutamate decarboxylase [EC:4.1.1.15] | 146.777 | | | 192.203 | 84.334 | | 121 | | 185.605 | | 118.485 | | 117.258 | |
| Pbr008481.1 | hypothetical protein; K01580 glutamate decarboxylase [EC:4.1.1.15] | 73.635 | | | 113.532 | 32.101 | | 52.257 | | 104.704 | | 50.474 | | 56.729 | |
| Pbr008522.1 | hypothetical protein LOC100250604; K00889 1-phosphatidylinositol-4-phosphate 5-kinase [EC:2.7.1.68] | 10.131 | | | 9.35 | 4.646 | | 9.256 | | 13.208 | | 3.436 | | 3.292 | |
| Pbr008761.1 | phosphofructokinase, putative (EC:2.7.1.11); K00850 6-phosphofructokinase [EC:2.7.1.11] | 46.207 | | | 74.53 | 26.047 | | 20.633 | | 42.377 | | 54.621 | | 31.91 | |
| Pbr009063.1 | Glycosyltransferase QUASIMODO1, | 17.05 | | | 6.144 | 7.884 | | 5.399 | | 3.44 | | 2.844 | | 4.052 | |
| Pbr009268.1 | hypothetical protein LOC100267958; K12446 L-arabinokinase [EC:2.7.1.46] | 39.289 | | | 35.662 | 50.404 | | 58.62 | | 15.822 | | 16.943 | | 16.715 | |
| Pbr009290.1 | hypothetical protein LOC100253745; K12450 UDP-glucose 4,6-dehydratase [EC:4.2.1.76] | 0.371 | | | 2.938 | 0 | | 0.675 | | 1.238 | | 0.711 | | 0.127 | |
| Pbr009527.1 | phosphoinositide phospholipase C (EC:3.1.4.11); K05857 phospholipase C, delta [EC:3.1.4.11] | 10.625 | | | 10.418 | 4.083 | | 12.534 | | 9.769 | | 3.792 | | 6.711 | |
| Pbr009528.1 | 1-phosphatidylinositol-4,5-bisphosphate phosphodiesterase, putative (EC:3.1.4.11); K05857 phospholipase C, delta [EC:3.1.4.11] | 20.262 | | | 43.81 | 85.742 | | 38.277 | | 40.726 | | 89.93 | | 77.37 | |
| Pbr009578.1 | hypothetical protein; K00696 sucrose-phosphate synthase [EC:2.4.1.14] | 20.015 | | | 20.836 | 19.57 | | 16.39 | | 11.145 | | 6.161 | | 6.838 | |
| Pbr009623.1 | rbcL; ribulose-1,5-bisphosphate carboxylase/oxygenase large subunit (EC:4.1.1.39); K01601 ribulose-bisphosphate carboxylase large chain [EC:4.1.1.39] | 4.448 | | | 4.007 | 5.772 | | 27.189 | | 4.953 | | 45.143 | | 20.767 | |
| Pbr009764.1 | hypothetical protein; K01183 chitinase [EC:3.2.1.14] | 131.457 | | | 49.687 | 13.798 | | 74.432 | | 136.624 | | 4.739 | | 15.322 | |
| Pbr009765.1 | hypothetical protein; K01183 chitinase [EC:3.2.1.14] | 129.603 | | | 47.416 | 13.094 | | 72.793 | | 133.872 | | 4.384 | | 15.195 | |
| Pbr009785.1 | hypothetical protein; K01183 chitinase [EC:3.2.1.14] | 64.74 | | | 25.511 | 5.913 | | 34.227 | | 69.894 | | 2.607 | | 8.484 | |
| Pbr009786.1 | hypothetical protein; K01183 chitinase [EC:3.2.1.14] | 131.457 | | | 49.687 | 13.798 | | 74.432 | | 136.624 | | 4.739 | | 15.322 | |
| Pbr010008.1 | rbcL; ribulose-1,5-bisphosphate carboxylase/oxygenase large subunit (EC:4.1.1.39); K01601 ribulose-bisphosphate carboxylase large chain [EC:4.1.1.39] | 4.448 | | | 4.007 | 6.054 | | 27.575 | | 4.953 | | 46.091 | | 21.274 | |
| Pbr010442.1 | Pectinesterase-2 precursor, putative (EC:3.1.1.11); K01051 pectinesterase [EC:3.1.1.11] | 7.166 | | | 14.826 | 5.35 | | 2.025 | | 6.742 | | 8.057 | | 8.611 | |
| Pbr010451.1 | Pectinesterase-2 precursor, putative (EC:3.1.1.11); K01051 pectinesterase [EC:3.1.1.11] | 7.166 | | | 14.826 | 5.35 | | 2.025 | | 6.742 | | 8.057 | | 8.611 | |
| Pbr010484.1 | rbcL; ribulose-1,5-bisphosphate carboxylase/oxygenase large subunit (EC:4.1.1.39); K01601 ribulose-bisphosphate carboxylase large chain [EC:4.1.1.39] | 4.448 | | | 4.007 | 6.054 | | 27.575 | | 4.953 | | 46.091 | | 21.274 | |
| Pbr010532.1 | fructose-bisphosphatase (EC:3.1.3.11); K03841 fructose-1,6-bisphosphatase I [EC:3.1.3.11] | 33.482 | | | 58.502 | 57.865 | | 35.866 | | 23.665 | | 39.455 | | 39.888 | |
| Pbr010726.1 | hypothetical protein; K01728 pectate lyase [EC:4.2.2.2] | 15.691 | | | 40.204 | 18.021 | | 27.092 | | 65.079 | | 20.142 | | 17.095 | |
| Pbr010727.1 | hypothetical protein LOC100246124; K01728 pectate lyase | 10.749 | | | 25.645 | 8.025 | | 15.234 | | 34.809 | | 13.626 | | 8.484 | |
| Pbr010732.1 | hypothetical protein LOC100243846; K00627 pyruvate dehydrogenase E2 component (dihydrolipoamide acetyltransferase) [EC:2.3.1.12] | 22.239 | | | 27.915 | 20.133 | | 34.516 | | 47.467 | | 20.616 | | 24.566 | |
| Pbr010751.1 | hypothetical protein LOC100243846; K00627 pyruvate dehydrogenase E2 component (dihydrolipoamide acetyltransferase) [EC:2.3.1.12] | 22.239 | | | 27.915 | 20.133 | | 34.516 | | 47.467 | | 20.616 | | 24.566 | |
| Pbr010756.1 | Pectate lyase precursor, putative (EC:4.2.2.2) | 31.011 | | | 74.797 | 29.989 | | 48.111 | | 115.16 | | 38.626 | | 30.897 | |
| Pbr010784.1 | hypothetical protein; K00703 starch synthase [EC:2.4.1.21] | 11.984 | | | 9.216 | 13.657 | | 10.991 | | 3.164 | | 3.436 | | 2.786 | |
| Pbr010837.1 | UDP-glucuronate 5-epimerase, putative (EC:5.1.3.12); K08679 UDP-glucuronate 4-epimerase [EC:5.1.3.6] | 29.405 | | | 26.847 | 18.725 | | 8.581 | | 12.658 | | 19.076 | | 26.719 | |
| Pbr010991.1 | pyruvate kinase, putative (EC:2.7.1.40); K00873 pyruvate kinase [EC:2.7.1.40] | 0.618 | | | 1.603 | 0.282 | | 0.386 | | 1.238 | | 0.118 | | 0.38 | |
| Pbr011131.1 | Homomeric Acetyl-CoA Carboxylase (Hom-ACCase) (EC:6.4.1.2); K11262 acetyl-CoA carboxylase / biotin carboxylase [EC:6.4.1.2 6.3.4.14] | 21.498 | | | 37.799 | 31.115 | | 25.839 | | 48.981 | | 26.185 | | 20.894 | |
| Pbr011138.1 | phosphoglycerate mutase, putative (EC:5.4.2.1); K01834 phosphoglycerate mutase [EC:5.4.2.1] | 7.042 | | | 7.346 | 7.884 | | 5.399 | | 11.557 | | 12.796 | | 6.711 | |
| Pbr011169.1 | hypothetical protein LOC100247690; K01006 pyruvate,orthophosphate dikinase [EC:2.7.9.1] | 19.027 | | | 25.378 | 54.205 | | 18.126 | | 5.779 | | 15.166 | | 14.942 | |
| Pbr011170.1 | hypothetical protein LOC100247690; K01006 pyruvate,orthophosphate dikinase [EC:2.7.9.1] | 31.011 | | | 38.067 | 123.334 | | 61.995 | | 14.034 | | 19.668 | | 32.923 | |
| Pbr011171.1 | hypothetical protein LOC100247690; K01006 pyruvate,orthophosphate dikinase [EC:2.7.9.1] | 4.818 | | | 5.877 | 20.837 | | 9.545 | | 2.477 | | 4.028 | | 5.445 | |
| Pbr011300.1 | glycosyltransferase, CAZy family GT8 (EC:2.4.1.43); K13648 alpha-1,4-galacturonosyltransferase [EC:2.4.1.43] | 12.231 | | | 15.627 | 9.574 | | 7.038 | | 17.611 | | 8.175 | | 12.916 | |
| Pbr011400.1 | GDP-D-mannose pyrophosphorylase; K00971 mannose-1-phosphate guanylyltransferase [EC:2.7.7.22] | 110.453 | | | 164.421 | 120.799 | | 64.598 | | 115.986 | | 110.902 | | 118.397 | |
| Pbr011434.1 | malate dehydrogenase (EC:1.1.1.37); K00026 malate dehydrogenase [EC:1.1.1.37] | 154.807 | | | 65.581 | 33.931 | | 66.044 | | 84.616 | | 47.394 | | 45.713 | |
| Pbr011621.1 | hypothetical protein LOC100244966; K01103 6-phosphofructo-2-kinase / fructose-2,6-bisphosphatase [EC:2.7.1.105 3.1.3.46] | 29.405 | | | 38.467 | 36.887 | | 50.81 | | 38.387 | | 22.275 | | 28.365 | |
| Pbr011624.1 | PDC1; pyruvate decarboxylase 1 | 7.537 | | | 25.645 | 16.895 | | 7.617 | | 10.594 | | 32.82 | | 8.864 | |
| Pbr011696.1 | hypothetical protein LOC100267980; K00921 1-phosphatidylinositol-3-phosphate 5-kinase [EC:2.7.1.150] | 46.455 | | | 41.539 | 38.014 | | 39.819 | | 36.598 | | 13.152 | | 15.575 | |
| Pbr011702.1 | pectinesterase family protein (EC:3.1.1.11); K01051 pectinesterase [EC:3.1.1.11] | 0.865 | | | 2.538 | 0 | | 0.964 | | 5.916 | | 0.829 | | 0.253 | |
| Pbr011950.1 | hypothetical protein; K01728 pectate lyase [EC:4.2.2.2] | 26.934 | | | 38.334 | 18.303 | | 34.709 | | 65.904 | | 13.626 | | 10.89 | |
| Pbr011969.1 | hypothetical protein LOC100246616; K00382 dihydrolipoamide dehydrogenase [EC:1.8.1.4] | 25.698 | | | 39.536 | 17.177 | | 23.236 | | 55.723 | | 34.242 | | 23.679 | |
| Pbr012055.1 | l-lactate dehydrogenase, putative (EC:1.1.1.27); K00016 L-lactate dehydrogenase [EC:1.1.1.27] | 79.072 | | | 154.136 | 69.129 | | 41.073 | | 85.717 | | 117.774 | | 76.737 | |
| Pbr012169.1 | alpha-galactosidase (EC:2.4.1.67); K07407 alpha-galactosidase [EC:3.2.1.22] | 18.78 | | | 37.132 | 18.444 | | 25.164 | | 57.099 | | 22.986 | | 15.449 | |
| Pbr012208.1 | myo-inositol oxidase; K00469 inositol oxygenase [EC:1.13.99.1] | 1.359 | | | 1.069 | 2.253 | | 2.989 | | 2.614 | | 0.592 | | 0.886 | |
| Pbr012278.1 | alpha-galactosidase/alpha-n-acetylgalactosaminidase, putative (EC:3.2.1.22); K07407 alpha-galactosidase [EC:3.2.1.22] | 9.019 | | | 15.494 | 10.419 | | 8.388 | | 18.712 | | 12.204 | | 6.838 | |
| Pbr012370.1 | acetyl-CoA carboxylase (EC:6.4.1.2); K01962 acetyl-CoA carboxylase carboxyl transferase subunit alpha [EC:6.4.1.2] | 23.969 | | | 30.988 | 12.39 | | 21.597 | | 26.142 | | 20.379 | | 18.234 | |
| Pbr012392.1 | hypothetical protein LOC100240924; K03841 fructose-1,6-bisphosphatase I [EC:3.1.3.11] | 61.775 | | | 117.405 | 62.512 | | 77.903 | | 78.425 | | 71.802 | | 46.473 | |
| Pbr012401.1 | hypothetical protein LOC100240924; K03841 fructose-1,6-bisphosphatase I [EC:3.1.3.11] | 61.775 | | | 117.405 | 62.512 | | 77.903 | | 78.425 | | 71.802 | | 46.473 | |
| Pbr012442.1 | hypothetical protein LOC100255703; K01051 pectinesterase [EC:3.1.1.11] | 0.124 | | | 0 | 0 | | 1.061 | | 0.275 | | 0 | | 0 | |
| Pbr012553.1 | aldose 1-epimerase, putative; K01792 glucose-6-phosphate 1-epimerase [EC:5.1.3.15] | 35.088 | | | 16.162 | 14.502 | | 20.44 | | 21.326 | | 4.976 | | 12.789 | |
| Pbr012634.2 | hypothetical protein LOC100253896; K00963 UTP--glucose-1-phosphate uridylyltransferase [EC:2.7.7.9] | 14.332 | | | 17.364 | 23.371 | | 14.751 | | 14.722 | | 33.887 | | 23.553 | |
| Pbr012641.1 | hypothetical protein LOC100266759; K00695 sucrose synthase [EC:2.4.1.13] | 5.683 | | | 10.151 | 10.559 | | 10.606 | | 13.759 | | 4.621 | | 5.572 | |
| Pbr012642.1 | K00695 sucrose synthase [EC:2.4.1.13] | 1.853 | | | 1.736 | 12.812 | | 8.87 | | 7.705 | | 2.488 | | 1.646 | |
| Pbr012687.2 | hypothetical protein LOC100242517; K00850 6-phosphofructokinase [EC:2.7.1.11] | 0.371 | | | 1.202 | 2.957 | | 0.964 | | 1.789 | | 1.303 | | 1.013 | |
| Pbr013026.1 | PDC1; pyruvate decarboxylase 1; K01568 pyruvate decarboxylase [EC:4.1.1.1] | 4.448 | | | 14.425 | 8.729 | | 4.339 | | 5.091 | | 13.27 | | 5.318 | |
| Pbr013028.1 | hypothetical protein LOC100244966; K01103 6-phosphofructo-2-kinase / fructose-2,6-bisphosphatase [EC:2.7.1.105 3.1.3.46] | 33.976 | | | 48.084 | 53.923 | | 78.674 | | 50.632 | | 28.673 | | 38.495 | |
| Pbr013232.1 | hypothetical protein LOC100247335; K01595 phosphoenolpyruvate carboxylase [EC:4.1.1.31] | 13.467 | | | 13.223 | 8.87 | | 7.617 | | 7.98 | | 7.228 | | 5.698 | |
| Pbr013288.1 | hypothetical protein; K01187 alpha-glucosidase [EC:3.2.1.20] | 61.775 | | | 184.322 | 38.859 | | 111.841 | | 436.013 | | 141.352 | | 45.333 | |
| Pbr013299.1 | Ribulose bisphosphate carboxylase small chain 1B, chloroplast precursor, putative (EC:4.1.1.39); K01602 ribulose-bisphosphate carboxylase small chain [EC:4.1.1.39] | 200.274 | | | 230.804 | 194.856 | | 315.468 | | 197.987 | | 133.532 | | 200.274 | |
| Pbr013317.1 | mannose-6-phosphate isomerase, putative (EC:5.3.1.8); K01809 mannose-6-phosphate isomerase [EC:5.3.1.8] | 40.524 | | | 16.295 | 4.787 | | 13.498 | | 13.071 | | 5.45 | | 40.524 | |
| Pbr013399.1 | ribose-5-phosphate isomerase (EC:5.3.1.6); K01807 ribose 5-phosphate isomerase A [EC:5.3.1.6] | 198.05 | | | 83.88 | 23.935 | | 34.806 | | 33.158 | | 3.91 | | 198.05 | |
| Pbr013598.1 | hypothetical protein; K00703 starch synthase [EC:2.4.1.21] | 36.941 | | | 35.395 | 41.815 | | 28.249 | | 13.896 | | 17.773 | | 36.941 | |
| Pbr013637.1 | pyruvate kinase, putative (EC:2.7.1.40); K00873 pyruvate kinase [EC:2.7.1.40] | 30.023 | | | 39.536 | 20.556 | | 22.657 | | 44.853 | | 31.161 | | 30.023 | |
| Pbr013857.1 | hypothetical protein; K00697 alpha,alpha-trehalose-phosphate synthase (UDP-forming) [EC:2.4.1.15]; K01087 trehalose-phosphatase [EC:3.1.3.12] | 19.768 | | | 50.088 | 22.808 | | 27.189 | | 63.703 | | 36.375 | | 19.768 | |
| Pbr013861.1 | hypothetical protein LOC100242009; K00913 inositol-1,3,4-trisphosphate 5/6-kinase / inositol-tetrakisphosphate 1-kinase [EC:2.7.1.159 2.7.1.134] | 25.575 | | | 36.998 | 14.783 | | 12.92 | | 18.987 | | 11.611 | | 25.575 | |
| Pbr013894.1 | hypothetical protein LOC100243192; K01640 hydroxymethylglutaryl-CoA lyase [EC:4.1.3.4] | 2.224 | | | 5.61 | 4.365 | | 5.11 | | 3.99 | | 2.014 | | 2.224 | |
| Pbr013898.2 | phosphoglucomutase (EC:5.4.2.2); K01835 phosphoglucomutase [EC:5.4.2.2] | 18.285 | | | 38.334 | 25.765 | | 26.996 | | 28.068 | | 36.138 | | 18.285 | |
| Pbr013912.1 | sorbitol dehydrogenase, putative / L-iditol 2-dehydrogenase, putative (EC:1.1.1.14); K00008 L-iditol 2-dehydrogenase | 9.884 | | | 13.49 | 14.502 | | 60.066 | | 42.927 | | 28.91 | | 9.884 | |
| Pbr013913.1 | sorbitol dehydrogenase, putative / L-iditol 2-dehydrogenase, putative (EC:1.1.1.14); K00008 L-iditol 2-dehydrogenase [EC:1.1.1.14] | 177.788 | | | 73.462 | 73.916 | | 187.816 | | 174.873 | | 59.479 | | 177.788 | |
| Pbr013914.1 | sorbitol dehydrogenase, putative / L-iditol 2-dehydrogenase, putative (EC:1.1.1.14); K00008 L-iditol 2-dehydrogenase [EC:1.1.1.14] | 65.728 | | | 37.666 | 48.01 | | 69.804 | | 63.703 | | 32.465 | | 65.728 | |
| Pbr013915.1 | L-iditol 2-dehydrogenase (EC:1.1.1.14); K00008 L-iditol 2-dehydrogenase [EC:1.1.1.14] | 278.604 | | | 210.769 | 710.577 | | 463.176 | | 202.665 | | 388.985 | | 278.604 | |
| Pbr013916.1 | sorbitol dehydrogenase, putative / L-iditol 2-dehydrogenase, putative (EC:1.1.1.14); K00008 L-iditol 2-dehydrogenase [EC:1.1.1.14] | 25.451 | | | 19.234 | 61.667 | | 45.99 | | 20.088 | | 44.313 | | 25.451 | |
| Pbr013917.1 | L-iditol 2-dehydrogenase (EC:1.1.1.14); K00008 L-iditol 2-dehydrogenase [EC:1.1.1.14] | 12.602 | | | 10.685 | 15.909 | | 12.534 | | 6.467 | | 13.507 | | 12.602 | |
| Pbr014121.1 | aldose 1-epimerase, putative; K01792 glucose-6-phosphate 1-epimerase [EC:5.1.3.15] | 21.621 | | | 18.833 | 24.075 | | 25.743 | | 10.594 | | 23.46 | | 21.621 | |
| Pbr014325.1 | aldose 1-epimerase, putative; K01792 glucose-6-phosphate 1-epimerase [EC:5.1.3.15] | 13.343 | | | 19.367 | 11.545 | | 19.283 | | 24.766 | | 11.73 | | 13.343 | |
| Pbr014526.1 | aldose 1-epimerase, putative; K01792 glucose-6-phosphate 1-epimerase [EC:5.1.3.15] | 34.47 | | | 14.025 | 14.502 | | 19.283 | | 19.262 | | 5.213 | | 34.47 | |
| Pbr014652.1 | sorbitol dehydrogenase, putative / L-iditol 2-dehydrogenase, putative (EC:1.1.1.14); K00008 L-iditol 2-dehydrogenase [EC:1.1.1.14] | 7.413 | | | 8.148 | 9.011 | | 5.399 | | 4.128 | | 10.427 | | 7.413 | |
| Pbr014704.2 | pyruvate kinase (EC:2.7.1.40); K00873 pyruvate kinase [EC:2.7.1.40] | 20.262 | | | 30.587 | 15.206 | | 20.151 | | 38.387 | | 25.474 | | 20.262 | |
| Pbr014731.1 | hypothetical protein LOC100261773; K00849 galactokinase [EC:2.7.1.6] | 2.842 | | | 7.613 | 6.617 | | 3.76 | | 7.292 | | 12.441 | | 2.842 | |
| Pbr014773.1 | hypothetical protein LOC100247690; K01006 pyruvate,orthophosphate dikinase [EC:2.7.9.1] | 5.189 | | | 8.415 | 19.57 | | 9.449 | | 1.926 | | 3.91 | | 5.189 | |
| Pbr014774.1 | hypothetical protein LOC100247690; K01006 pyruvate,orthophosphate dikinase [EC:2.7.9.1] | 5.56 | | | 7.747 | 21.26 | | 8.195 | | 3.164 | | 3.792 | | 5.56 | |
| Pbr014902.1 | hypothetical protein LOC100243135; K00695 sucrose synthase [EC:2.4.1.13] | 2.347 | | | 6.278 | 10.982 | | 4.146 | | 3.164 | | 8.768 | | 2.347 | |
| Pbr014962.1 | Glycosyltransferase QUASIMODO1, putative; K13648 alpha-1,4-galacturonosyltransferase [EC:2.4.1.43] | 45.343 | | | 67.318 | 20.978 | | 35.866 | | 76.086 | | 33.413 | | 45.343 | |
| Pbr015010.1 | galactokinase, putative (EC:2.7.1.157); K00849 galactokinase [EC:2.7.1.6] | 62.392 | | | 48.351 | 51.671 | | 68.069 | | 55.998 | | 34.242 | | 62.392 | |
| Pbr015038.1 | ATP citrate (pro-S)-lyase (EC:2.3.3.8); K01648 ATP citrate (pro-S)-lyase [EC:2.3.3.8] | 489.503 | | | 523.449 | 297.353 | | 430.684 | | 930.913 | | 445.502 | | 489.503 | |
| Pbr015118.1 | pyrophosphate--fructose-6-phosphate 1-phosphotransferase; | 101.311 | | | 175.774 | 164.586 | | 106.635 | | 173.359 | | 252.135 | | 101.311 | |
| Pbr015141.1 | hypothetical protein LOC100267681; K00036 glucose-6-phosphate 1-dehydrogenase [EC:1.1.1.49] | 54.609 | | | 41.139 | 9.574 | | 10.316 | | 15.135 | | 5.687 | | 54.609 | |
| Pbr015353.1 | Beta-fructofuranosidase, insoluble isoenzyme 3 precursor, putative (EC:3.2.1.26); K01193 beta-fructofuranosidase [EC:3.2.1.26] | 0.371 | | | 1.069 | 0.704 | | 0.482 | | 0.275 | | 0.592 | | 0.371 | |
| Pbr015533.1 | hypothetical protein LOC100242358; K00844 hexokinase [EC:2.7.1.1] | 94.886 | | | 71.058 | 38.718 | | 80.506 | | 68.656 | | 29.147 | | 94.886 | |
| Pbr015536.1 | Pectinesterase PPE8B precursor, putative (EC:3.1.1.11); K01051 pectinesterase [EC:3.1.1.11] | 0.247 | | | 1.469 | 1.126 | | 0.578 | | 1.376 | | 2.844 | | 0.38 | |
| Pbr015554.1 | hypothetical protein LOC100254212; K00469 inositol oxygenase [EC:1.13.99.1] | 0.618 | | | 0.401 | 0.563 | | 2.121 | | 0.275 | | 0.118 | | 0.127 | |
| Pbr015640.1 | hypothetical protein LOC100251489; K00889 1-phosphatidylinositol-4-phosphate 5-kinase [EC:2.7.1.68] | 1.112 | | | 2.538 | 0.282 | | 0.386 | | 1.789 | | 0.237 | | 0 | |
| Pbr015988.1 | hypothetical protein LOC100254744; K05605 3-hydroxyisobutyryl-CoA hydrolase [EC:3.1.2.4] | 9.884 | | | 13.09 | 10.559 | | 6.942 | | 5.641 | | 12.441 | | 10.384 | |
| Pbr016062.1 | Homomeric Acetyl-CoA Carboxylase (Hom-ACCase) (EC:6.4.1.2); K11262 acetyl-CoA carboxylase / biotin carboxylase [EC:6.4.1.2 6.3.4.14] | 14.332 | | | 21.371 | 10.982 | | 13.787 | | 27.517 | | 11.375 | | 9.75 | |
| Pbr016096.1 | trehalose-6-phosphate synthase, putative (EC:2.4.1.15 3.1.3.12); K00697 alpha,alpha-trehalose-phosphate synthase (UDP-forming) [EC:2.4.1.15]; K01087 trehalose-phosphatase [EC:3.1.3.12] | 55.474 | | | 57.167 | 99.118 | | 108.659 | | 47.055 | | 67.892 | | 84.461 | |
| Pbr016098.1 | hypothetical protein; K01653 acetolactate synthase I/III small subunit [EC:2.2.1.6] | 10.872 | | | 26.313 | 13.094 | | 11.859 | | 19.4 | | 15.048 | | 20.387 | |
| Pbr016162.1 | pyruvate decarboxylase (EC:4.1.1.1) | 170.745 | | | 637.916 | 321.287 | | 91.787 | | 111.583 | | 503.678 | | 204.505 | |
| Pbr016213.1 | hypothetical protein LOC100264186; K00688 starch phosphorylase [EC:2.4.1.1] | 7.66 | | | 14.158 | 12.108 | | 6.942 | | 14.034 | | 18.365 | | 11.397 | |
| Pbr016294.2 | ribose-phosphate diphosphokinase (EC:2.7.6.1); K00948 ribose-phosphate pyrophosphokinase [EC:2.7.6.1] | 17.544 | | | 20.436 | 6.476 | | 9.256 | | 23.252 | | 13.27 | | 18.868 | |
| Pbr016374.1 | glycosyltransferase, family GT8 (EC:2.4.1.43); K13648 alpha-1,4-galacturonosyltransferase [EC:2.4.1.43] | 7.413 | | | 8.815 | 3.379 | | 5.206 | | 7.842 | | 5.095 | | 3.546 | |
| Pbr016480.1 | hypothetical protein LOC100241904; K08679 UDP-glucuronate 4-epimerase [EC:5.1.3.6] | 223.501 | | | 165.623 | 222.733 | | 181.452 | | 249.858 | | 110.902 | | 116.878 | |
| Pbr016566.1 | Glycosyltransferase QUASIMODO1, putative; K13648 alpha-1,4-galacturonosyltransferase [EC:2.4.1.43] | 40.401 | | | 50.355 | 36.606 | | 34.806 | | 61.914 | | 39.218 | | 29.884 | |
| Pbr016797.1 | fructose-bisphosphate aldolase, putative (EC:4.1.2.13); K01623 fructose-bisphosphate aldolase, class I [EC:4.1.2.13] | 391.034 | | | 767.476 | 387.6 | | 292.329 | | 515.263 | | 637.092 | | 422.812 | |
| Pbr016798.1 | fructose-bisphosphate aldolase, putative (EC:4.1.2.13); K01623 fructose-bisphosphate aldolase, class I [EC:4.1.2.13] | 21.992 | | | 31.522 | 36.606 | | 10.509 | | 16.648 | | 45.143 | | 32.29 | |
| Pbr016849.1 | hypothetical protein LOC100263819; K00326 cytochrome-b5 reductase [EC:1.6.2.2] | 74.624 | | | 174.438 | 224.282 | | 61.898 | | 121.489 | | 310.311 | | 177.786 | |
| Pbr017155.1 | hypothetical protein LOC100265765; K00888 phosphatidylinositol 4-kinase [EC:2.7.1.67] | 58.439 | | | 86.017 | 66.876 | | 65.08 | | 106.492 | | 64.219 | | 41.281 | |
| Pbr017459.1 | phosphoenolpyruvate carboxykinase (ATP) (EC:4.1.1.49); K01610 phosphoenolpyruvate carboxykinase (ATP) [EC:4.1.1.49] | 19.027 | | | 51.156 | 39.844 | | 15.426 | | 28.756 | | 40.877 | | 33.683 | |
| Pbr017545.1 | hypothetical protein LOC100254950; K01689 enolase [EC:4.2.1.11] | 32.37 | | | 96.302 | 112.634 | | 33.649 | | 89.982 | | 219.315 | | 128.274 | |
| Pbr017584.1 | acetyl-CoA C-acetyltransferase (EC:2.3.1.9); K00626 acetyl-CoA C-acetyltransferase [EC:2.3.1.9] | 62.269 | | | 49.82 | 74.761 | | 80.121 | | 30.132 | | 20.379 | | 34.443 | |
| Pbr017718.1 | Phospholipase C 4 precursor, putative (EC:3.1.4.3) | 39.289 | | | 46.348 | 17.317 | | 77.903 | | 139.513 | | 59.242 | | 26.465 | |
| Pbr017947.1 | hypothetical protein LOC100261093; K00122 formate dehydrogenase [EC:1.2.1.2] | 1100.084 | | | 614.007 | 347.474 | | 1336.40 | | 320.99 | | 174.054 | | 783.196 | |
| Pbr017964.1 | hypothetical protein LOC100267896; K08232 monodehydroascorbate reductase (NADH) [EC:1.6.5.4] | 37.683 | | | 36.731 | 48.01 | | 47.532 | | 20.913 | | 16.351 | | 25.452 | |
| Pbr017969.1 | Polygalacturonase precursor, putative (EC:3.2.1.67); K01184 polygalacturonase [EC:3.2.1.15] | 0 | | | 0.801 | 0 | | 2.603 | | 2.339 | | 0.829 | | 0.127 | |
| Pbr017975.1 | isocitrate dehydrogenase, putative / NADP+ isocitrate dehydrogenase, putative; K00031 isocitrate dehydrogenase [EC:1.1.1.42] | 16.185 | | | 9.75 | 10.419 | | 18.126 | | 10.869 | | 7.109 | | 8.737 | |
| Pbr018101.1 | aconitase, putative (EC:4.2.1.3); K01681 aconitate hydratase 1 [EC:4.2.1.3] | 467.017 | | | 284.498 | 223.718 | | 314.311 | | 221.79 | | 133.177 | | 160.311 | |
| Pbr018706.1 | pyruvate kinase, putative (EC:2.7.1.40); K00873 pyruvate kinase [EC:2.7.1.40] | 30.517 | | | 38.868 | 19.711 | | 21.597 | | 45.541 | | 30.688 | | 26.845 | |
| Pbr018739.1 | UDP-glucuronate 5-epimerase, putative (EC:5.1.3.12); K08679 UDP-glucuronate 4-epimerase [EC:5.1.3.6] | 65.975 | | | 69.722 | 38.577 | | 18.319 | | 28.893 | | 41.233 | | 56.856 | |
| Pbr018758.1 | hypothetical protein LOC100267958; K12446 L-arabinokinase [EC:2.7.1.46] | 137.14 | | | 90.959 | 181.058 | | 214.619 | | 25.591 | | 21.564 | | 47.232 | |
| Pbr018792.1 | fucose synthetase, putative (EC:1.1.1.271); K02377 GDP-L-fucose synthase [EC:1.1.1.271] | 34.223 | | | 50.488 | 28.158 | | 38.469 | | 60.813 | | 37.678 | | 37.735 | |
| Pbr018801.2 | hypothetical protein LOC100242115; K00847 fructokinase [EC:2.7.1.4] | 50.285 | | | 53.16 | 48.01 | | 77.421 | | 82.827 | | 37.204 | | 38.495 | |
| Pbr018870.1 | pyruvate kinase (EC:2.7.1.40); K00873 pyruvate kinase [EC:2.7.1.40] | 17.173 | | | 25.378 | 12.108 | | 17.065 | | 28.205 | | 22.394 | | 19.248 | |
| Pbr018896.1 | hypothetical protein LOC100265765; K00888 phosphatidylinositol 4-kinase [EC:2.7.1.67] | 53.497 | | | 78.671 | 58.851 | | 60.645 | | 96.448 | | 56.873 | | 37.735 | |
| Pbr019019.1 | Enoyl-CoA hydratase, mitochondrial precursor | 16.679 | | | 19.634 | 11.404 | | 13.402 | | 28.343 | | 13.152 | | 16.679 | |
| Pbr019113.1 | hypothetical protein; K01648 ATP citrate (pro-S)-lyase [EC:2.3.3.8] | 1.606 | | | 2.938 | 2.393 | | 2.121 | | 6.191 | | 3.91 | | 1.606 | |
| Pbr019116.1 | malic enzyme, putative (EC:1.1.1.40); K00029 malate dehydrogenase (oxaloacetate-decarboxylating)(NADP+) [EC:1.1.1.40] | 165.927 | | | 204.09 | 170.64 | | 198.228 | | 190.007 | | 94.669 | | 165.927 | |
| Pbr019272.1 | similar to starch synthase; K00703 starch synthase [EC:2.4.1.21] | 20.015 | | | 38.467 | 28.158 | | 28.057 | | 35.91 | | 29.977 | | 20.015 | |
| Pbr019445.1 | pyruvate decarboxylase, putative (EC:4.1.1.1); K01568 pyruvate decarboxylase [EC:4.1.1.1] | 111.318 | | | 464.012 | 288.342 | | 107.984 | | 164.691 | | 506.048 | | 111.318 | |
| Pbr019447.1 | pyruvate decarboxylase (EC:4.1.1.1); K01568 pyruvate decarboxylase [EC:4.1.1.1] | 0.494 | | | 3.072 | 0.845 | | 0.193 | | 0.55 | | 2.844 | | 0.494 | |
| Pbr019634.1 | hypothetical protein LOC100250317; K01176 alpha-amylase | 20.633 | | | 14.959 | 14.924 | | 16.39 | | 9.631 | | 11.375 | | 20.633 | |
| Pbr019918.1 | AtGH9A4 (Arabidopsis thaliana Glycosyl Hydrolase 9A4); catalytic/ hydrolase, hydrolyzing O-glycosyl compounds (EC:3.2.1.4); K01179 endoglucanase [EC:3.2.1.4] | 51.026 | | | 4.007 | 5.913 | | 8.581 | | 8.668 | | 2.725 | | 51.026 | |
| Pbr020194.1 | Glycosyltransferase QUASIMODO1, putative; K13648 alpha-1,4-galacturonosyltransferase [EC:2.4.1.43] | 10.008 | | | 6.812 | 2.957 | | 4.146 | | 7.292 | | 1.303 | | 10.008 | |
| Pbr020223.1 | enolase, putative (EC:4.2.1.11); K01689 enolase [EC:4.2.1.11] | 22.239 | | | 24.977 | 14.783 | | 21.211 | | 42.927 | | 40.166 | | 22.239 | |
| Pbr020361.1 | hypothetical protein LOC100255914; K05350 beta-glucosidase [EC:3.2.1.21] | 16.926 | | | 31.522 | 9.574 | | 21.404 | | 60.951 | | 13.744 | | 16.926 | |
| Pbr020464.1 | hypothetical protein LOC100260586; K12450 UDP-glucose 4,6-dehydratase [EC:4.2.1.76] | 297.507 | | | 212.906 | 143.326 | | 143.368 | | 239.951 | | 181.874 | | 297.507 | |
| Pbr020465.2 | trehalose-6-phosphate synthase; K00697 alpha,alpha-trehalose-phosphate synthase (UDP-forming) [EC:2.4.1.15] | 10.131 | | | 13.757 | 17.74 | | 14.848 | | 26.829 | | 12.441 | | 10.131 | |
| Pbr020588.1 | similar to APX6 (ASCORBATE PEROXIDASE 6); L-ascorbate peroxidase; K00434 L-ascorbate peroxidase [EC:1.11.1.11] | 1.606 | | | 3.74 | 1.83 | | 1.35 | | 2.614 | | 1.54 | | 1.606 | |
| Pbr020590.1 | similar to APX6 (ASCORBATE PEROXIDASE 6); L-ascorbate peroxidase; K00434 L-ascorbate peroxidase [EC:1.11.1.11] | 1.606 | | | 3.74 | 1.83 | | 1.35 | | 2.614 | | 1.54 | | 1.606 | |
| Pbr020612.1 | hypothetical protein; K01807 ribose 5-phosphate isomerase A [EC:5.3.1.6] | 2.224 | | | 3.473 | 1.408 | | 2.025 | | 2.752 | | 3.081 | | 2.224 | |
| Pbr020637.1 | similar to aldehyde dehydrogenase family 7 member A1; K14085 aldehyde dehydrogenase family 7 member A1 [EC:1.2.1.31 1.2.1.8 1.2.1.3] | 35.088 | | | 22.039 | 25.202 | | 47.436 | | 9.218 | | 10.901 | | 35.088 | |
| Pbr020713.1 | phosphatidylinositol synthase, putative (EC:2.7.8.11); K00999 CDP-diacylglycerol--inositol 3-phosphatidyltransferase [EC:2.7.8.11] | 19.891 | | | 16.028 | 18.162 | | 18.03 | | 22.977 | | 11.611 | | 19.891 | |
| Pbr020778.1 | Pectate lyase precursor, putative (EC:4.2.2.2); K01728 pectate lyase [EC:4.2.2.2] | 2.1 | | | 14.826 | 1.408 | | 8.292 | | 37.974 | | 1.896 | | 2.1 | |
| Pbr020798.1 | hypothetical protein LOC100248352; K01803 triosephosphate isomerase (TIM) [EC:5.3.1.1] | 2.842 | | | 6.278 | 6.899 | | 4.917 | | 6.742 | | 8.057 | | 2.842 | |
| Pbr020977.1 | hypothetical protein; K01728 pectate lyase [EC:4.2.2.2] | 6.548 | | | 10.952 | 6.195 | | 6.171 | | 17.198 | | 10.545 | | 6.548 | |
| Pbr021042.1 | hypothetical protein LOC100243178; K01792 glucose-6-phosphate 1-epimerase [EC:5.1.3.15] | 1.112 | | | 3.606 | 0.282 | | 1.832 | | 3.027 | | 2.251 | | 1.112 | |
| Pbr021157.1 | hypothetical protein; K01728 pectate lyase [EC:4.2.2.2] | 19.15 | | | 15.494 | 15.206 | | 22.657 | | 30.682 | | 9.36 | | 19.15 | |
| Pbr021220.1 | hypothetical protein; K00001 alcohol dehydrogenase [EC:1.1.1.1] | 15.32 | | | 72.527 | 33.508 | | 3.567 | | 5.366 | | 39.218 | | 15.32 | |
| Pbr021222.2 | alcohol dehydrogenase, putative (EC:1.1.1.284); K00001 alcohol dehydrogenase [EC:1.1.1.1] | 17.544 | | | 49.82 | 69.551 | | 13.884 | | 21.601 | | 58.531 | | 17.544 | |
| Pbr021310.1 | hypothetical protein LOC100267188; K00705 4-alpha-glucanotransferase [EC:2.4.1.25] | 39.783 | | | 39.402 | 43.223 | | 57.559 | | 37.424 | | 24.408 | | 39.783 | |
| Pbr021374.1 | hypothetical protein LOC100240835; K01807 ribose 5-phosphate isomerase A [EC:5.3.1.6] | 1.853 | | | 6.678 | 2.675 | | 1.543 | | 3.715 | | 2.962 | | 1.853 | |
| Pbr021378.1 | hypothetical protein; K01187 alpha-glucosidase [EC:3.2.1.20] | 15.814 | | | 28.45 | 11.967 | | 32.202 | | 51.87 | | 17.18 | | 15.814 | |
| Pbr021462.2 | hypothetical protein LOC100256047; K00972 UDP-N-acetylglucosamine pyrophosphorylase [EC:2.7.7.23] | 12.973 | | | 17.097 | 26.328 | | 16.487 | | 13.208 | | 34.479 | | 12.973 | |
| Pbr021488.1 | UDP-n-acteylglucosamine pyrophosphorylase, putative; K12447 UDP-sugar pyrophosphorylase [EC:2.7.7.64] | 27.675 | | | 27.248 | 20.837 | | 22.657 | | 19.262 | | 22.275 | | 12.916 | |
| Pbr021759.1 | hypothetical protein LOC100254814; K12451 3,5-epimerase/4-reductase [EC:5.1.3.- 1.1.1.-] | 65.481 | | | 98.038 | 100.103 | | 75.493 | | 119.15 | | 169.67 | | 109.154 | |
| Pbr021771.1 | hydroxymethylglutaryl-CoA synthase, putative (EC:2.3.3.10); K01641 hydroxymethylglutaryl-CoA synthase [EC:2.3.3.10] | 426.122 | | | 315.218 | 137.131 | | 238.048 | | 624.782 | | 145.381 | | 207.544 | |
| Pbr021808.1 | Beta-fructofuranosidase, insoluble isoenzyme 3 precursor, putative (EC:3.2.1.26); K01193 beta-fructofuranosidase [EC:3.2.1.26] | 10.131 | | | 3.473 | 0.704 | | 0.386 | | 0.688 | | 0 | | 0.886 | |
| Pbr021809.1 | Beta-fructofuranosidase, insoluble isoenzyme 3 precursor, putative (EC:3.2.1.26); K01193 beta-fructofuranosidase [EC:3.2.1.26] | 10.131 | | | 3.473 | 0.704 | | 0.386 | | 0.688 | | 0 | | 0.886 | |
| Pbr021846.1 | Homomeric Acetyl-CoA Carboxylase (Hom-ACCase) (EC:6.4.1.2); K11262 acetyl-CoA carboxylase / biotin carboxylase [EC:6.4.1.2 6.3.4.14] | 21.374 | | | 37.532 | 31.256 | | 25.936 | | 49.119 | | 26.422 | | 20.894 | |
| Pbr022043.1 | L-idonate dehydrogenase; K00008 L-iditol 2-dehydrogenase [EC:1.1.1.14] | 183.842 | | | 312.547 | 610.896 | | 334.848 | | 239.126 | | 663.514 | | 1175.997 | |
| Pbr022124.1 | hypothetical protein LOC100264358; K01711 GDPmannose 4,6-dehydratase [EC:4.2.1.47] | 354.958 | | | 271.007 | 67.158 | | 148.189 | | 244.354 | | 157.585 | | 103.455 | |
| Pbr022147.1 | hypothetical protein LOC100247106 | 4.818 | | | 11.887 | 3.097 | | 8.388 | | 5.916 | | 4.265 | | 3.546 | |
| Pbr022225.1 | Glycosyltransferase QUASIMODO1, putative; K13648 alpha-1,4-galacturonosyltransferase [EC:2.4.1.43] | 36.324 | | | 41.005 | 37.31 | | 33.938 | | 49.394 | | 26.541 | | 18.488 | |
| Pbr022277.1 | (S)-2-hydroxy-acid oxidase, putative (EC:1.1.3.15); K11517 (S)-2-hydroxy-acid oxidase [EC:1.1.3.15] | 6.301 | | | 11.086 | 9.433 | | 6.749 | | 13.208 | | 16.469 | | 12.03 | |
| Pbr022356.1 | hypothetical protein LOC100260645; K01179 endoglucanase [EC:3.2.1.4] | 1.235 | | | 6.144 | 1.971 | | 1.735 | | 5.503 | | 5.45 | | 1.773 | |
| Pbr022360.1 | fructose-bisphosphate aldolase, putative (EC:4.1.2.13); K01623 fructose-bisphosphate aldolase, class I [EC:4.1.2.13] | 323.329 | | | 629.1 | 384.643 | | 282.495 | | 362.129 | | 318.605 | | 364.056 | |
| Pbr022455.1 | hypothetical protein LOC100259893; K01792 glucose-6-phosphate 1-epimerase [EC:5.1.3.15] | 3.583 | | | 7.613 | 6.054 | | 4.917 | | 3.44 | | 5.924 | | 3.166 | |
| Pbr022520.1 | malate dehydrogenase, putative (EC:1.1.1.37); K00026 malate dehydrogenase [EC:1.1.1.37] | 29.281 | | | 73.061 | 44.913 | | 19.572 | | 15.96 | | 32.109 | | 47.106 | |
| Pbr022525.1 | malate dehydrogenase, putative (EC:1.1.1.37); K00026 malate dehydrogenase [EC:1.1.1.37] | 29.281 | | | 73.061 | 44.913 | | 19.572 | | 15.96 | | 32.109 | | 47.106 | |
| Pbr022545.1 | hypothetical protein LOC100255189; K01648 ATP citrate (pro-S)-lyase [EC:2.3.3.8] | 88.461 | | | 61.574 | 42.66 | | 48.4 | | 122.04 | | 61.257 | | 64.074 | |
| Pbr022559.1 | phosphoglycerate mutase, putative (EC:5.4.2.1); K01834 phosphoglycerate mutase [EC:5.4.2.1] | 4.201 | | | 10.151 | 7.04 | | 4.242 | | 7.155 | | 8.175 | | 6.458 | |
| Pbr022705.1 | aspartate ammonia-lyase (EC:4.2.1.2); K01679 fumarate hydratase, class II [EC:4.2.1.2] | 22.98 | | | 12.155 | 10.7 | | 21.79 | | 17.474 | | 13.626 | | 10.257 | |
| Pbr022713.1 | hypothetical protein; K01213 galacturan 1,4-alpha-galacturonidase [EC:3.2.1.67] | 4.942 | | | 2.137 | 0.845 | | 0 | | 0.138 | | 0.118 | | 0.127 | |
| Pbr022714.1 | SUS3; SUS3 (sucrose synthase 3); UDP-glycosyltransferase/ sucrose synthase/ transferase, transferring glycosyl groups (EC:2.4.1.13); K00695 sucrose synthase [EC:2.4.1.13] | 23.474 | | | 47.817 | 63.779 | | 27.189 | | 13.208 | | 56.754 | | 55.337 | |
| Pbr022715.1 | hypothetical protein LOC100267606; sucrose synthase | 170.004 | | | 299.19 | 463.628 | | 193.504 | | 86.817 | | 379.98 | | 423.572 | |
| Pbr022719.1 | Pectinesterase-2 precursor, putative (EC:3.1.1.11); K01051 pectinesterase [EC:3.1.1.11] | 9.019 | | | 2.137 | 0.282 | | 2.41 | | 1.651 | | 0 | | 0 | |
| Pbr023011.1 | hypothetical protein LOC100248915; K00128 aldehyde dehydrogenase (NAD+) [EC:1.2.1.3] | 2.1 | | | 1.469 | 4.365 | | 2.025 | | 3.027 | | 2.133 | | 1.52 | |
| Pbr023311.1 | L-ascorbate peroxidase 1, cytosolic, putative (EC:1.11.1.11); K00434 L-ascorbate peroxidase [EC:1.11.1.11] | 2.1 | | | 1.469 | 8.166 | | 5.592 | | 3.99 | | 2.962 | | 1.646 | |
| Pbr023529.2 | hypothetical protein; K01455 formamidase [EC:3.5.1.49] | 53.25 | | | 71.191 | 54.486 | | 132.666 | | 82.69 | | 57.465 | | 63.567 | |
| Pbr023612.1 | hypothetical protein LOC100256536; K01602 ribulose-bisphosphate carboxylase small chain [EC:4.1.1.39] | 15.197 | | | 18.566 | 22.104 | | 8.099 | | 17.198 | | 31.517 | | 32.037 | |
| Pbr024016.1 | hypothetical protein; K01728 pectate lyase [EC:4.2.2.2] | 99.952 | | | 31.789 | 42.097 | | 188.587 | | 81.176 | | 115.878 | | 45.333 | |
| Pbr024227.1 | Phosphoenolpyruvate carboxylase, putative (EC:1.3.1.74); K01595 phosphoenolpyruvate carboxylase [EC:4.1.1.31] | 584.018 | | | 327.105 | 250.61 | | 295.607 | | 766.221 | | 400.123 | | 396.727 | |
| Pbr024394.1 | formate dehydrogenase, putative (EC:1.2.1.2); K00122 formate dehydrogenase [EC:1.2.1.2] | 217.323 | | | 157.075 | 97.146 | | 298.596 | | 162.49 | | 84.361 | | 198.173 | |
| Pbr024419.1 | hypothetical protein LOC100267896; K08232 monodehydroascorbate reductase (NADH) [EC:1.6.5.4] | 15.197 | | | 23.775 | 33.649 | | 29.503 | | 14.309 | | 11.256 | | 17.728 | |
| Pbr024431.1 | NADP-specific isocitrate dehydrogenase, putative (EC:1.1.1.42); K00031 isocitrate dehydrogenase [EC:1.1.1.42] | 22.486 | | | 12.021 | 12.953 | | 26.996 | | 10.869 | | 7.938 | | 10.13 | |
| Pbr024496.1 | hypothetical protein LOC100258719; K05305 fucokinase [EC:2.7.1.52] | 13.714 | | | 29.118 | 16.895 | | 23.814 | | 43.615 | | 25.237 | | 18.361 | |
| Pbr024514.1 | hypothetical protein; K00888 phosphatidylinositol 4-kinase [EC:2.7.1.67] | 10.749 | | | 16.028 | 11.827 | | 14.269 | | 11.557 | | 6.991 | | 6.965 | |
| Pbr024600.1 | 3-isopropylmalate dehydratase, putative; K01704 3-isopropylmalate/(R)-2-methylmalate dehydratase small subunit [EC:4.2.1.33 4.2.1.35] | 164.939 | | | 133.3 | 80.251 | | 94.197 | | 126.717 | | 67.181 | | 129.034 | |
| Pbr024632.1 | ATP-citrate synthase, putative (EC:6.2.1.5) | 48.308 | | | 32.991 | 26.187 | | 28.442 | | 63.29 | | 30.095 | | 34.823 | |
| Pbr024640.1 | hypothetical protein LOC100241717; K00033 6-phosphogluconate dehydrogenase [EC:1.1.1.44] | 181.494 | | | 100.977 | 60.963 | | 59.584 | | 80.076 | | 44.432 | | 53.817 | |
| Pbr024742.1 | Pectinesterase-2 precursor, putative (EC:3.1.1.11); K01051 pectinesterase [EC:3.1.1.11] | 1.483 | | | 3.473 | 0.422 | | 0.868 | | 2.064 | | 0.829 | | 2.026 | |
| Pbr025297.1 | Glycosyltransferase QUASIMODO1, putative; K13648 alpha-1,4-galacturonosyltransferase [EC:2.4.1.43] | 13.096 | | | 8.949 | 7.884 | | 6.556 | | 8.393 | | 5.687 | | 4.179 | |
| Pbr025653.2 | hypothetical protein; K00913 inositol-1,3,4-trisphosphate 5/6-kinase / inositol-tetrakisphosphate 1-kinase [EC:2.7.1.159 2.7.1.134] | 1.112 | | | 3.74 | 2.675 | | 2.121 | | 0.963 | | 1.896 | | 1.899 | |
| Pbr025955.1 | hypothetical protein LOC100267838; K00913 inositol-1,3,4-trisphosphate 5/6-kinase / inositol-tetrakisphosphate 1-kinase [EC:2.7.1.159 2.7.1.134] | 6.054 | | | 7.346 | 6.054 | | 17.065 | | 21.326 | | 13.152 | | 4.812 | |
| Pbr026333.1 | Pectinesterase-2 precursor, putative (EC:3.1.1.11); K01051 pectinesterase [EC:3.1.1.11] | 17.05 | | | 5.209 | 0.141 | | 3.953 | | 5.916 | | 0.474 | | 0.38 | |
| Pbr026334.1 | Pectinesterase-2 precursor, putative (EC:3.1.1.11); K01051 pectinesterase [EC:3.1.1.11] | 2.718 | | | 0.267 | 0 | | 2.025 | | 0.55 | | 0 | | 0 | |
| Pbr026336.1 | hypothetical protein LOC100267606; K00695 sucrose synthase [EC:2.4.1.13] | 166.298 | | | 292.779 | 454.335 | | 191.19 | | 85.304 | | 371.331 | | 416.481 | |
| Pbr026337.1 | hypothetical protein LOC100267606; K00695 sucrose synthase [EC:2.4.1.13] | 9.39 | | | 20.436 | 34.212 | | 10.895 | | 5.228 | | 28.318 | | 27.225 | |
| Pbr026338.1 | SUS3; SUS3 (sucrose synthase 3); UDP-glycosyltransferase/ sucrose synthase/ transferase, transferring glycosyl groups (EC:2.4.1.13); K00695 sucrose synthase [EC:2.4.1.13] | 23.474 | | | 47.817 | 63.779 | | 27.189 | | 13.208 | | 56.754 | | 55.337 | |
| Pbr026339.1 | hypothetical protein; K01213 galacturan 1,4-alpha-galacturonidase [EC:3.2.1.67] | 4.942 | | | 2.137 | 0.845 | | 0 | | 0 | | 0.118 | | 0.127 | |
| Pbr026340.1 | hypothetical protein; K01213 galacturan | 4.942 | | | 2.137 | 0.845 | | 0 | | 0.138 | | 0.118 | | 0.127 | |
| Pbr026341.1 | SUS3; SUS3 (sucrose synthase 3); UDP-glycosyltransferase/ sucrose synthase/ transferase, transferring glycosyl groups (EC:2.4.1.13); K00695 sucrose synthase [EC:2.4.1.13] | 23.474 | | | 47.817 | 63.779 | | 27.189 | | 13.208 | | 56.754 | | 55.337 | |
| Pbr026342.1 | hypothetical protein LOC100267606; K00695 sucrose synthase [EC:2.4.1.13] | 166.298 | | | 292.779 | 454.335 | | 191.19 | | 85.304 | | 371.331 | | 416.481 | |
| Pbr026344.1 | Pectinesterase-2 precursor, putative (EC:3.1.1.11); K01051 pectinesterase [EC:3.1.1.11] | 2.718 | | | 0.267 | 0 | | 2.025 | | 0.55 | | 0 | | 0 | |
| Pbr026345.1 | Pectinesterase-2 precursor, putative (EC:3.1.1.11); K01051 pectinesterase [EC:3.1.1.11] | 18.038 | | | 5.476 | 0.141 | | 4.049 | | 6.054 | | 0.355 | | 0.507 | |
| Pbr026452.2 | hypothetical protein LOC100251825; K12373 hexosaminidase [EC:3.2.1.52] | 81.419 | | | 74.664 | 59.837 | | 74.914 | | 67.418 | | 47.275 | | 37.355 | |
| Pbr026466.1 | hypothetical protein LOC100246761; K01728 pectate lyase [EC:4.2.2.2] | 10.996 | | | 13.49 | 12.812 | | 10.606 | | 8.255 | | 11.967 | | 18.108 | |
| Pbr026495.1 | hypothetical protein; K00889 1-phosphatidylinositol-4-phosphate 5-kinase [EC:2.7.1.68] | 4.448 | | | 2.671 | 2.675 | | 2.507 | | 3.302 | | 1.896 | | 1.013 | |
| Pbr026498.1 | hypothetical protein LOC100258261; K00889 1-phosphatidylinositol-4-phosphate 5-kinase [EC:2.7.1.68] | 23.104 | | | 24.443 | 20.133 | | 22.947 | | 25.178 | | 11.493 | | 8.231 | |
| Pbr026674.1 | K01900 succinyl-CoA synthetase beta subunit | 48.802 | | | 62.643 | 34.072 | | 58.716 | | 83.928 | | 63.271 | | 46.852 | |
| Pbr026722.1 | aldose 1-epimerase, putative; K01792 glucose-6-phosphate 1-epimerase [EC:5.1.3.15] | 4.571 | | | 3.206 | 5.209 | | 4.435 | | 1.926 | | 4.265 | | 1.899 | |
| Pbr026727.1 | hypothetical protein LOC100241717; K00033 6-phosphogluconate dehydrogenase [EC:1.1.1.44] | 239.933 | | | 154.003 | 74.479 | | 89.955 | | 140.201 | | 72.987 | | 81.422 | |
| Pbr026777.1 | ATP citrate (pro-S)-lyase (EC:2.3.3.8); K01648 ATP citrate (pro-S)-lyase [EC:2.3.3.8] | 493.086 | | | 518.908 | 294.255 | | 430.009 | | 914.677 | | 443.607 | | 423.825 | |
| Pbr026918.3 | hypothetical protein; K01961 acetyl-CoA carboxylase | 18.903 | | | 27.915 | 11.123 | | 19.476 | | 34.259 | | 16.469 | | 12.41 | |
| Pbr027025.1 | NADP-specific isocitrate dehydrogenase, putative (EC:1.1.1.42); K00031 isocitrate dehydrogenase [EC:1.1.1.42] | 12.478 | | | 7.88 | 7.462 | | 14.944 | | 7.705 | | 5.687 | | 7.471 | |
| Pbr027032.1 | Polygalacturonase precursor, putative (EC:3.2.1.67); K01184 polygalacturonase [EC:3.2.1.15] | 0 | | | 0.801 | 0 | | 2.603 | | 2.339 | | 0.829 | | 0.127 | |
| Pbr027037.1 | hypothetical protein LOC100267896; K08232 monodehydroascorbate reductase (NADH) [EC:1.6.5.4] | 37.683 | | | 36.731 | 48.01 | | 47.532 | | 20.913 | | 16.351 | | 25.452 | |
| Pbr027064.1 | rbcL; ribulose-1,5-bisphosphate carboxylase/oxygenase large subunit (EC:4.1.1.39); K01601 ribulose-bisphosphate carboxylase large chain [EC:4.1.1.39] | 4.448 | | | 4.007 | 6.054 | | 27.575 | | 4.953 | | 46.091 | | 21.274 | |
| Pbr027083.1 | rbcL; ribulose-1,5-bisphosphate carboxylase/oxygenase large subunit (EC:4.1.1.39); K01601 ribulose-bisphosphate carboxylase large chain [EC:4.1.1.39] | 4.448 | | | 4.007 | 6.054 | | 27.575 | | 4.953 | | 46.091 | | 21.274 | |
| Pbr027358.1 | hypothetical protein LOC100264056; K13648 alpha-1,4-galacturonosyltransferase [EC:2.4.1.43] | 0.124 | | | 4.274 | 276.375 | | 42.519 | | 11.695 | | 221.685 | | 171.835 | |
| Pbr027508.1 | hypothetical protein LOC100247335; K01595 phosphoenolpyruvate carboxylase [EC:4.1.1.31] | 6.548 | | | 7.88 | 4.224 | | 6.171 | | 9.493 | | 2.844 | | 2.153 | |
| Pbr027517.1 | (S)-2-hydroxy-acid oxidase, putative (EC:1.1.3.15); K11517 (S)-2-hydroxy-acid oxidase [EC:1.1.3.15] | 28.169 | | | 43.009 | 51.952 | | 32.299 | | 51.595 | | 75.593 | | 57.109 | |
| Pbr027524.1 | (S)-2-hydroxy-acid oxidase, putative (EC:1.1.3.15); K11517 (S)-2-hydroxy-acid oxidase [EC:1.1.3.15] | 28.169 | | | 43.009 | 51.952 | | 32.299 | | 51.595 | | 75.593 | | 57.109 | |
| Pbr027531.1 | (S)-2-hydroxy-acid oxidase, putative (EC:1.1.3.15); K11517 (S)-2-hydroxy-acid oxidase [EC:1.1.3.15] | 28.169 | | | 43.009 | 51.952 | | 32.299 | | 51.595 | | 75.593 | | 57.109 | |
| Pbr027633.1 | hypothetical protein LOC100263580; K00844 hexokinase [EC:2.7.1.1] | 5.807 | | | 3.473 | 4.083 | | 4.146 | | 3.164 | | 1.54 | | 1.773 | |
| Pbr027666.1 | hydrolase, acting on ester bonds, putative | 3.459 | | | 7.88 | 12.39 | | 3.471 | | 6.742 | | 9.123 | | 10.89 | |
| Pbr027667.1 | hypothetical protein; K01114 phospholipase C [EC:3.1.4.3] | 9.513 | | | 20.169 | 30.129 | | 7.424 | | 17.611 | | 24.882 | | 29.504 | |
| Pbr027745.1 | similar to aldehyde dehydrogenase family 7 member A1; K14085 aldehyde dehydrogenase family 7 member A1 [EC:1.2.1.31 1.2.1.8 1.2.1.3] | 247.47 | | | 164.955 | 191.055 | | 379.391 | | 92.734 | | 86.849 | | 57.109 | |
| Pbr027868.1 | hypothetical protein; K00696 sucrose-phosphate synthase [EC:2.4.1.14] | 21.374 | | | 21.371 | 20.274 | | 17.644 | | 11.97 | | 6.28 | | 7.091 | |
| Pbr027923.1 | UDP-n-acteylglucosamine pyrophosphorylase, putative; K12447 UDP-sugar pyrophosphorylase [EC:2.7.7.64] | 25.698 | | | 26.446 | 20.133 | | 20.922 | | 18.161 | | 20.853 | | 12.283 | |
| Pbr027959.1 | hypothetical protein; K00128 aldehyde dehydrogenase (NAD+) [EC:1.2.1.3] | 28.293 | | | 44.478 | 29.144 | | 26.514 | | 39.9 | | 20.142 | | 25.579 | |
| Pbr027964.1 | hypothetical protein LOC100262362; K12449 UDP-apiose/xylose synthase | 187.054 | | | 199.95 | 115.027 | | 204.592 | | 288.932 | | 230.216 | | 125.362 | |
| Pbr027986.1 | hypothetical protein LOC100255282; K01188 beta-glucosidase [EC:3.2.1.21] | 6.301 | | | 9.35 | 5.209 | | 5.881 | | 10.181 | | 5.095 | | 2.912 | |
| Pbr027987.1 | hypothetical protein LOC100255282; K01188 beta-glucosidase [EC:3.2.1.21] | 11.614 | | | 18.833 | 7.321 | | 13.209 | | 18.712 | | 6.872 | | 5.572 | |
| Pbr028010.2 | similar to enolase, putative; K01689 enolase [EC:4.2.1.11] | 3.089 | | | 7.48 | 2.534 | | 5.014 | | 5.916 | | 5.45 | | 3.292 | |
| Pbr028261.1 | hydroxymethylglutaryl-CoA lyase (EC:4.1.3.4); K01640 hydroxymethylglutaryl-CoA lyase [EC:4.1.3.4] | 28.169 | | | 18.165 | 53.36 | | 36.734 | | 16.786 | | 45.854 | | 38.495 | |
| Pbr028275.1 | alcohol dehydrogenase (EC:1.1.1.1); K00001 alcohol dehydrogenase [EC:1.1.1.1] | 20.756 | | | 29.785 | 53.36 | | 27.96 | | 39.075 | | 56.043 | | 44.573 | |
| Pbr028302.1 | sucrose synthase, putative (EC:2.4.1.13); K00695 sucrose synthase [EC:2.4.1.13] | 1.853 | | | 1.736 | 6.476 | | 8.774 | | 8.393 | | 2.607 | | 1.266 | |
| Pbr028371.2 | aldose 1-epimerase (EC:5.1.3.3); K01785 aldose 1-epimerase [EC:5.1.3.3] | 24.586 | | | 48.752 | 46.461 | | 51.196 | | 70.857 | | 60.19 | | 54.957 | |
| Pbr028513.1 | hypothetical protein LOC100245003 | 10.131 | | | 13.09 | 7.321 | | 9.159 | | 11.695 | | 8.057 | | 3.419 | |
| Pbr028605.1 | phosphofructokinase, putative (EC:2.7.1.90); K00850 6-phosphofructokinase [EC:2.7.1.11] | 33.358 | | | 44.077 | 79.829 | | 32.974 | | 31.507 | | 60.901 | | 77.37 | |
| Pbr028606.1 | phosphofructokinase, putative (EC:2.7.1.90); K00850 6-phosphofructokinase [EC:2.7.1.11] | 33.358 | | | 44.077 | 79.829 | | 32.974 | | 31.507 | | 60.901 | | 77.37 | |
| Pbr028609.1 | hypothetical protein LOC100243946; K00850 6-phosphofructokinase [EC:2.7.1.11] | 18.903 | | | 32.056 | 45.053 | | 26.707 | | 32.47 | | 55.451 | | 49.385 | |
| Pbr028610.1 | phosphofructokinase, putative (EC:2.7.1.90); K00850 6-phosphofructokinase [EC:2.7.1.11] | 16.926 | | | 24.042 | 37.169 | | 16.198 | | 19.4 | | 39.692 | | 33.683 | |
| Pbr028619.1 | myo-inositol oxidase; K00469 inositol oxygenase [EC:1.13.99.1] | 6.548 | | | 10.285 | 6.617 | | 8.099 | | 10.044 | | 3.673 | | 4.305 | |
| Pbr028738.1 | ribose-5-phosphate isomerase (EC:5.3.1.6); K01807 ribose 5-phosphate isomerase A [EC:5.3.1.6] | 195.949 | | | 58.102 | 67.862 | | 95.932 | | 61.914 | | 24.408 | | 25.706 | |
| Pbr028810.1 | mannose-6-phosphate isomerase, putative (EC:5.3.1.8); K01809 mannose-6-phosphate isomerase [EC:5.3.1.8] | 19.397 | | | 18.432 | 5.491 | | 11.377 | | 16.51 | | 7.702 | | 3.799 | |
| Pbr028837.1 | hypothetical protein; K01187 alpha-glucosidase [EC:3.2.1.20] | 62.269 | | | 121.813 | 24.075 | | 71.443 | | 226.33 | | 86.494 | | 24.186 | |
| Pbr029047.1 | hypothetical protein LOC100254952; K00921 1-phosphatidylinositol-3-phosphate 5-kinase [EC:2.7.1.150] | 18.038 | | | 12.422 | 11.123 | | 14.655 | | 10.319 | | 3.91 | | 5.065 | |
| Pbr029060.1 | ATP-citrate synthase, putative (EC:6.2.1.5); K01648 ATP citrate (pro-S)-lyase [EC:2.3.3.8] | 127.874 | | | 86.017 | 72.367 | | 72.889 | | 178.725 | | 84.954 | | 98.137 | |
| Pbr029104.2 | hypothetical protein LOC100263079; K00975 glucose-1-phosphate adenylyltransferase [EC:2.7.7.27] | 4.695 | | | 12.689 | 1.69 | | 6.845 | | 8.943 | | 2.844 | | 1.773 | |
| Pbr029200.1 | rbcL; ribulose-1,5-bisphosphate carboxylase/oxygenase large subunit (EC:4.1.1.39); K01601 ribulose-bisphosphate carboxylase large chain [EC:4.1.1.39] | 4.448 | | | 4.007 | 6.054 | | 27.575 | | 4.953 | | 46.091 | | 21.274 | |
| Pbr029218.1 | rbcL; ribulose-1,5-bisphosphate carboxylase/oxygenase large subunit (EC:4.1.1.39) | 4.448 | | | 4.007 | 6.054 | | 27.575 | | 4.953 | | 46.091 | | 21.274 | |
| Pbr029288.1 | endo-1,4-beta-glucanase, putative (EC:3.2.1.4); K01179 endoglucanase [EC:3.2.1.4] | 60.045 | | | 5.209 | 5.632 | | 17.451 | | 24.903 | | 4.976 | | 2.659 | |
| Pbr029633.1 | hypothetical protein LOC100266026; K05605 3-hydroxyisobutyryl-CoA hydrolase [EC:3.1.2.4] | 28.169 | | | 48.084 | 45.335 | | 30.274 | | 53.796 | | 65.996 | | 47.612 | |
| Pbr029682.1 | hypothetical protein LOC100260586; K12450 UDP-glucose 4,6-dehydratase [EC:4.2.1.76] | 3.583 | | | 6.678 | 6.195 | | 5.592 | | 9.769 | | 9.716 | | 7.598 | |
| Pbr029685.2 | Glycosyltransferase QUASIMODO1, putative; K13648 alpha-1,4-galacturonosyltransferase [EC:2.4.1.43] | 13.343 | | | 8.949 | 10.841 | | 16.68 | | 8.255 | | 5.45 | | 2.912 | |
| Pbr029953.1 | hypothetical protein LOC100261061; K00012 UDPglucose 6-dehydrogenase [EC:1.1.1.22] | 471.835 | | | 604.257 | 605.828 | | 335.041 | | 610.61 | | 1161.031 | | 679.108 | |
| Pbr029954.1 | BG1; beta-glucosidase; K01188 beta-glucosidase [EC:3.2.1.21] | 0.247 | | | 0.534 | 1.267 | | 0.868 | | 0.688 | | 0.118 | | 0.127 | |
| Pbr029981.1 | 6-phosphogluconolactonase, putative (EC:3.1.1.31) | 122.438 | | | 63.711 | 43.223 | | 112.323 | | 38.524 | | 23.934 | | 23.426 | |
| Pbr030107.1 | Os12g0443600; K00012 UDPglucose 6-dehydrogenase [EC:1.1.1.22] | 48.678 | | | 24.042 | 27.736 | | 37.023 | | 75.673 | | 99.764 | | 29.631 | |
| Pbr030314.1 | hypothetical protein LOC100255703; K01051 pectinesterase [EC:3.1.1.11] | 0.124 | | | 0 | 0 | | 1.061 | | 0.275 | | 0 | | 0 | |
| Pbr030449.1 | hydrolase, acting on ester bonds, putative; K01114 phospholipase C [EC:3.1.4.3] | 3.583 | | | 7.613 | 12.249 | | 3.182 | | 6.742 | | 8.886 | | 10.763 | |
| Pbr030450.1 | hypothetical protein; K01114 phospholipase C [EC:3.1.4.3] | 9.019 | | | 18.833 | 26.187 | | 7.135 | | 17.061 | | 22.986 | | 27.352 | |
| Pbr030708.2 | glycosyltransferase, CAZy family GT8 (EC:2.4.1.43); K13648 alpha-1,4-galacturonosyltransferase [EC:2.4.1.43] | 13.343 | | | 12.288 | 9.574 | | 8.484 | | 19.4 | | 6.517 | | 8.104 | |
| Pbr030762.1 | hypothetical protein; K01193 beta-fructofuranosidase [EC:3.2.1.26] | 2.347 | | | 1.202 | 1.126 | | 6.267 | | 4.128 | | 1.303 | | 1.773 | |
| Pbr030971.1 | rbcL; ribulose-1,5-bisphosphate carboxylase/oxygenase large subunit (EC:4.1.1.39) | 4.448 | | | 4.007 | 6.054 | | 27.575 | | 4.953 | | 46.091 | | 21.274 | |
| Pbr030988.1 | rbcL; ribulose-1,5-bisphosphate carboxylase/oxygenase large subunit (EC:4.1.1.39); K01601 ribulose-bisphosphate carboxylase large chain [EC:4.1.1.39] | 4.448 | | | 4.007 | 6.054 | | 27.575 | | 4.953 | | 46.091 | | 21.274 | |
| Pbr031090.1 | hypothetical protein LOC100247690; K01006 pyruvate,orthophosphate dikinase [EC:2.7.9.1] | 17.05 | | | 25.244 | 50.404 | | 19.572 | | 5.916 | | 14.692 | | 14.562 | |
| Pbr031091.1 | hypothetical protein LOC100247690; K01006 pyruvate,orthophosphate dikinase [EC:2.7.9.1] | 5.436 | | | 6.011 | 20.978 | | 9.834 | | 2.889 | | 3.081 | | 5.445 | |
| Pbr031365.1 | hypothetical protein LOC100262240; K00135 succinate-semialdehyde dehydrogenase (NADP+) [EC:1.2.1.16] | 13.343 | | | 29.919 | 17.036 | | 21.886 | | 34.534 | | 23.46 | | 22.413 | |
| Pbr031410.1 | hypothetical protein; K00913 inositol-1,3,4-trisphosphate 5/6-kinase / inositol-tetrakisphosphate 1-kinase [EC:2.7.1.159 2.7.1.134] | 29.652 | | | 32.857 | 29.285 | | 17.74 | | 19.125 | | 29.621 | | 44.7 | |
| Pbr031443.1 | glucosamine-fructose-6-phosphate aminotransferase, putative (EC:2.6.1.16); K00820 glucosamine--fructose-6-phosphate aminotransferase (isomerizing) [EC:2.6.1.16] | 26.192 | | | 25.244 | 17.177 | | 22.465 | | 35.36 | | 20.735 | | 20.007 | |
| Pbr031455.1 | hypothetical protein LOC100256854; K01962 acetyl-CoA carboxylase carboxyl transferase subunit alpha [EC:6.4.1.2] | 2.842 | | | 3.206 | 2.253 | | 4.242 | | 2.339 | | 0.711 | | 1.14 | |
| Pbr031532.1 | hypothetical protein; K00703 starch synthase [EC:2.4.1.21] | 12.478 | | | 10.952 | 16.754 | | 12.727 | | 3.164 | | 4.384 | | 3.546 | |
| Pbr031564.1 | sucrose synthase (EC:2.4.1.13); K00695 sucrose synthase [EC:2.4.1.13] | 1.483 | | | 1.87 | 5.913 | | 7.617 | | 7.017 | | 2.725 | | 0.886 | |
| Pbr031607.1 | hypothetical protein LOC100256047; K00972 UDP-N-acetylglucosamine pyrophosphorylase [EC:2.7.7.23] | 21.498 | | | 25.645 | 40.407 | | 25.55 | | 18.437 | | 50.237 | | 32.67 | |
| Pbr031797.1 | hypothetical protein LOC100262240; K00135 succinate-semialdehyde dehydrogenase (NADP+) | 12.231 | | | 26.981 | 14.642 | | 18.126 | | 31.232 | | 20.853 | | 19.881 | |
| Pbr031801.1 | malate dehydrogenase, putative (EC:1.1.1.37); K00025 malate dehydrogenase [EC:1.1.1.37] | 0.124 | | | 0.401 | 1.408 | | 1.253 | | 1.101 | | 0.118 | | 0 | |
| Pbr032052.1 | aldose 1-epimerase, putative; K01792 glucose-6-phosphate 1-epimerase [EC:5.1.3.15] | 17.297 | | | 22.706 | 16.613 | | 24.393 | | 28.618 | | 14.218 | | 12.916 | |
| Pbr032090.1 | sucrose phosphate syntase, putative (EC:2.4.1.14); K00696 sucrose-phosphate synthase [EC:2.4.1.14] | 57.203 | | | 44.878 | 62.512 | | 42.615 | | 29.581 | | 28.673 | | 51.284 | |
| Pbr032091.1 | hypothetical protein LOC100241955; K00696 sucrose-phosphate synthase [EC:2.4.1.14] | 9.637 | | | 8.415 | 9.433 | | 6.653 | | 4.265 | | 5.332 | | 7.344 | |
| Pbr032092.1 | hypothetical protein LOC100241955; K00696 sucrose-phosphate synthase [EC:2.4.1.14] | 9.637 | | | 8.415 | 9.433 | | 6.653 | | 4.265 | | 5.332 | | 7.344 | |
| Pbr032093.1 | sucrose phosphate syntase, putative (EC:2.4.1.14); K00696 sucrose-phosphate synthase [EC:2.4.1.14] | 57.203 | | | 44.878 | 62.512 | | 42.615 | | 29.581 | | 28.673 | | 51.284 | |
| Pbr032243.1 | hypothetical protein; K01728 pectate lyase [EC:4.2.2.2] | 15.444 | | | 12.422 | 8.307 | | 17.933 | | 32.608 | | 11.73 | | 14.562 | |
| Pbr032318.1 | hypothetical protein LOC100251825; K12373 hexosaminidase [EC:3.2.1.52] | 71.041 | | | 65.848 | 51.671 | | 65.658 | | 59.713 | | 40.522 | | 33.43 | |
| Pbr032451.1 | 6-phosphofructokinase (EC:2.7.1.11); K00850 6-phosphofructokinase [EC:2.7.1.11] | 11.119 | | | 11.353 | 3.942 | | 12.245 | | 10.319 | | 4.858 | | 3.672 | |
| Pbr032600.1 | mannose-6-phosphate isomerase (EC:5.3.1.8); K01809 mannose-6-phosphate isomerase [EC:5.3.1.8] | 7.784 | | | 20.97 | 4.928 | | 5.399 | | 8.393 | | 3.555 | | 4.305 | |
| Pbr032602.1 | Ribulose bisphosphate carboxylase small chain, chloroplast precursor, putative (EC:4.1.1.39); K01602 ribulose-bisphosphate carboxylase small chain [EC:4.1.1.39] | 64.863 | | | 155.739 | 87.15 | | 61.416 | | 45.266 | | 70.498 | | 72.558 | |
| Pbr032603.1 | Ribulose bisphosphate carboxylase small chain 1B, chloroplast precursor, putative (EC:4.1.1.39); K01602 ribulose-bisphosphate carboxylase small chain [EC:4.1.1.39] | 91.55 | | | 219.584 | 117.139 | | 68.165 | | 57.374 | | 96.92 | | 103.962 | |
| Pbr032770.1 | sorbitol dehydrogenase | 1.73 | | | 0.801 | 0.282 | | 1.639 | | 0.55 | | 0.118 | | 0.633 | |
| Pbr032771.1 | L-iditol 2-dehydrogenase (EC:1.1.1.14); K00008 L-iditol 2-dehydrogenase [EC:1.1.1.14] | 1.235 | | | 0.801 | 0.845 | | 2.218 | | 0 | | 0 | | 0.633 | |
| Pbr032772.1 | alcohol dehydrogenase, putative (EC:1.1.1.14); K00008 L-iditol 2-dehydrogenase [EC:1.1.1.14] | 94.886 | | | 98.84 | 174.16 | | 97.186 | | 46.642 | | 177.372 | | 445.985 | |
| Pbr032773.1 | alcohol dehydrogenase, putative (EC:1.1.1.14); K00008 L-iditol 2-dehydrogenase [EC:1.1.1.14] | 261.184 | | | 361.031 | 662.426 | | 218.186 | | 115.298 | | 741.359 | | 1875.366 | |
| Pbr032774.1 | alcohol dehydrogenase, putative (EC:1.1.1.14); K00008 L-iditol 2-dehydrogenase [EC:1.1.1.14] | 260.195 | | | 363.168 | 658.906 | | 214.522 | | 113.234 | | 728.681 | | 1846.748 | |
| Pbr032775.1 | L-iditol 2-dehydrogenase (EC:1.1.1.14); K00008 L-iditol 2-dehydrogenase [EC:1.1.1.14] | 92.786 | | | 98.038 | 146.987 | | 156.481 | | 82.139 | | 152.253 | | 214.762 | |
| Pbr032776.1 | L-iditol 2-dehydrogenase (EC:1.1.1.14); K00008 L-iditol 2-dehydrogenase [EC:1.1.1.14] | 55.597 | | | 66.249 | 114.323 | | 59.199 | | 29.306 | | 117.892 | | 287.446 | |
| Pbr032777.1 | L-iditol 2-dehydrogenase (EC:1.1.1.14); K00008 L-iditol 2-dehydrogenase [EC:1.1.1.14] | 346.556 | | | 227.464 | 113.76 | | 311.901 | | 278.751 | | 54.858 | | 120.17 | |
| Pbr032778.1 | sorbitol dehydrogenase, putative / L-iditol 2-dehydrogenase, putative (EC:1.1.1.14); K00008 L-iditol 2-dehydrogenase [EC:1.1.1.14] | 19.644 | | | 17.23 | 24.216 | | 32.492 | | 20.363 | | 30.451 | | 60.148 | |
| Pbr032986.2 | Pectate lyase precursor, putative (EC:4.2.2.2); K01728 pectate lyase [EC:4.2.2.2] | 3.336 | | | 1.069 | 3.379 | | 9.738 | | 12.658 | | 7.465 | | 2.406 | |
| Pbr033075.1 | Glycosyltransferase QUASIMODO1, putative; K13648 alpha-1,4-galacturonosyltransferase [EC:2.4.1.43] | 61.281 | | | 88.154 | 26.187 | | 44.544 | | 100.576 | | 38.508 | | 22.033 | |
| Pbr033247.1 | hypothetical protein; K08232 monodehydroascorbate reductase (NADH) [EC:1.6.5.4] | 0.494 | | | 0 | 1.126 | | 1.061 | | 1.513 | | 0.592 | | 0.633 | |
| Pbr033440.1 | hypothetical protein LOC100254952; K00921 1-phosphatidylinositol-3-phosphate 5-kinase [EC:2.7.1.150] | 6.919 | | | 3.473 | 3.661 | | 4.242 | | 2.889 | | 0.948 | | 0.38 | |
| Pbr033557.1 | hypothetical protein; K01580 glutamate decarboxylase | 177.17 | | | 283.429 | 223.859 | | 250.871 | | 410.559 | | 258.415 | | 184.244 | |
| Pbr033571.1 | hypothetical protein LOC100266759; K00695 sucrose synthase [EC:2.4.1.13] | 1.73 | | | 1.736 | 2.112 | | 1.735 | | 3.027 | | 0.711 | | 1.013 | |
| Pbr033653.1 | glycosyltransferase, CAZy family GT8 (EC:2.4.1.43); K13648 alpha-1,4-galacturonosyltransferase [EC:2.4.1.43] | 0 | | | 4.808 | 278.205 | | 51.871 | | 11.007 | | 206.4 | | 166.136 | |
| Pbr033724.1 | hypothetical protein LOC100261773; K00849 galactokinase | 20.509 | | | 30.72 | 25.765 | | 16.005 | | 24.49 | | 34.361 | | 23.933 | |
| Pbr033748.1 | hypothetical protein LOC100244966; K01103 6-phosphofructo-2-kinase / fructose-2,6-bisphosphatase [EC:2.7.1.105 3.1.3.46] | 25.204 | | | 34.193 | 33.931 | | 48.304 | | 35.36 | | 18.484 | | 24.059 | |
| Pbr033821.1 | hypothetical protein LOC100251292; K01784 UDP-glucose 4-epimerase [EC:5.1.3.2] | 72.029 | | | 31.522 | 75.183 | | 56.885 | | 17.336 | | 19.905 | | 67.999 | |
| Pbr033934.1 | ascorbate peroxidase; K00434 L-ascorbate peroxidase [EC:1.11.1.11] | 803.319 | | | 1128.908 | 949.923 | | 419.789 | | 768.285 | | 1291.246 | | 871.456 | |
| Pbr034263.1 | glyceraldehyde-3-phosphate dehydrogenase (NADP+)/3-chloroallyl aldehyde dehydrogenase (EC:1.2.1.13); K00131 glyceraldehyde-3-phosphate dehydrogenase (NADP) [EC:1.2.1.9] | 44.354 | | | 81.476 | 39.563 | | 75.3 | | 95.348 | | 65.048 | | 60.148 | |
| Pbr034361.1 | hypothetical protein LOC100241955; K00696 sucrose-phosphate synthase [EC:2.4.1.14] | 64.616 | | | 50.488 | 69.692 | | 47.436 | | 33.158 | | 32.82 | | 57.236 | |
| Pbr034362.1 | Phosphoenolpyruvate carboxylase, putative (EC:1.3.1.74); K01595 phosphoenolpyruvate carboxylase [EC:4.1.1.31] | 527.556 | | | 290.241 | 226.112 | | 262.44 | | 678.854 | | 358.89 | | 357.598 | |
| Pbr034504.1 | myo-inositol-1 phosphate synthase, putative (EC:5.5.1.4); K01858 myo-inositol-1-phosphate synthase [EC:5.5.1.4] | 47.319 | | | 83.346 | 52.797 | | 67.201 | | 124.103 | | 69.787 | | 56.603 | |
| Pbr034507.1 | myo-inositol-1 phosphate synthase, putative (EC:5.5.1.4); K01858 myo-inositol-1-phosphate synthase [EC:5.5.1.4] | 49.79 | | | 87.486 | 55.613 | | 69.708 | | 128.919 | | 71.802 | | 59.389 | |
| Pbr034527.1 | hypothetical protein LOC100243846 | 18.532 | | | 23.241 | 16.613 | | 27.382 | | 37.974 | | 16.943 | | 19.627 | |
| Pbr034729.1 | malic enzyme, putative (EC:1.1.1.40); K00029 malate dehydrogenase (oxaloacetate-decarboxylating)(NADP+) [EC:1.1.1.40] | 153.943 | | | 181.117 | 154.73 | | 179.524 | | 169.92 | | 83.176 | | 102.696 | |
| Pbr034732.1 | ATP-citrate synthase, putative (EC:2.3.3.8); K01648 ATP citrate (pro-S)-lyase [EC:2.3.3.8] | 6.054 | | | 7.346 | 3.942 | | 5.592 | | 13.759 | | 6.161 | | 6.458 | |
| Pbr034762.1 | Ribulose bisphosphate carboxylase small chain 1B, chloroplast precursor, putative (EC:4.1.1.39); K01602 ribulose-bisphosphate carboxylase small chain [EC:4.1.1.39] | 735.366 | | | 1671.857 | 968.648 | | 639.229 | | 564.932 | | 895.152 | | 918.435 | |
| Pbr034766.1 | mannose-6-phosphate isomerase (EC:5.3.1.8) | 4.448 | | | 10.418 | 2.675 | | 3.375 | | 4.128 | | 4.265 | | 3.672 | |
| Pbr034871.1 | aldehyde dehydrogenase (NAD+) (EC:1.2.1.3); K00128 aldehyde dehydrogenase (NAD+) [EC:1.2.1.3] | 45.96 | | | 77.736 | 94.894 | | 92.654 | | 112.133 | | 95.262 | | 113.712 | |
| Pbr034873.1 | aldehyde dehydrogenase (NAD+) [EC:1.2.1.3] | 34.964 | | | 51.423 | 64.342 | | 68.744 | | 84.203 | | 65.641 | | 75.344 | |
| Pbr034954.1 | hypothetical protein LOC100253688; K00975 glucose-1-phosphate adenylyltransferase [EC:2.7.7.27] | 17.173 | | | 30.186 | 30.833 | | 28.346 | | 40.175 | | 52.252 | | 33.683 | |
| Pbr035064.1 | hypothetical protein LOC100251292; K01784 UDP-glucose 4-epimerase [EC:5.1.3.2] | 374.478 | | | 121.546 | 393.654 | | 304.188 | | 61.914 | | 87.797 | | 372.034 | |
| Pbr035106.1 | hypothetical protein LOC100246241; K01194 alpha,alpha-trehalase [EC:3.2.1.28] | 19.027 | | | 23.775 | 10.7 | | 9.834 | | 42.79 | | 13.744 | | 17.601 | |
| Pbr035229.1 | hypothetical protein LOC100262177; K01728 pectate lyase [EC:4.2.2.2] | 3.583 | | | 1.336 | 0.563 | | 0.096 | | 0.138 | | 0 | | 0 | |
| Pbr035260.1 | myo-inositol oxidase; K00469 inositol oxygenase [EC:1.13.99.1] | 8.031 | | | 10.952 | 7.04 | | 8.484 | | 10.869 | | 4.384 | | 5.825 | |
| Pbr035287.1 | pyruvate kinase (EC:2.7.1.40); K00873 pyruvate kinase [EC:2.7.1.40] | 1.73 | | | 3.473 | 1.69 | | 2.218 | | 4.678 | | 3.199 | | 2.659 | |
| Pbr035305.1 | Pectate lyase precursor, putative (EC:4.2.2.2) | 3.336 | | | 5.743 | 2.112 | | 5.014 | | 20.776 | | 1.54 | | 1.52 | |
| Pbr035476.2 | disproportionating enzyme (EC:2.4.1.25); K00705 4-alpha-glucanotransferase [EC:2.4.1.25] | 6.548 | | | 14.559 | 8.729 | | 8.099 | | 12.52 | | 9.716 | | 8.737 | |
| Pbr035571.1 | 2-isopropylmalate synthase (EC:2.3.3.13); K01649 2-isopropylmalate synthase [EC:2.3.3.13] | 2.224 | | | 9.083 | 0.986 | | 3.375 | | 9.769 | | 0.711 | | 0.76 | |
| Pbr035637.1 | hypothetical protein LOC100267681; K00036 glucose-6-phosphate 1-dehydrogenase [EC:1.1.1.49] | 30.887 | | | 21.771 | 5.632 | | 7.135 | | 9.356 | | 3.91 | | 7.091 | |
| Pbr035655.1 | neutral alpha-glucosidase ab precursor, putative (EC:3.2.1.20); K01187 alpha-glucosidase [EC:3.2.1.20] | 18.903 | | | 4.942 | 18.303 | | 16.583 | | 12.383 | | 2.37 | | 1.266 | |
| Pbr035932.1 | fructose-1,6-bisphosphatase, putative (EC:1.3.1.74); K03841 fructose-1,6-bisphosphatase I [EC:3.1.3.11] | 62.64 | | | 163.219 | 66.735 | | 59.199 | | 112.133 | | 106.281 | | 52.424 | |
| Pbr035949.1 | acetyl-CoA carboxylase (EC:6.4.1.2); K01962 acetyl-CoA carboxylase carboxyl transferase subunit alpha [EC:6.4.1.2] | 26.687 | | | 34.06 | 16.473 | | 28.635 | | 36.323 | | 25.474 | | 20.134 | |
| Pbr035996.1 | PtrSuSY2; sucrose synthase (EC:2.4.1.13); K00695 sucrose synthase [EC:2.4.1.13] | 6.301 | | | 8.548 | 63.356 | | 21.404 | | 16.235 | | 124.883 | | 55.463 | |
| Pbr035997.1 | PtrSuSY2; sucrose synthase (EC:2.4.1.13); K00695 sucrose synthase [EC:2.4.1.13] | 6.672 | | | 9.884 | 49.418 | | 16.294 | | 17.749 | | 93.484 | | 44.7 | |
| Pbr036026.1 | hypothetical protein LOC100255282; K01188 beta-glucosidase [EC:3.2.1.21] | 0.124 | | | 0.534 | 1.408 | | 0.578 | | 0.55 | | 0.474 | | 1.14 | |
| Pbr036040.1 | K01188 beta-glucosidase [EC:3.2.1.21] | 0.124 | | | 0.534 | 1.408 | | 0.578 | | 0.55 | | 0.474 | | 1.14 | |
| Pbr036221.1 | Pectate lyase precursor, putative (EC:4.2.2.2); K01728 pectate lyase [EC:4.2.2.2] | 13.714 | | | 30.72 | 2.534 | | 10.22 | | 20.225 | | 3.792 | | 1.646 | |
| Pbr036303.1 | phosphoenolpyruvate carboxykinase (ATP) (EC:4.1.1.49); K01610 phosphoenolpyruvate carboxykinase (ATP) [EC:4.1.1.49] | 19.027 | | | 52.492 | 40.689 | | 15.426 | | 28.756 | | 41.944 | | 35.076 | |
| Pbr036345.1 | fructose-bisphosphatase (EC:3.1.3.11) | 37.188 | | | 45.012 | 51.53 | | 38.566 | | 18.161 | | 34.953 | | 39.128 | |
| Pbr036382.1 | Polygalacturonase precursor, putative (EC:3.2.1.67); K01184 polygalacturonase [EC:3.2.1.15] | 17.668 | | | 1.603 | 0.282 | | 25.839 | | 2.064 | | 0.474 | | 0.127 | |
| Pbr036396.1 | glucose-1-phosphate adenylyltransferase, putative (EC:2.7.7.27); K00975 glucose-1-phosphate adenylyltransferase [EC:2.7.7.27] | 49.543 | | | 42.474 | 23.371 | | 43.097 | | 54.347 | | 24.171 | | 26.339 | |
| Pbr036641.1 | hypothetical protein LOC100263580; K00844 hexokinase [EC:2.7.1.1] | 4.818 | | | 3.339 | 4.083 | | 3.953 | | 2.752 | | 1.54 | | 1.52 | |
| Pbr036711.1 | pyruvate kinase (EC:2.7.1.40); K00873 pyruvate kinase [EC:2.7.1.40] | 1.73 | | | 3.473 | 1.69 | | 2.218 | | 4.678 | | 3.199 | | 2.659 | |
| Pbr036852.1 | hypothetical protein; K01193 beta-fructofuranosidase [EC:3.2.1.26] | 12.108 | | | 5.209 | 10.137 | | 37.312 | | 22.151 | | 3.673 | | 11.65 | |
| Pbr036856.1 | hypothetical protein; K01193 beta-fructofuranosidase [EC:3.2.1.26] | 12.108 | | | 5.209 | 10.137 | | 37.312 | | 22.151 | | 3.673 | | 11.65 | |
| Pbr036881.1 | Phospholipase C 4 precursor, putative (EC:3.1.4.3); K01114 phospholipase C [EC:3.1.4.3] | 0.618 | | | 0.534 | 0.845 | | 1.543 | | 3.027 | | 1.066 | | 0.507 | |
| Pbr036882.1 | Phospholipase C 4 precursor, putative (EC:3.1.4.3); K01114 phospholipase C [EC:3.1.4.3] | 3.83 | | | 4.007 | 1.971 | | 6.363 | | 11.282 | | 3.436 | | 2.406 | |
| Pbr036977.1 | hypothetical protein; K01728 pectate lyase [EC:4.2.2.2] | 49.296 | | | 34.327 | 37.31 | | 129.581 | | 73.334 | | 111.968 | | 33.936 | |
| Pbr037078.1 | ATP-citrate synthase, putative (EC:2.3.3.8); K01648 ATP citrate (pro-S)-lyase [EC:2.3.3.8] | 13.467 | | | 19.367 | 10.559 | | 12.92 | | 30.407 | | 13.27 | | 11.27 | |
| Pbr037161.1 | acetolactate synthase, putative (EC:2.2.1.6); K01652 acetolactate synthase I/II/III large subunit [EC:2.2.1.6] | 3.336 | | | 5.476 | 3.238 | | 2.025 | | 6.191 | | 4.265 | | 6.838 | |
| Pbr037260.1 | hypothetical protein; K11517 (S)-2-hydroxy-acid oxidase [EC:1.1.3.15] | 152.954 | | | 308.406 | 236.671 | | 143.465 | | 151.758 | | 199.765 | | 192.222 | |
| Pbr037378.1 | pectinesterase family protein (EC:3.1.1.11); K01051 pectinesterase [EC:3.1.1.11] | 9.143 | | | 32.724 | 2.253 | | 6.749 | | 43.34 | | 9.36 | | 1.899 | |
| Pbr037383.1 | hypothetical protein LOC100267980; K00921 1-phosphatidylinositol-3-phosphate 5-kinase [EC:2.7.1.150] | 80.554 | | | 57.033 | 57.021 | | 71.54 | | 56.961 | | 22.157 | | 29.884 | |
| Pbr037395.1 | PtrSuSY2; sucrose synthase (EC:2.4.1.13); K00695 sucrose synthase [EC:2.4.1.13] | 60.416 | | | 80.808 | 659.047 | | 222.332 | | 143.228 | | 1338.047 | | 643.019 | |
| Pbr037426.1 | similar to aldehyde dehydrogenase family 7 member A1; K14085 aldehyde dehydrogenase family 7 member A1 [EC:1.2.1.31 1.2.1.8 1.2.1.3] | 23.598 | | | 15.494 | 19.429 | | 37.312 | | 5.916 | | 8.531 | | 5.698 | |
| Pbr037938.1 | isocitrate dehydrogenase, putative (EC:1.1.1.41); K00030 isocitrate dehydrogenase (NAD+) [EC:1.1.1.41] | 2.718 | | | 1.736 | 2.534 | | 2.41 | | 4.128 | | 1.896 | | 1.266 | |
| Pbr037971.1 | pyruvate decarboxylase, putative (EC:4.1.1.1); K01568 pyruvate | 13.961 | | | 58.502 | 38.014 | | 15.041 | | 24.078 | | 68.366 | | 20.767 | |
| Pbr038213.1 | Phosphoenolpyruvate carboxylase, putative (EC:1.3.1.74); K01595 phosphoenolpyruvate carboxylase [EC:4.1.1.31] | 268.597 | | | 184.189 | 106.157 | | 153.492 | | 383.455 | | 194.433 | | 179.052 | |
| Pbr038232.1 | malate dehydrogenase, putative (EC:1.1.1.37); K00026 malate dehydrogenase [EC:1.1.1.37] | 119.472 | | | 121.546 | 75.746 | | 102.007 | | 164.829 | | 118.366 | | 93.705 | |
| Pbr038292.1 | 3-hydroxyacyl-CoA dehyrogenase, putative (EC:1.1.1.157); K00074 3-hydroxybutyryl-CoA dehydrogenase [EC:1.1.1.157] | 15.814 | | | 11.887 | 11.545 | | 14.077 | | 8.118 | | 6.161 | | 7.851 | |
| Pbr038344.1 | hypothetical protein; K02160 acetyl-CoA carboxylase biotin carboxyl carrier protein | 12.973 | | | 16.295 | 5.772 | | 11.377 | | 20.088 | | 10.782 | | 10.13 | |
| Pbr038359.1 | hypothetical protein LOC100242366; K13648 alpha-1,4-galacturonosyltransferase [EC:2.4.1.43] | 25.204 | | | 10.285 | 7.462 | | 10.027 | | 10.044 | | 3.436 | | 3.419 | |
| Pbr038453.1 | similar to starch synthase; K00703 starch synthase [EC:2.4.1.21] | 50.038 | | | 73.195 | 47.728 | | 62.669 | | 92.871 | | 52.015 | | 33.303 | |
| Pbr038586.1 | hypothetical protein LOC100256495; K00001 alcohol dehydrogenase [EC:1.1.1.1] | 9.76 | | | 5.476 | 0.282 | | 2.41 | | 2.614 | | 0.355 | | 0.38 | |
| Pbr038846.1 | hypothetical protein LOC100250907; aldose 1-epimerase | 6.054 | | | 10.685 | 8.307 | | 10.991 | | 16.786 | | 12.678 | | 12.41 | |
| Pbr038975.1 | fructose-bisphosphate aldolase, putative (EC:4.1.2.13); K01623 fructose-bisphosphate aldolase, class I [EC:4.1.2.13] | 232.273 | | | 450.788 | 279.19 | | 180.97 | | 205.417 | | 240.168 | | 296.184 | |
| Pbr039133.1 | Pectate lyase precursor, putative (EC:4.2.2.2); K01728 pectate lyase [EC:4.2.2.2] | 10.378 | | | 4.007 | 1.83 | | 0.289 | | 0.275 | | 0.237 | | 0.38 | |
| Pbr039283.1 | UDP-glucose 4-epimerase (EC:5.1.3.2); K01784 UDP-glucose 4-epimerase [EC:5.1.3.2] | 254.018 | | | 181.25 | 217.946 | | 549.274 | | 33.984 | | 23.934 | | 80.536 | |
| Pbr039291.1 | phosphoglycerate mutase, putative (EC:5.4.2.1); K01834 phosphoglycerate mutase [EC:5.4.2.1] | 4.077 | | | 10.018 | 6.899 | | 4.146 | | 6.467 | | 8.057 | | 6.078 | |
| Pbr039377.1 | acetolactate synthase, putative (EC:2.2.1.6); K01652 acetolactate synthase I/II/III large subunit [EC:2.2.1.6] | 88.214 | | | 92.963 | 94.894 | | 68.647 | | 145.429 | | 106.162 | | 139.418 | |
| Pbr039378.1 | alcohol dehydrogenase, putative (EC:1.1.1.284); K00001 alcohol dehydrogenase [EC:1.1.1.1] | 15.444 | | | 44.211 | 60.4 | | 18.126 | | 18.849 | | 65.641 | | 45.586 | |
| Pbr039379.1 | alcohol dehydrogenase, putative (EC:1.1.1.284); K00001 alcohol dehydrogenase [EC:1.1.1.1] | 39.536 | | | 103.915 | 164.727 | | 39.048 | | 45.128 | | 182.822 | | 123.969 | |
| Pbr039380.2 | hypothetical protein; K00001 alcohol dehydrogenase [EC:1.1.1.1] | 38.547 | | | 82.678 | 46.039 | | 13.884 | | 21.739 | | 56.28 | | 28.111 | |
| Pbr039481.1 | hypothetical protein LOC100251280; K00423 L-ascorbate oxidase [EC:1.10.3.3] | 22.61 | | | 72.794 | 45.335 | | 94.968 | | 261.828 | | 77.726 | | 34.316 | |
| Pbr039508.1 | trehalose-6-phosphate synthase, putative (EC:2.4.1.15 3.1.3.12); K00697 alpha,alpha-trehalose-phosphate synthase (UDP-forming) [EC:2.4.1.15] | 0.865 | | | 3.339 | 1.267 | | 1.061 | | 0.963 | | 0.829 | | 0.253 | |
| Pbr039602.1 | hypothetical protein; K11517 (S)-2-hydroxy-acid oxidase [EC:1.1.3.15] | 108.847 | | | 99.908 | 73.493 | | 87.255 | | 56.135 | | 42.773 | | 110.673 | |
| Pbr039643.1 | mannose-6-phosphate isomerase (EC:5.3.1.8); K01809 mannose-6-phosphate isomerase [EC:5.3.1.8] | 5.189 | | | 12.956 | 3.379 | | 3.953 | | 5.091 | | 5.332 | | 4.685 | |
| Pbr039673.1 | hypothetical protein LOC100253688 | 18.532 | | | 34.193 | 35.057 | | 30.081 | | 44.991 | | 59.124 | | 37.609 | |
| Pbr039758.1 | hypothetical protein; K14190 GDP-L-galactose phosphorylase [EC:2.7.7.69] | 95.133 | | | 116.871 | 91.092 | | 96.704 | | 85.304 | | 56.636 | | 56.983 | |
| Pbr039964.1 | hypothetical protein LOC100256264; K00913 inositol-1,3,4-trisphosphate 5/6-kinase / inositol-tetrakisphosphate 1-kinase [EC:2.7.1.159 2.7.1.134] | 55.721 | | | 57.968 | 117.984 | | 52.257 | | 72.371 | | 131.163 | | 104.595 | |
| Pbr039996.1 | hypothetical protein LOC100264923; K00851 gluconokinase [EC:2.7.1.12] | 14.826 | | | 8.949 | 5.632 | | 11.377 | | 4.678 | | 5.213 | | 6.331 | |
| Pbr040043.1 | ATP-citrate synthase, putative (EC:2.3.3.8); K01648 ATP citrate (pro-S)-lyase [EC:2.3.3.8] | 32.123 | | | 35.262 | 15.206 | | 29.31 | | 63.015 | | 29.147 | | 27.478 | |
| Pbr040150.1 | hypothetical protein LOC100245266; K00627 pyruvate dehydrogenase E2 component (dihydrolipoamide acetyltransferase) [EC:2.3.1.12] | 22.61 | | | 28.85 | 11.686 | | 21.308 | | 42.239 | | 16.706 | | 17.475 | |
| Pbr040265.3 | acetyl-CoA C-acetyltransferase (EC:2.3.1.9); K00626 acetyl-CoA C-acetyltransferase [EC:2.3.1.9] | 818.268 | | | 526.788 | 221.466 | | 644.242 | | 936.141 | | 255.927 | | 319.61 | |
| Pbr040288.1 | glycosyltransferase, family GT8 (EC:2.4.1.43); K13648 alpha-1,4-galacturonosyltransferase [EC:2.4.1.43] | 1.483 | | | 4.274 | 1.126 | | 2.218 | | 3.99 | | 1.896 | | 1.899 | |
| Pbr040751.1 | hypothetical protein; K01114 phospholipase C [EC:3.1.4.3] | 5.683 | | | 0.801 | 5.772 | | 2.796 | | 2.064 | | 0.948 | | 1.52 | |
| Pbr040752.1 | hypothetical protein; K01114 phospholipase C [EC:3.1.4.3] | 5.066 | | | 8.281 | 16.613 | | 11.281 | | 19.813 | | 12.204 | | 31.91 | |
| Pbr040797.1 | hypothetical protein LOC100247244; K00873 pyruvate kinase [EC:2.7.1.40] | 45.837 | | | 38.868 | 43.786 | | 24.779 | | 21.876 | | 29.858 | | 28.998 | |
| Pbr040861.1 | hypothetical protein; K00913 inositol-1,3,4-trisphosphate 5/6-kinase / inositol-tetrakisphosphate 1-kinase [EC:2.7.1.159 2.7.1.134] | 29.775 | | | 32.19 | 28.862 | | 18.222 | | 18.849 | | 29.858 | | 44.32 | |
| Pbr040907.1 | aldo-keto reductase, putative (EC:1.1.1.107); K00064 D-threo-aldose 1-dehydrogenase [EC:1.1.1.122] | 21.498 | | | 50.221 | 23.231 | | 33.938 | | 50.357 | | 31.043 | | 28.618 | |
| Pbr041170.1 | hypothetical protein LOC100260576 | 44.354 | | | 91.36 | 57.162 | | 51.196 | | 88.193 | | 92.655 | | 66.607 | |
| Pbr041218.1 | hypothetical protein LOC100261773; K00849 galactokinase [EC:2.7.1.6] | 4.201 | | | 8.682 | 6.899 | | 4.242 | | 7.43 | | 12.678 | | 6.965 | |
| Pbr041242.1 | rbcL; ribulose-1,5-bisphosphate carboxylase/oxygenase large subunit (EC:4.1.1.39); K01601 ribulose-bisphosphate carboxylase large chain [EC:4.1.1.39] | 4.448 | | | 4.007 | 6.054 | | 27.575 | | 4.953 | | 46.091 | | 21.274 | |
| Pbr041255.1 | hypothetical protein; K01961 acetyl-CoA carboxylase, biotin carboxylase subunit [EC:6.4.1.2 6.3.4.14] | 34.47 | | | 41.139 | 20.978 | | 32.974 | | 55.172 | | 29.029 | | 25.199 | |
| Pbr041275.2 | hypothetical protein; K01728 pectate lyase [EC:4.2.2.2] | 85.743 | | | 65.715 | 50.685 | | 97.282 | | 120.801 | | 33.294 | | 26.339 | |
| Pbr041369.1 | hypothetical protein LOC100255282; K01188 beta-glucosidase | 18.038 | | | 29.118 | 12.53 | | 22.754 | | 29.031 | | 12.915 | | 11.903 | |
| Pbr041370.1 | hypothetical protein LOC100255282; K01188 beta-glucosidase [EC:3.2.1.21] | 11.984 | | | 20.302 | 8.025 | | 14.269 | | 19.262 | | 7.346 | | 6.711 | |
| Pbr041854.1 | hypothetical protein LOC100256158; K01785 aldose 1-epimerase [EC:5.1.3.3] | 37.683 | | | 16.295 | 5.772 | | 46.086 | | 8.393 | | 2.607 | | 6.078 | |
| Pbr042197.1 | hypothetical protein; K05857 phospholipase C, delta [EC:3.1.4.11] | 5.066 | | | 6.144 | 7.884 | | 3.567 | | 5.916 | | 3.199 | | 2.786 | |
| Pbr042364.1 | rbcL; ribulose-1,5-bisphosphate carboxylase/oxygenase large subunit (EC:4.1.1.39); K01601 ribulose-bisphosphate | 4.448 | | | 4.007 | 6.054 | | 27.575 | | 4.953 | | 46.091 | | 21.274 | |
| Pbr042382.1 | rbcL; ribulose-1,5-bisphosphate carboxylase/oxygenase large subunit (EC:4.1.1.39); K01601 ribulose-bisphosphate carboxylase large chain [EC:4.1.1.39] | 4.448 | | | 4.007 | 6.054 | | 27.575 | | 4.953 | | 46.091 | | 21.274 | |
| Pbr042465.1 | hypothetical protein; K01728 pectate lyase [EC:4.2.2.2] | 5.56 | | | 8.949 | 6.758 | | 8.484 | | 17.474 | | 9.716 | | 8.737 | |
| Pbr042506.1 | hypothetical protein; K00696 sucrose-phosphate synthase [EC:2.4.1.14] | 5.066 | | | 5.877 | 4.928 | | 4.049 | | 4.54 | | 2.37 | | 2.659 | |
| Pbr042536.1 | glucose-1-phosphate adenylyltransferase (EC:2.7.7.27) | 3.212 | | | 5.343 | 5.069 | | 4.242 | | 7.98 | | 5.806 | | 5.572 | |
| Pbr042601.1 | 2-oxoglutarate dehydrogenase, putative (EC:1.2.4.2); K00164 2-oxoglutarate dehydrogenase E1 component [EC:1.2.4.2] | 27.675 | | | 25.244 | 12.953 | | 19.669 | | 18.849 | | 15.048 | | 12.283 | |
| Pbr042708.1 | hypothetical protein; K00851 gluconokinase [EC:2.7.1.12] | 2.471 | | | 0.134 | 0.563 | | 1.446 | | 0.963 | | 0.237 | | 0.507 | |
| Pbr042753.1 | hypothetical protein LOC100242408; K06118 UDP-sulfoquinovose synthase [EC:3.13.1.1] | 10.625 | | | 33.792 | 13.516 | | 18.897 | | 24.49 | | 18.484 | | 15.575 | |
| Pbr042868.1 | PDC1; pyruvate decarboxylase 1; K01568 pyruvate decarboxylase [EC:4.1.1.1] | 9.637 | | | 30.32 | 20.133 | | 8.581 | | 12.933 | | 37.678 | | 11.143 | |
| Pbr042913.1 | hypothetical protein; K00434 L-ascorbate peroxidase [EC:1.11.1.11] | 11.243 | | | 20.703 | 5.069 | | 8.388 | | 18.987 | | 5.569 | | 4.559 | |
