# Supplementary material for: Identifying the candidate genes involved in the calyx abscission process of 'Kuerlexiangli’ (Pyrus sinkiangensis Yu) by digital transcript abundance measurements
Source: BMC Genomics. 2013 Oct 23;14(1):727. doi: 10.1186/1471-2164-14-727 (PMC4046677; doi:10.1186/1471-2164-14-727)
Supplement: Supplementary file 5 — Additional file 5: Real-time quantitative PCR analysis of selected differential genes detected via digital transcript abundance measurements. Representative genes selected for the analysis were those involved in photosynthesis, plant hormone signal transduction, carbohydrate metabolism, cell wall degradation, and other processes. (DOC 88 KB) [file 12864_2013_5444_MOESM5_ESM.doc]

**Additional file 5: Real-time quantitative PCR analysis of selected differential genes detected via digital transcript abundance measurements.**

**
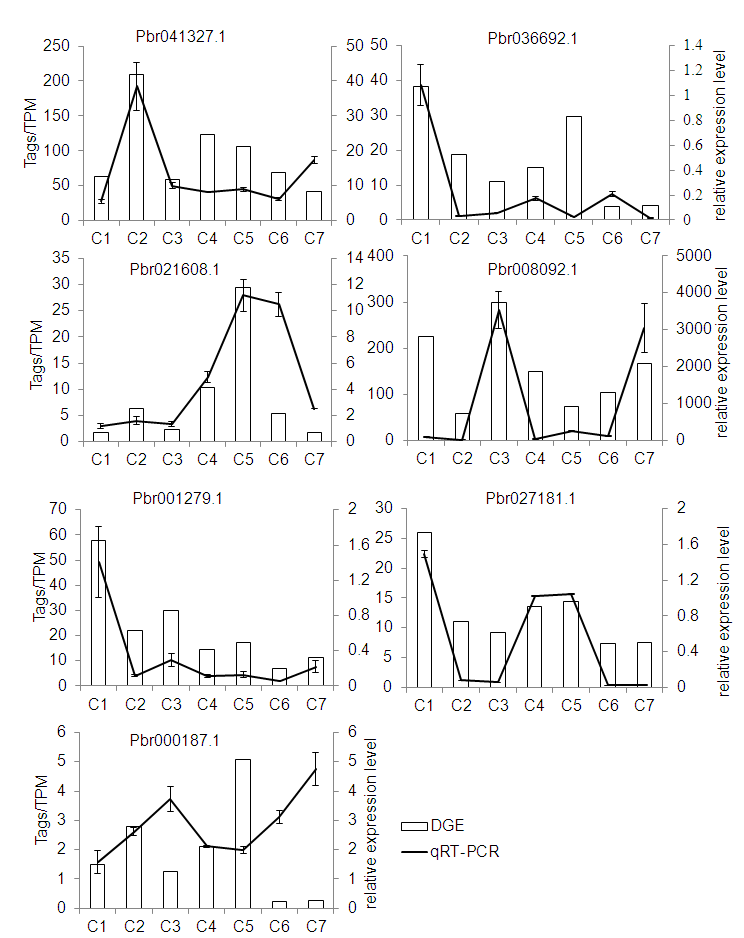
**
