# Supplementary figures and images for: Identifying the candidate genes involved in the calyx abscission process of 'Kuerlexiangli’ (Pyrus sinkiangensis Yu) by digital transcript abundance measurements
Source: BMC Genomics. 2013 Oct 23;14(1):727. doi: 10.1186/1471-2164-14-727 (PMC4046677; doi:10.1186/1471-2164-14-727)

**Additional file 6: A pear fruit with calyx tube (white arrow) at 22 d after full bloom.**

**
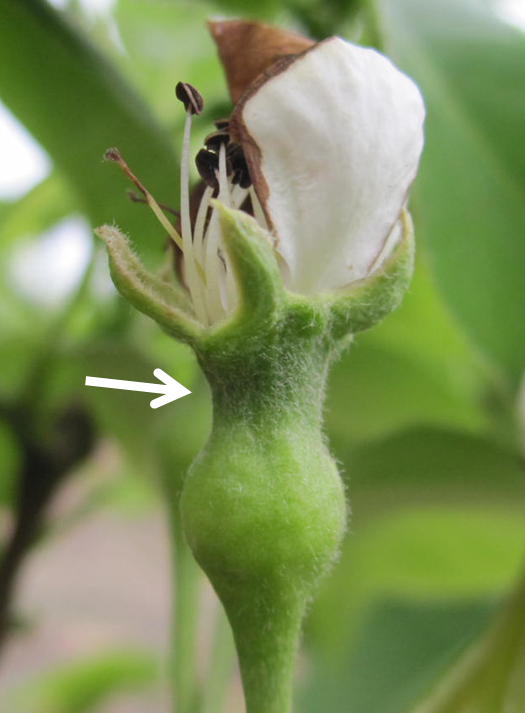
**

Supplement: Supplementary file 6 — Additional file 6: A pear fruit with calyx tube (white arrow) at 22 d after full bloom. (DOC 764 KB) [file 12864_2013_5444_MOESM6_ESM.doc]
