# Supplementary material for: Identifying the candidate genes involved in the calyx abscission process of 'Kuerlexiangli’ (Pyrus sinkiangensis Yu) by digital transcript abundance measurements
Source: BMC Genomics. 2013 Oct 23;14(1):727. doi: 10.1186/1471-2164-14-727 (PMC4046677; doi:10.1186/1471-2164-14-727)
Supplement: Supplementary file 7 — Additional file 7: Primer details for genes selected for quantitative real-time PCR analysis from results of digital transcript abundance measurements. This is the primer list of seven genes selected for quantitative real-time PCR assay to confirm the reliability of digital transcript abundance measurements. Gene ID, forward and reverse primers are shown. (DOC 30 KB) [file 12864_2013_5444_MOESM7_ESM.doc]

**Additional file 7: Primer details for genes selected for quantitative real-time PCR analysis from results of digital transcript abundance measurements.**

| Gene ID | Forward primer | Reverse primer |
| --- | --- | --- |
| Pbr041327.1 | CCTCAGACCCCTCACCAAGT | AGCTTTCACAACGCATGACC |
| Pbr036692.1 | GGTTCTTCGTTTCACTGCCC | GACCTGTTGCTCTTCCTCGG |
| Pbr021608.1 | TTGGCCTCTTCACCAAGTGT | GTGATTGCGAGGCAGAGTG |
| Pbr008092.1 | TCTTCGTGTTGTTCCTGCTCC | AACACCATCAAATCCATTAGCG |
| Pbr001279.1 | AGGGTTCTGGTTGCATCTCG | CAGTTTAGTGGGCACAGGGT |
| Pbr027181.1 | GGCCATCAAGACACTTGGTCTA | GTCACCACCGTTCCTCGTAG |
| Pbr000187.1 | GCCTGGCACCTATCAGACGA | ACCGGCAATGTTGGGAATC |
